# How the COVID-19 pandemic and related school closures reduce physical activity among children and adolescents in the WHO European Region: a systematic review and meta-analysis

**DOI:** 10.1186/s12966-023-01542-x

**Published:** 2023-12-19

**Authors:** Helena Ludwig-Walz, Waldemar Siemens, Sarah Heinisch, Indra Dannheim, Julika Loss, Martin Bujard

**Affiliations:** 1 Federal Institute for Population Research (BiB), Wiesbaden, Germany; 2 Institute for Evidence in Medicine, Medical Center - University of Freiburg, Faculty of Medicine, Freiburg, Germany; 3 Cochrane Germany, Cochrane Germany Foundation, Freiburg, Germany; 4 Institute for Physical Education and Sport, University of Education, Karlsruhe, Germany; 5 Regional Innovation Center for Health and Quality of Life in Fulda (RIGL), Fulda University of Applied Sciences, Fulda, Germany; 6 Department of Nutritional, Food and Consumer Sciences, Fulda University of Applied Sciences, Fulda, Germany; 7 Robert Koch Institute, Berlin, Germany; 8 Institute of Medical Psychology, Medical Faculty, University Heidelberg, Heidelberg, Germany

**Keywords:** Physical activity, Child, Adolescent, COVID-19, Exercise, Schools, Europe, Health policy, Evidence‐informed decision‐making, Non‐pharmacological interventions

## Abstract

**Background:**

Emerging research suggests that physical activity among children and adolescents decreased during the COVID-19 pandemic. However, a differentiated overview of European youth is lacking. In particular, no systematic analysis has been conducted to date on the impact of heterogeneous pandemic restrictions and school closures within European countries, and with regard to potentially vulnerable groups.

**Methods:**

We searched seven databases and included studies for children and adolescents (≤ 19 years) of the WHO European Region that compared physical activity during the COVID-19 pandemic with a pre-pandemic baseline using validated measurement instruments. We used the Oxford Stringency Index and School Closure Index as indicators of restriction stringency. Screening for eligibility, data extraction, assessment of the study risk of bias (using the ‘Risk of Bias in Non-randomized Studies - of Exposure’ [ROBINS-E]) and certainty grading of evidence (using the GRADE approach), were all done in duplicate. Unpublished data was requested from study authors. Data were pooled in random effects models. An a priori protocol was published, reporting is carried out in accordance with the ‘Preferred Reporting Items for Systematic Review and Meta-Analyses’ (PRISMA) statement.

**Results:**

Of 14,897 non-duplicate records, 26 publications (*n* = 15,038 pre-pandemic, *n* = 13,041 during pandemic) met full inclusion criteria. Comparison before and during the COVID-19 pandemic revealed a significant reduction in total physical activity (standardized mean difference [SMD], -0.57 [95%CI, -0.95; -0.20]) and moderate-to-vigorous physical activity (SMD, -0.43 [95% CI, -0.75; -0.10]), corresponding to a decrease of 12 min per day (a 20% reduction of the WHO recommendation). A decrease in sporting activity was also recorded. Subgroup analyses suggested that middle childhood (aged 8–12) and adolescents were particularly affected by the decline. School closures were associated with a reduction in physical activity. The certainty of evidence for all outcomes was low.

**Conclusions:**

A sharp decline in all forms of physical activity was recorded among European children and adolescents during the COVID-19 pandemic. This decline was higher during periods of school closure and mainly affected younger schoolchildren and adolescents. Immediate action by policy-makers and practitioners, as well as evidence-based public health strategies, are imperative in reversing this trend.

**Trial registration:**

PROSPERO: CRD42023395871

**Supplementary Information:**

The online version contains supplementary material available at 10.1186/s12966-023-01542-x.

## Background

The positive effects of physical activity on the physical and mental health of children and adolescents have been outlined in numerous studies [1, 2]. In particular, cardiovascular diseases, metabolic diseases, obesity and also mental health and cognition in youth all benefit from physical activity [1–5]. Furthermore, regular physical activity at a young age forms healthy habits in later life [6] and helps to reduce risk factors and diseases over the long term [7]. However, experts are keen to stress that the lack of adequate physical activity levels in children and adolescents is a major health problem [8, 9] that brings with it an enormous global health and economic burden [9, 10].

During the COVID-19 pandemic, opportunities for continuous physical activity among children and adolescents were severely limited by various public health and social measures (PHSM), e.g. closures of educational institutions (kindergartens, schools, universities), the restriction of access to physical activity opportunities (swimming, outdoor play, sports clubs) and the limiting of social contacts [11]. The effects of these limitations may contribute to long-term behavioural change in children and adolescents and could accelerate the downward-trend in physical activity [8] that is already in evidence and thereby have a severe lasting impact on the health of the upcoming generation [12]. Meanwhile, summary analyses describe a global decline in physical activity in children and adolescents during the COVID-19 pandemic [13–16]. However, there are important research gaps concerning the impact of the restriction stringency, school closures, different measurement tools and different types of physical activity. For the WHO European Region, a systematic analysis of changes in youth’s physical activity is lacking at all, although the number of studies is constantly increasing and the results are partly heterogeneous. The consideration of the WHO European Region further enables the analysis of country-specific heterogeneous PHSM to infer possible links to a change in physical activity in children and adolescents, creating a quasi-experimental design. Our aim, therefore, is to assess the impact that the COVID-19 pandemic has had on physical activity among children and adolescents in the WHO European Region compared with a pre-pandemic baseline, taking particular account of the relevance of restriction stringency policies.

## Methods

The systematic review and meta-analysis is reported according to the Preferred Reporting Items for Systematic Reviews and Meta-analyses (PRISMA) [17] statement (Additional file [AF1]: Table S1) and adheres to the Cochrane Handbook for Systematic Reviews [18]. It was registered on the International Prospective Register of Systematic Reviews (PROSPERO; CRD42023395871) [19] and an a priori protocol was published [20]. Deviations from the protocol are reported in AF1: Table S2.

### Eligibility criteria

We defined the following criteria as being eligible for inclusion: (1) Children and adolescents from the WHO European Region [21] ≤ 19years; (2) physical activity measurement at least once during the COVID-19 pandemic; (3) reporting of a pre-pandemic baseline; (4) measuring of physical activity with validated instruments; and (5) primary studies (also including pre-prints and congress abstracts) or reports (grey literature). We placed no restrictions on language or effect measures.

### Information sources and search strategy

We searched in seven electronic databases (PubMed, Embase, Sports Medicine & Education Index, PsycINFO, Web of Science, Cochrane Central Register of Controlled Trials [CENTRAL] and WHO COVID-19 Research Database [including pre-prints]) for eligible publications through to January 31, 2023. We tried to identify other potentially eligible publications by handsearching the reference lists of all included studies and related systematic reviews, and also searched for registered observational studies in clinicaltrials.gov. In addition, the data sources of the ‘Global Matrix 4.0 Physical Activity Report’ [16] and websites of key organizations (see AF1: Table S3) were checked.

We designed the search strategy by using validated or recommended search filters and conducted a peer-review process considering the evidence-based Peer Review of Electronic Search Strategies (PRESS) checklist [22] (see protocol [20] for further details). The search strategy for every database is presented in the Supplement (AF1: Table S4).

### Selection process

We began by performing an automated deduplication process with assistance from the EPPI reviewer software [23]. This was followed by title/abstract screening, conducted independently in reviewer teams of two (HLW, ID, SH). We obtained the full text of all potentially relevant records. Disagreements were resolved through consensus. We prepared a PRISMA flow diagram for study selection (AF1: Fig. S1). Reasons for the exclusion of publications following full-text assessment were also provided (AF1: Fig. S1 and Table S5).

### Data extraction

For studies meeting our inclusion criteria, reviewer teams of two (HLW, ID, SH) independently extracted key study characteristics in Table 1 ‘Characteristics of included studies’; disagreements regarding data extraction were resolved through discussion. For several publications, we requested further data via email from the authors and sent a reminder after 2 weeks if no response was received; eight authors provided us with additional, unpublished data. For three publications, the corresponding author could not be reached by email [24–26]. In the case of duplicate publications or multiple reports of a study, we compared and considered all available relevant data. We expanded our study characterization by adding the Oxford Stringency Index and the School Closure Index [11] for the measurement period of every study as policy indices for the classification of PHSM. The Oxford Stringency Index consists of nine variables; one of these variables represents school closures in the respective country. In compliance with the COVIDSurg Collaborative [27], we defined three cut-off points for the Oxford Stringency Index: light restrictions (index < 20), moderate lockdowns (index 20–60) and full lockdowns (index > 60). For the School Closure Index, we specified two cut-off points: no or few alterations compared with a pre-COVID-19 situation (index < 2) and partial or full school closure (index ≥ 2) [28]. More details on these indices are contained in the protocol [20].

**Table 1:** Characteristics of the studies included

**Study information**		**Population**		**Exposure**		**Comparison**	**Outcome**		**Risk of bias**
**First author, year**	**Study type,name of the study**	**Sample size (% female)**	**Age of study population**	**Time point during COVID-19 pandemic**	**Policy indices **[11]	**Time point of pre-pandemic baseline, link between measurement time points**	**Type of outcome, symptom reporter**	**Detailed description of diagnostic instrument**	
**Bosnia and Herzegovina**									
Geets Kesic, 2021 [24]	Cohort study	PP & DP: 859 (43)	Age range: 14 to 18y	4/2020	Oxford Stringency Index: 95.4 (89.8 to 96.3) School Closure Index: 3.0 (3.0 to 3.0) Days OSI > 60 before measurement: 12 Days of removed restrictions: 0	1/2020, same population	**Total** physical activity, self-reported	Name: PAQ for Adolescents (PAQ-A) Estimated time-frame: 7-day recall Cut-off points: NI	High
**Croatia**									
Sekulic, 2020 [9]	Cohort study	PP & DP: 388 (32.4)	Age range: 15 to 18y, Mean ± SD, 16.4 ± 1.9y	4/2020	Oxford Stringency Index: 91.8 (90.7 to 92.6) School Closure Index: 3.0 (3.0 to 3.0) Days OSI > 60 before measurement: 16 Days of removed restrictions: 0	9-10/2019, same population	**Total** physical activity, self-reported	Name: PAQ for Adolescents (PAQ-A) Estimated time-frame: 7-day recall Cut-off points: NI	Some concerns
Zenic, 2020 [10]	Cohort study	PP & DP: 823 (NI)	Age range: 11 to 17y Mean ± SD, 16.5 ± 2.1y	4/2020	Oxford Stringency Index: 95.4 (89.8 to 96.3) School Closure Index: 3.0 (3.0 to 3.0) Days OSI > 60 before measurement: 12 Days of removed restrictions: 0	10/2019 to 3/2020, same population	**Total** physical activity, self-reported	Name: PAQ for Adolescents (PAQ-A) Estimated time-frame: 7-day recall Cut-off points: NI	High
**Czech Republic**									
Štverá-ková, 2021 [29]	Cross-sectional study	PP: 206 (49) DP: 98 (57)	Age range: 8 to 12y Mean ± SD, 10.1 ± 1.5y	11/2020 – 1/2021	Oxford Stringency Index: 71.1 (62.0 to 81.5) School Closure Index: 2.6 (2.0 to 3.0) Days OSI > 60 before measurement: 10 Days of removed restrictions: 0	12/2019–1/2020, same population	**Total** physical activity, self-reported	Name: PAQ for Children (PAQ-C) Estimated time-frame: 7-day recall Cut-off points: NI	Some concerns
**Germany**									
Kurz, 2022 [30]	Cross-sectional study, Ulm SPATZ Health Study	PP: 296 (53) DP: 63 (51)	Age range: 6 to 7y	3-7/2020	Oxford Stringency Index: 64.8 (32.9 to 76.9) School Closure Index: 2.3 (2.0 to 3.0) Days OSI > 60 before measurement: 0 Days of removed restrictions: 0	9/2018–3/2020	**Total** physical activity, parent-reported	Name: Bayer-Questionnaire Estimated time-frame: NI Cut-off points: NI	High
Schmidt, 2020 [31]	Cohort study, German Motorik-Modul (MoMo)	PP & DP: 1,711 (49.8)	Age range: 4 to 17y PP: Mean ± SD, 10.4 ± 4.0y DP: Mean ± SD, 11.3 ± 4.1y	4-5/2020	Oxford Stringency Index: 76.9 (76.9 to 76.9) School Closure Index: 3.0 (3.0 to 3.0) Days OSI > 60 before measurement: 30 Days of removed restrictions: 0	8/2018–3/2020, same population	**Moderate-to-vigorous** physical and **sporting** activity, self-reported > 11y, parental support ≤ 11	Name: MoMo-PAQ Estimated time-frame: 7-day recall Cut-off points: NI	Some concerns
Schmidt, 2021 [32]	Cohort study, German Motorik-Modul (MoMo)	PP: 1,711 (49.8) DP: 1,483 (NI)	Age range: 4 to 17y	1-2/2021	Oxford Stringency Index: 83.3 (83.3 to 83.3) School Closure Index: 3.0 (3.0 to 3.0) Days OSI > 60 before measurement: 99 Days of removed restrictions: 0	8/2018–3/2020, same population	**Moderate-to-vigorous** physical and **sporting** activity, self-reported > 11y, parental support ≤ 11	Name: MoMo-PAQ Estimated time-frame: 7-day recall Cut-off points: NI	Some concerns
**Ireland**									
O’Kane, 2021 [33]	Retrospective study	PP: 281 (100) DP: 94 (100)	Age range: 12 to 14y PP&DP: Mean ± SD, 12.8 ± 0.8y	5–6/2020	Oxford Stringency Index: 77.7 (38.9 to 90.7) School Closure Index: 2.9 (2.0 to 3.0) Days OSI > 60 before measurement: 36 Days of removed restrictions: 0	Retrospective study	**Moderate-to-vigorous** physical activity, self-reported	Name: PACE+ Estimated time-frame: NI Cut-off points: NI	Some concerns
**Italy**									
Mastorci, 2021 [34]	Cohort study	PP & DP: 1,289 (51.7)	Age range: 10 to 14y Mean ± SD, 12.53 ± 1.25y	4/2020	Oxford Stringency Index: 93.5 (93.5 to 93.5) School Closure Index: 3.0 (3.0 to 3.0) Days OSI > 60 before measurement: 52 Days of removed restrictions: 0	9-10/2019	**Total** physical activity, self-reported	Name: PAQ for Children (PAQ-C) Estimated time-frame: 7-day recall Cut-off points: NI	Some concerns
Dallolio, 2022 [35]	Cohort study	PP: 77 (37.7) DP: 77 (37.7)	PP: Mean ± SD, 7.84 ± 1.41y DP: Mean ± SD, 9.19 ± 3.00y	1/2021	Oxford Stringency Index: 76.6 (74.1 to 82.4) School Closure Index: 2.0 (2.0 to 2.0) Days OSI > 60 before measurement: 313 Days of removed restrictions: 0	10/2019	**Total** physical activity, self-reported	Name: PAQ for Children (PAQ-C) Estimated time-frame: 7-day recall Cut-off points: NI	Some concerns
							**Moderate-to-vigorous** physical activity, accelerometer	Name: ActiGraph wGT3X-BT Estimated time-frame: 5 weekdays and 2 weekend days Cut-off points: According to Evenson et al. [36] (light, moderate, vigorous)	
**Netherlands**									
ten Velde, 2021 [37]	Cohort study	PP & DP: 64 (66.7)	Age range: 7 to 12y PP&DP: Mean ± SD, 10.1 ± 0.7y	6/2020	Oxford Stringency Index: 61.0 (59.3 to 63.0) School Closure Index: 1.5 (1.0 to 3.0) Days OSI > 60 before measurement: 46 Days of removed restrictions: 0	5-6/2019	**Moderate-to-vigorous** physical activity, accelerometer	Name: ActiGraph GT3X Estimated time-frame: NI Cut-off points: NI	Some concerns
**Poland**									
Kołota, 2021 [38]	Retrospective study	PP & DP: 1,334 (53.3)	Age range: 10 to 16y	6/2020	Oxford Stringency Index: 52.6 (50.9 to 64.8) School Closure Index: 2.0 (2.0 to 2.0) Days OSI > 60 before measurement: 62 Days of removed restrictions: 0	Retrospective study	**Moderate-to-vigorous** physical activity, self-reported	Name: Question regarding number of days with > 60 min of MVPA, including “increases your heart rate and makes you get out of breath some of the time” Estimated time-frame: week Cut-off points: MVPA ≥ 3	High
Łuszczki, 2021 [39]	Cross-sectional study	PP: 376 (50.3) DP: 640 (51.3)	Age range: 6 to 15y PP: Mean ± SD, 10.51 ± 2.13y DP: Mean ± SD, 10.79 ± 2.02y	2–3/2021	Oxford Stringency Index: 71.5 (71.3 to 73.2) School Closure Index: 2.1 (2.0 to 3.0) Days OSI > 60 before measurement: 100 Days of removed restrictions: 0	2-3/2020	**Moderate-to-vigorous** physical activity, self- and parent-reported	Name: Question regarding number of days with > 60 min of MVPA, including “increased your breathing rate” Estimated time-frame: last week Cut-off points: NI	High
**Portugal**									
Mercê, 2022 [40]	Retrospective study	PP & DP: 61 (NI)	Age range: 5 to 17y Mean ± SD, 12 ± 3.1y	5-6/2020	Oxford Stringency Index: 65.0 (59.3 to 88.0) School Closure Index: 2.3 (2.0 to 3.0) Days OSI > 60 before measurement: 44 Days of removed restrictions: 0	Retrospective study	**Total** physical activity, self-reported	Name: Pictorial Children’s Physical Activity Questionnaire Estimated time-frame: weekday and weekend Cut-off points: NI	Very high
**Slovenia**									
Blazević, 2021 [41]	Cohort study	PP & DP: 209 (57)	Age range: 15 to 17y Mean ± SD, 16.4 ± 1.9y	4/2020	Oxford Stringency Index: 96.3 (96.3 to 96.3) School Closure Index: 3.0 (3.0 to 3.0) Days OSI > 60 before measurement: 16 Days of removed restrictions: 0	9-10/2019	**Total** physical activity, self-reported	Name: PAQ for Adolescents (PAQ-A) Estimated time-frame: 7-day recall Cut-off points: NI	Some concerns
Morrison, 2021 [42]	Cross-sectional study	PP & DP: 62 (50)	PP: Mean ± SD, 11.6 ± 1.5y DP: Mean ± SD, 11.5 ± 1.5y	4/2020	Oxford Stringency Index: 88.1 (75.0 to 89.8) School Closure Index: 3.0 (3.0 to 3.0) Days OSI > 60 before measurement: 13 Days of removed restrictions: 0	10/2018	**Moderate-to-vigorous** physical activity, self-reported	Name: School Health Action, Planning, and Evaluation System (SHAPES) Estimated time-frame: weekday Cut-off points: NI	Some concerns
**Spain**									
Alonso-Mart¡nez, 2021 [43]	Cohort study	PP: 21 (42.9) DP: 21 (42.9)	Age range: 4 to 6y PP: Mean ± SD, 4.28 ± 0.80y DP: Mean ± SD, 4.29 ± 0.76y	3-4/2020	Oxford Stringency Index: 68.2 (11.1 to 85.2) School Closure Index: 2.6 (0.0 to 3.0) Days OSI > 60 before measurement: 0 Days of removed restrictions: 0	9-12/2019	**Total** and **moderate-to-vigorous** physical activity, accelerometer	Name: GENEActiv tri-axial accelerometer Estimated time-frame: 3 days prior to lockdown and 3 days during lockdown Cut-off points: According to Crotti et al. [44] (light, moderate, vigorous)	Very high
García-Alonso, 2022 [45]	Cohort study	PP: 124 (50) DP: 110 (51)	Age range: 4 to 7y Mean, 5.77y	1-3/2021	Oxford Stringency Index: 70.6 (66.7 to 78.7) School Closure Index: 1.1 (1.0 to 3.0) Days OSI > 60 before measurement: 68 Days of removed restrictions: 0	1-3/2020	**Total** and **moderate-to-vigorous** physical activity, accelerometer	Name: GENEActiv tri-axial accelerometer Estimated time-frame: 6 consecutive days Cut-off points: According to Hildebrand et al. [46, 47] (light, moderate, vigorous)	Some concerns
Medrano, 2021 [48]	Cohort study	PP: 291 (47.8) DP: 113 (48.7)	Age range: 8 to 16y PP: Mean ± SD, 12.1 ± 2.4y DP: Mean ± SD, 12.0 ± 2.6y	3-4/2020	Oxford Stringency Index: 68.2 (11.1 to 85.2) School Closure Index: 2.6 (0.0 to 3.0) Days OSI > 60 before measurement: 0 Days of removed restrictions: 0	9-12/2019	**Total** and **moderate-to-vigorous** physical activity, accelerometer	Name: ActiGraph (no further information) Estimated time-frame: NI Cut-off points: NI	High
Tapia-Serrano, 2022 [49]	Cross-sectional study	PP: 844 (42.7) DP: 501 (55.3)	Age range: 11 to 16y PP: Mean ± SD, 13.12 ± 0.86y DP: Mean ± SD, 14.39 ± 1.16y	2-3/2021	Oxford Stringency Index: 69.5 (66.7 to 71.3) School Closure Index: 1.0 (1.0 to 1.0) Days OSI > 60 before measurement: 201 Days of removed restrictions: 0	3-6/2018	**Total** physical activity, self-reported	Name: PAQ for Adolescents (PAQ-A) Estimated time-frame: 7-day recall Cut-off points: NI	Some concerns
**Sweden**									
Chen, 2022 [50]	Cohort study	PP & DP: 583 (NI)	PP: Mean ± SD, 13.6 ± 0.4y	2-11/2020	Oxford Stringency Index: 69.5 (66.7 to 71.3) School Closure Index: 1.0 (1.0 to 1.0) Days OSI > 60 before measurement: 201 Days of removed restrictions: 0	9/2015–6/2019	**Moderate-to-vigorous** physical activity, self-reported	Name: WHO HBSC physical activity questionnaire Estimated time-frame: NI Cut-off points: NI	Some concerns
**Switzerland**									
Zehnder, 2022 [51]	Retrospective study	PP & DP: 237 (44.1)	Age range: 7 to 16y Mean ± SD, 11.7 ± 2.47y	4-5/2020	Oxford Stringency Index: 69.5 (66.7 to 71.3) School Closure Index: 1.0 (1.0 to 1.0) Days OSI > 60 before measurement: 201 Days of removed restrictions: 0	Retrospective study	**Sporting** activity, self-reported	Name: German Physical Activity, Exercise and Sport Questionnaire (BSA-F) Estimated time-frame: PP: Last 7 days, DP: typical week Cut-off points: NI	High
**United Kingdom**									
Bingham, 2021 [52]	Cohort study	PP: 643 (49) DP: 658 (NI)	Age range: 9 to 13y PP: Mean ± SD, 9.1 ± 1.1y DP: Mean ± SD, 10.5 ± 1.1y	5-7/2020	Oxford Stringency Index: 68.5 (64.4 to 73.2) School Closure Index: 3.0 (3.0 to 3.0) Days OSI > 60 before measurement: 40 Days of removed restrictions: 0	2017–2020	**Total** physical activity, self-reported	Name: PP: PAQ for Children (PAQ-C); DP: Youth Activity Profile - English Youth Version (YAP) Estimated time-frame: PAQ-C: 7-day recall; YAP: normal weekday or weekend in the last 7 days Cut-off points: PAQ-C: girls: sufficiently active > 2.7 aggregate score, not sufficiently active < 2.7 aggregate score; boys: sufficiently active > 2.9 aggregate score, not sufficiently active < 2.9 aggregate score; YAP: sufficiently active > 60 min, not sufficiently active < 60 min	High
James, 2021 [53]	Cross-sectional study	PP1: 475 (50.7) PP2: 1,150 (47.7) DP: 1,068 (49.4)	Age range: 8 to 11y PP1: Mean, 10.30y PP2: Mean, 10.27y DP: Mean, 9.99y	4-6/2020	Oxford Stringency Index: 74.9 (67.6 to 79.6) School Closure Index: 3.0 (3.0 to 3.0) Days OSI > 60 before measurement: 10 Days of removed restrictions: 0	PP1: 3-6/2018 PP2: 3-6/2019	**Moderate-to-vigorous** physical activity, self-reported	Name: HAPPEN survey Estimated time-frame: Last 7 days Cut-off points: NI	Some concerns
Salway, 2022 [54]	Cross-sectional study	PP: 1,296 (52) DP: 393 (49)	Age range: 10 to 11y PP: Mean ± SD, 11.0 ± 0.4y DP: Mean ± SD, 10.8 ± 0.5y	5-12/2021	Oxford Stringency Index: 48.3 (41.2 to 62.5) School Closure Index: 1.0 (1.0 to 2.0) Days OSI > 60 before measurement: 405 Days of removed restrictions: 0	3/2017–7/2018	**Moderate-to-vigorous** physical activity, accelerometer	Name: ActiGraph wGT3X-BT Estimated time-frame: 5 weekdays and 2 weekend days Cut-off points: According to Evenson et al. [36] (light, moderate, vigorous)	Some concerns
Sheldrick, 2022 [55]	Cohort study	PP: 102 (50.0) DP: 102 (50.0)	Age range: 10 to 12y PP: Mean ± SD, 10.2 ± 0.7y DP: Mean ± SD, 12.8 ± 0.8y	6-7/2020	Oxford Stringency Index: 68.1 (64.4 to 73.2) School Closure Index: 3.0 (3.0 to 3.0) Days OSI > 60 before measurement: 71 Days of removed restrictions: 0	2017–18	**Total** and **moderate-to-vigorous** physical activity, accelerometer	Name: ActiGraph GT9X Estimated time-frame: PP: 7 consecutive days; DP: 8 consecutive days Cut-off points: According to Chandler et al. [56] (TPA ≥ 306 counts/5 secs, MVPA ≥ 818 counts/5 secs)	Some concerns

Policy indices: 95% confidence intervals are in parentheses

*DP* During pandemic, *MoMo* German Motorik-Modul, *MVPA* Moderate-to-vigorous physical activity, *NI* No information, *OSI* Oxford Stringency Index, *PACE* Patient-Centered Assessment and Counseling for Exercise Plus Nutrition, *PAQ* Physical Activity Questionnaire, *PP* Pre-pandemic, *SA* Sporting activity, *SD* Standard deviation, *TPA* Total physical activity, *y* Years

We defined total physical activity (TPA), moderate-to-vigorous physical activity (MVPA) and sporting activity (SA) as primary outcomes. Validation of the measurement instrument used, including both self-reported and device-based measurements, was defined as a prerequisite. No limitations were set as regards effect measures.

### Risk of bias assessment

All studies were independently assessed by two reviewers (HLW, SH), using the ‘Risk of Bias (RoB) in Non-randomized Studies - of Exposure’ (ROBINS-E) instrument. This tool comprises seven assessment criteria, with the RoB judgements expressed as ‘low RoB’, ‘some concerns RoB’, ‘high RoB’ or ‘very high RoB’ [57]; more details are provided in the protocol [20]. The studies were subsequently grouped into ‘some concerns RoB’ and ‘high RoB’ (including the categories ‘high RoB’ and ‘very high RoB’); no study received the rating ‘low RoB’. Interpretation of studies with ‘some concerns RoB’ was given preference in meta-analyses to deal with methodological heterogeneity and potential confounding.

### Synthesis methods

For all of the studies that were included, we provide both the effect estimates at pre-pandemic and pandemic measurement and the change effect as standardized mean difference (SMD) or risk ratio with the corresponding 95% confidence interval (CI). We performed meta-analysis when data from at least three studies with different study populations could be pooled. First, we distinguished between TPA, MVPA and SA and pooled available data sets using SMD (95% CI) to summarize change estimates.

Second, we differentiated according to the measurement instrument used (accelerometer measurement versus self-reported scores). Device-based measurements (via accelerometer) were summarized as ‘minutes/day’ (details for data conversion are presented in AF1: Table S6). Self-reported measurements for TPA were summarized within the Physical Activity Questionnaire for Children/Adolescents (PAQ-C/A) since the majority of measurements used this instrument. Due to the heterogeneity in self-reported MVPA measurements, we summarized these measurements as SMD and subsequently re-expressed them using a familiar instrument (WHO Health Behaviour in School-aged Children [HBSC survey]), to ensure practical interpretability of the results.

Third, we analyzed change effect estimates for the subgroups: gender (female/male), age (age categories are based on those laid down by the Centers for Disease Control and Prevention [58]: ‘preschoolers/middle childhood’: 3 to 8 years; ‘middle childhood’: 9–11 years; and ‘young teens/teenagers’: 12 to 18 years, studies with overlapping age intervals were assigned based on the age structure that was most appropriate and studies in which there was a wide age interval were excluded from these analyses), measurement time point (spring/summer 2020, winter 2020/2021, spring 2021), Oxford Stringency Index (≤ 60 versus > 60), School Closure Index (< 2 versus ≥ 2) and length of pandemic-related restrictions before measurement (Oxford Stringency Index > 60 before measurement for 30/60/90 days).

We performed some data conversion before conducting meta-analyses (AF1: Table S6). If the studies that were included did not report sufficient data for inclusion in the meta-analysis (e.g. reporting percentage change) and we had not received the information we had requested from the authors, the results were reported in narrative tables. Where possible, we included adjusted effect estimates. If both self-reported and parent-reported data were available, we included the self-reported data.

We assessed heterogeneity by visual inspection of the forest plots, the I^2^ statistic [59] and with 95% prediction intervals when > 3 studies were included in meta-analyses [60–62]. We considered I^2^ values of greater than 50% as substantial. We tried to explain heterogeneity by conducting subgroup analyses and meta-regression (if ≥ 10 studies per examined variable) [60] with the potential categorical moderators: RoB, age, symptom reporter, country, Oxford Stringency Index (≤ 60 versus > 60), School Closure Index (< 2 versus ≥ 2) and study design. In addition, the following potential continuous moderators were considered: time of measurement during pandemic, publication year, Stringency Index, School Closure Index and sample size. We considered potential publication bias by conducting a visual inspection of (contour-enhanced) funnel plots [63, 64] and we applied the Egger’s test when a meta-analysis included ≥ 10 studies [65].

We conducted meta-analysis calculations with the package ‘meta’ [66] in R Studio 4.2.1 [67] using the random effects model with a restricted maximum likelihood approach [68] and the Hartung-Knapp method for calculating the 95% CI. All statistical analyses were performed based on the statistical guidelines presented in the Cochrane Handbook for Systematic Reviews of Interventions [69].

### Certainty of evidence assessment

We applied the ‘Grading of Recommendations Assessment, Development and Evaluation’ (GRADE) approach, adapted to the use of non-randomized studies [70], to assess overall certainty of evidence for each of the primary outcomes; more information is provided in the protocol [20]. Certainty of evidence for each outcome was evaluated independently by two review authors (HLW, WS); differences were resolved through discussion. The ‘Summary of findings’ table summarizes the results regarding certainty of evidence. Details of the criteria used to grade the evidence are reported in AF1: Table S7; evidence profiles containing more detailed explanations can be found in AF1: Table S8.

## Results

Our systematic literature search identified 14,891 non-duplicate records and six grey literature publications. Of these, 135 studies and six grey literature reports were assessed for eligibility (full-text screening) and 25 [24, 25, 29–31, 33–35, 37, 39–43, 45, 48–55, 71, 72] studies and one report [32] were deemed to meet the criteria for inclusion in the review (AF1: Fig. S1). In total, data from 15,038 children and adolescents pre-pandemic and 13,041 children and adolescents during pandemic were included in this review. The most relevant reasons for exclusion after full-text screening were ‘no validation of the measurement instrument’ (*n* = 80, 59.3%); and ‘no data reporting on physical activity’ (*n* = 19, 14.1%); details are described in AF1: Table S5.

### Study characteristics

A detailed description of the included publications is presented in Table 1 and AF1: Table S9. The included 26 publications are scattered across 14 WHO European Region countries: four from Spain [43, 45, 48, 49], four from the United Kingdom [52–55], three from Germany [30, 31, 73], two from Croatia [25, 71], two from Italy [34, 35], two from Poland [39, 72], two from Slovenia [41, 42], and one each from Bosnia and Herzegovina [24], Czech Republic [29], Ireland [33], Netherlands [37], Portugal [40], Sweden [50] and Switzerland [51]. A graphical overview of how these studies are distributed is provided in AF1: Fig. S2. TPA and MVPA were analyzed in 15 publications (TPA: [24, 25, 29, 30, 34, 35, 40, 41, 43, 45, 48, 49, 52, 55, 71], MVPA: [31, 33, 35, 37, 39, 42, 43, 45, 48, 50, 53–55, 72, 73]) respectively, and SA in three publications [31, 51, 73]. Self-reported data were collected in 20 analyses [24, 25, 29–31, 33–35, 39–42, 49–53, 71–73] and accelerometer data in six analyses [35, 37, 43, 45, 48, 55].

The publications were conducted as cohort (*n* = 15, [24, 25, 31, 34, 35, 37, 41, 43, 45, 48, 50, 52, 55, 71, 73]), cross-sectional (*n* = 7, [29, 30, 39, 42, 49, 53, 54]) or retrospective studies (*n* = 4, [33, 40, 51, 72]). The majority were carried out in spring/summer 2020 (*n* = 18, [24, 25, 30, 31, 33, 34, 37, 40–43, 48, 51–53, 55, 71, 72]) or winter 2020/2021 (*n* = 6, [29, 35, 39, 45, 49, 73]). In 24 publications, the period during the pandemic was classified as ‘full lockdown’ (Oxford COVID-19 Stringency Index > 60, [24, 25, 29–31, 33–35, 37, 39–43, 45, 48–53, 55, 71, 73]). In 20 publications, pandemic-measurement occurred during partial or full school closures (School Closure Index ≥ 2, [24, 25, 29–31, 33–35, 39–43, 48, 52, 53, 55, 71–73]). The length of a ‘full lockdown’ (Oxford COVID-19 Stringency Index > 60) before pandemic measurement ranged from 0 to 405 days. The RoB assessment revealed ‘some concerns’ for 16 publications [25, 29, 31, 33–35, 37, 41, 42, 45, 49, 50, 53–55, 73], ‘high RoB’ for eight publications [24, 30, 38, 39, 48, 51, 52, 71] and ‘very high RoB’ for two publications [40, 43]. Details on RoB assessment are presented in AF1: Fig. S3 (traffic-light plots) und AF1: Fig. S4 (weighted bar plots).

### Meta-analysis for total physical activity

**Fig. 1:**
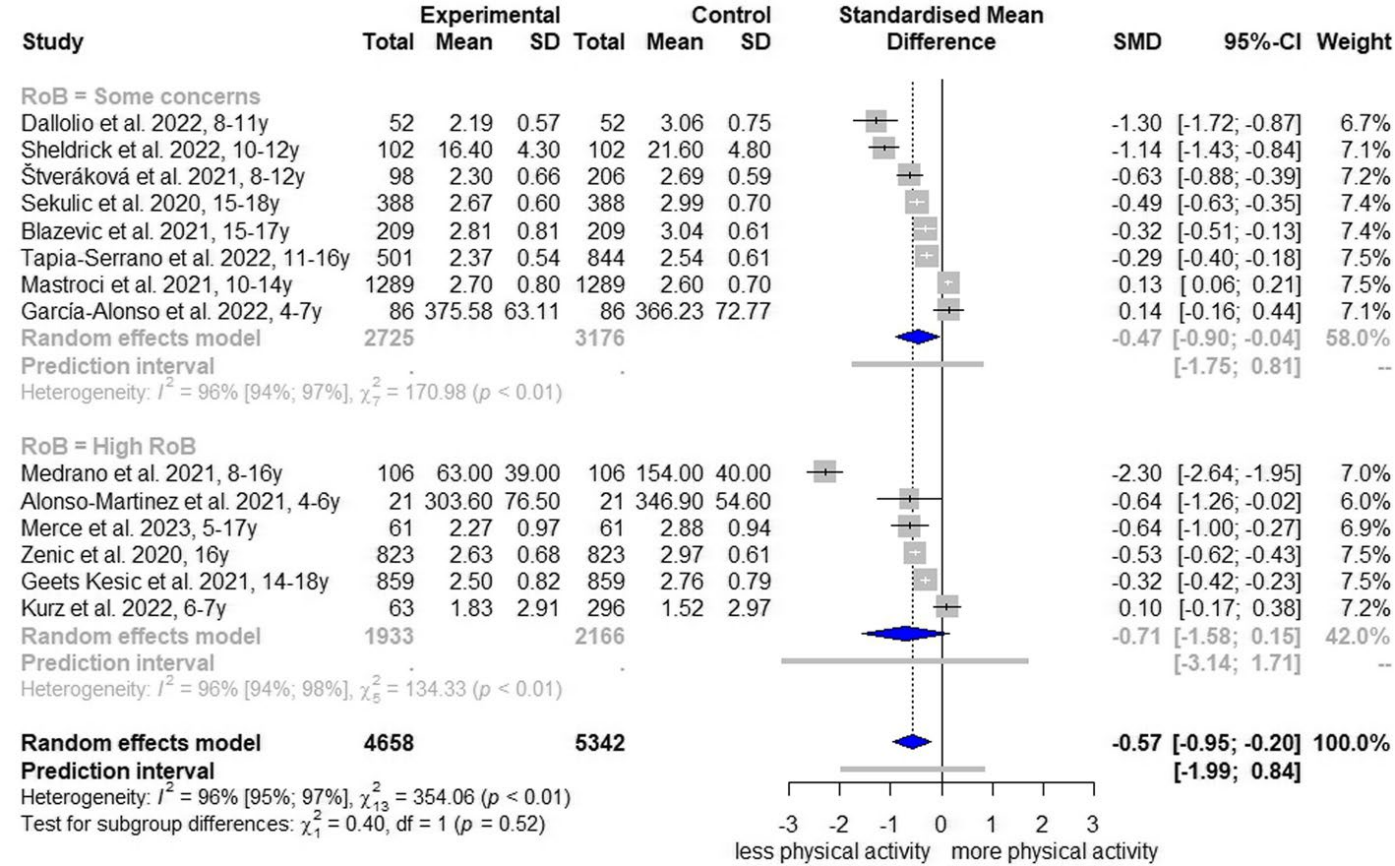
Forest plot of changes in total physical activity comparing before and during COVID-19 pandemic

For TPA, we performed a meta-analysis with 14 studies [24, 25, 29, 30, 34, 35, 40, 41, 43, 45, 48, 49, 55, 71] and certainty of evidence was graded as ‘low’ (Table 2). The pooled SMD estimate for change of TPA, including self-reported scores and accelerometer measurements, was -0.57 (95% CI, -0.95 to -0.20; I^2^ = 96%; Fig. 1) for all 14 studies, and a SMD of -0.47 (95% CI, -0.90 to -0.04; I^2^ = 96%; Fig. 1) for eight studies with a ‘some concerns RoB’ rating. The SMD for ‘high RoB’ studies had a wide 95% CI and crossed the null effect (-0.71, 95% CI -1.58 to 0.15; I^2^ = 96%).

**Table 2:** Summary of findings

**Outcome**	**Number of studies**	**Standardized mean difference, 95% CI**	**Summary of findings**	**Certainty of evidence (GRADE)**
**Total physical activity**	14 studies [24, 25, 29, 30, 34, 35, 40, 41, 43, 45, 48, 49, 55, 71]	All studies: -0.57, -0.95 to -0.20 ‘Some concerns RoB’ studies: -0.47, -0.95 to -0.20	Total physical activity among children and adolescents in Europe decreased significantly during the COVID-19 pandemic. Analysis revealed indications that stringent school closures (partially or fully closed schools) were associated with a higher decline in TPA compared with schools where there were either no restrictions or only a small number of restrictions. Since 47% of the studies had a high or very high RoB, a high degree of heterogeneity was present and a publication bias can be assumed, we downgraded the results to ‘low certainty of evidence’.	⊕⊕⊝⊝ **Low**^a,b,c^
**Moderate-to-vigorous physical activity**	12 publications [31–33, 35, 37, 39, 42, 43, 45, 50, 54, 55] (data from two publications [31, 32] of the same study population with different measuring time points were aggregated)	All studies: -0.43, -0.75 to -0.10 ‘Some concerns RoB’ studies: -0.43, -0.84 to -0.02	Moderate-to-vigorous physical activity among children and adolescents in Europe decreased significantly during the COVID-19 pandemic. Analysis revealed indications that stringent school closures (partially or fully closed schools) were associated with a higher decline in MVPA compared with schools where there were either no restrictions or only a small number of restrictions. Due to the high degree of heterogeneity and an indicated publication bias, we downgraded the results to ‘low certainty of evidence’.	⊕⊕⊝⊝ **Low**^b,d^
**Sporting activity**	3 publications [31, 32, 51]	No pooling occurred	Sporting activity decreased significantly in each of the three publications. As one of the three studies had a high RoB and inconsistency of the data can be suspected, we downgraded the results to ‘low certainty of evidence’.	⊕⊕⊝⊝ **Low**^e,f^

^a^Downgraded by -0.5 points due to 47% studies having a high or very high risk of bias

^b^Downgraded by -1 point due to considerable heterogeneity (total physical activity: 96%, moderate-to-vigorous physical activity: 92%; wide 95% prediction intervals)

^c^Downgraded by -0.5 points due to visual inspection of the funnel plot suggesting asymmetry and being supported by an almost statistically significant test (*p* = 0.052)

^d^Downgraded by -1 point due to visual inspection of the funnel plot suggesting asymmetry and being supported by a statistically significant test (*p* = 0.02)

^e^Downgraded by -1 due to one of the three studies having a high risk of bias and two studies being from the same population

^f^Downgraded by -1 due to differences in point estimate and no overlap of 95% CI

Hereinafter, analyses were differentiated according to the outcome measurement instrument. Eight studies with the widely used PAQ-C/A instruments yielded a reduction of -0.29 score points (95% CI, -0.51 to -0.08; I^2^ = 96%; AF1: Fig. S5). A pooling of four studies with an accelerometer measurement revealed a reduction of -47.7 min (95% CI, -115.9 to 20.5; I^2^ = 96%; AF1: Fig. S6) per day.

**Fig. 2:**
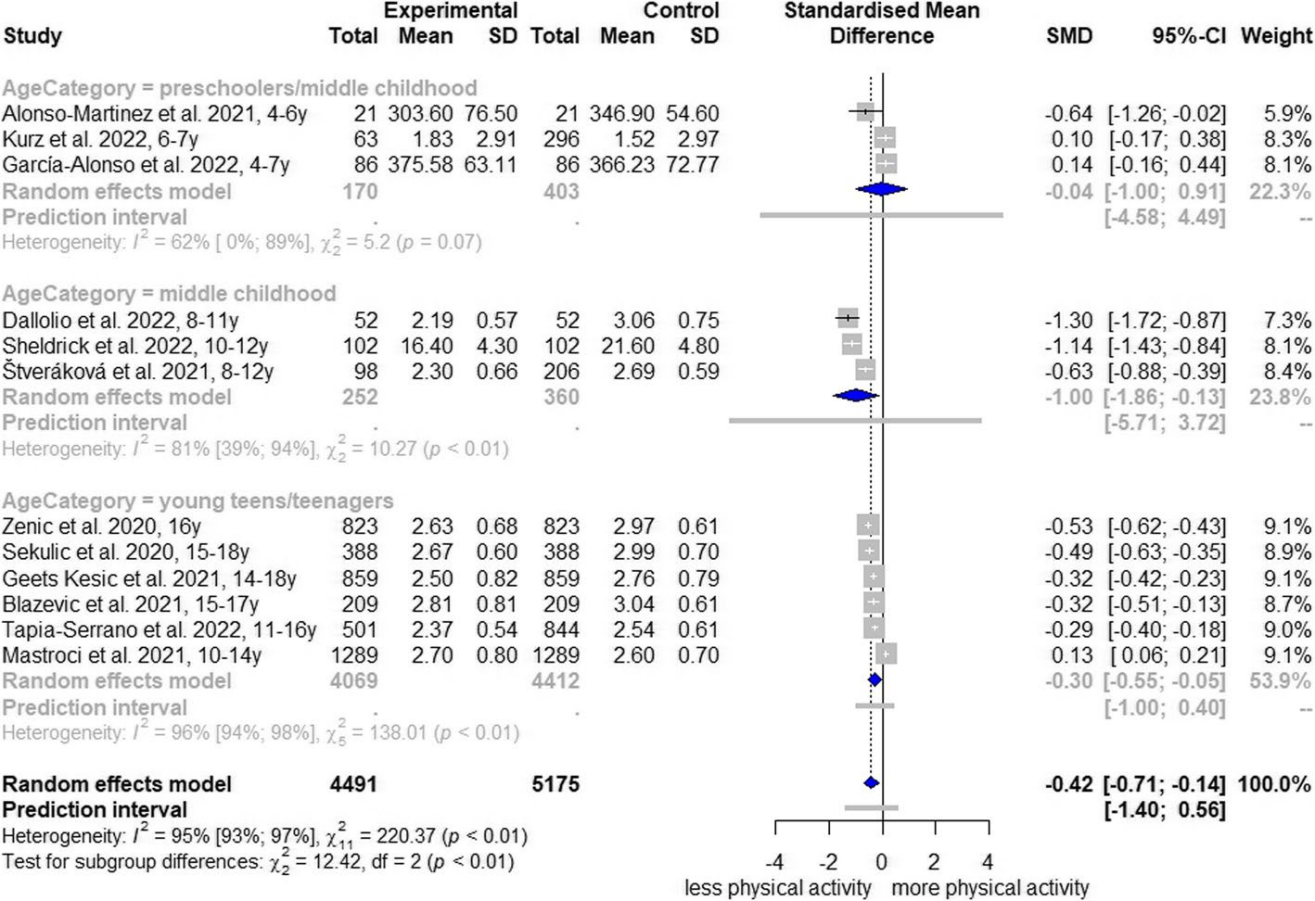
Forest plot of changes in total physical activity comparing different age groups

Gender-stratified pooling yielded a SMD of -0.16 (95% CI, -0.46 to 0.15; I^2^ = 84%; AF1: Fig. S7) for female children and adolescents and a SMD of -0.37 (95% CI, -0.81 to 0.08; I^2^ = 86%; AF1: Fig. S7) for male CA. The age-group classification showed a significant decline for middle childhood (adapted to the age range of 8 to 12 years: SMD, -1.00; 95% CI, -1.86 to -0.13; I^2^ = 81%; Fig. 2) and young teens/teenagers (SMD, -0.30; 95% CI, -0.55 to -0.05; I^2^ = 96%; Fig. 2), but not for children younger than 7 years of age (SMD, -0.04; 95% CI, -1.00 to 0.91; I^2^ = 62%; Fig. 2).

**Fig. 3:**
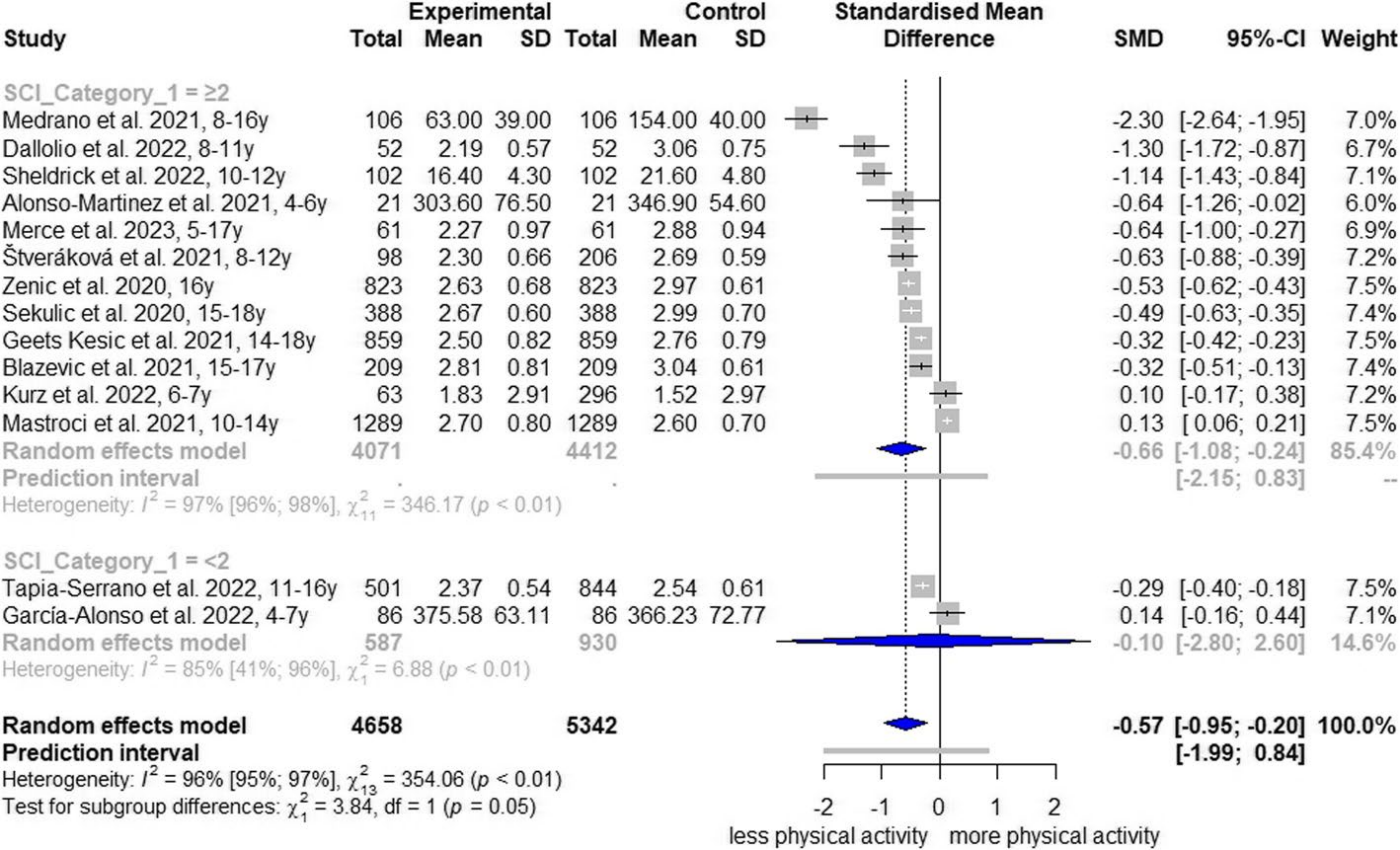
Forest plot of changes in total physical activity comparing school closure indices

Regarding the course of time, TPA decreased in spring/summer 2020 (10 studies: SMD, -0.60; 95% CI, -1.10 to -0.11; I^2^ = 97%; AF1: Fig. S8), in winter 2020/spring 2021 (3 studies: SMD, -0.59; 95% CI, -2.36 to 1.18; I^2^ = 94%; AF1: Fig. S8) and in spring 2021 (1 study: SMD, -0.29; 95% CI, -0.40 to -0.18; AF1: Fig. S8). A comparison regarding the Oxford Stringency Index was not possible because all studies had an index > 60 at the measurement time point. Comparisons of the School Closure Index revealed that full or partial school closures were associated with higher TPA reductions (SCI ≥ 2: SMD, -0.66; 95% CI, -1.08 to -0.24; I^2^ = 97%; Fig. 3), whereas no school closure or few alterations had no statistical association with TPA reductions (SMD, -0.10; 95% CI, -2.80 to 2.60; I^2^ = 85%; Fig. 3).

Although the analyses by restriction length (number of days before measurement in which the Oxford Stringency Index was > 60) revealed no significant associations, the trend indicated that TPA decreases more where the duration of the restriction is longer (Restriction before measurement ≥ 30 days: SMD, -0.48; 95% CI, -1.32 to 0.37; I^2^ = 97%; Fig. S9; Restriction before measurement ≥ 60 days: SMD, -0.63; 95% CI, -1.72 to 0.45; I^2^ = 95%; Fig. S10; Restriction before measurement ≥ 90 days: SMD, -0.77; 95% CI, -7.16 to 5.61; I^2^ = 95%; Fig. S11).

### Meta-analysis for moderate-to-vigorous physical activity

In the meta-analysis for MVPA, we include 12 publications [31–33, 35, 37, 39, 42, 43, 45, 50, 54, 55] (data from two publications [31, 32] of the same study population with different measuring time points were aggregated) and certainty of evidence was rated as ‘low’ (AF1: Table S8). A SMD of -0.43 (95% CI, -0.75 to -0.10; I^2^ = 92%; AF1: Fig. S12) was calculated as the total change effect, while pooling of ‘some concerns RoB’ resulted in a SMD of -0.43 (95% CI, -0.84 to -0.02; I^2^ = 94%; AF1: Fig. S12).

Self-reported changes revealed a reduction of -0.55 score points when re-expressed with the WHO HBSC survey instrument based on the SD (= 1.9) from Chen et al. [50] (AF1: Fig. S13). Changes based on six accelerometer measurements resulted in a MVPA reduction of -12.0 min (95% CI, -27.1 to 3.1; I^2^ = 96%; AF1: Fig. S14) per day.

**Fig. 4:**
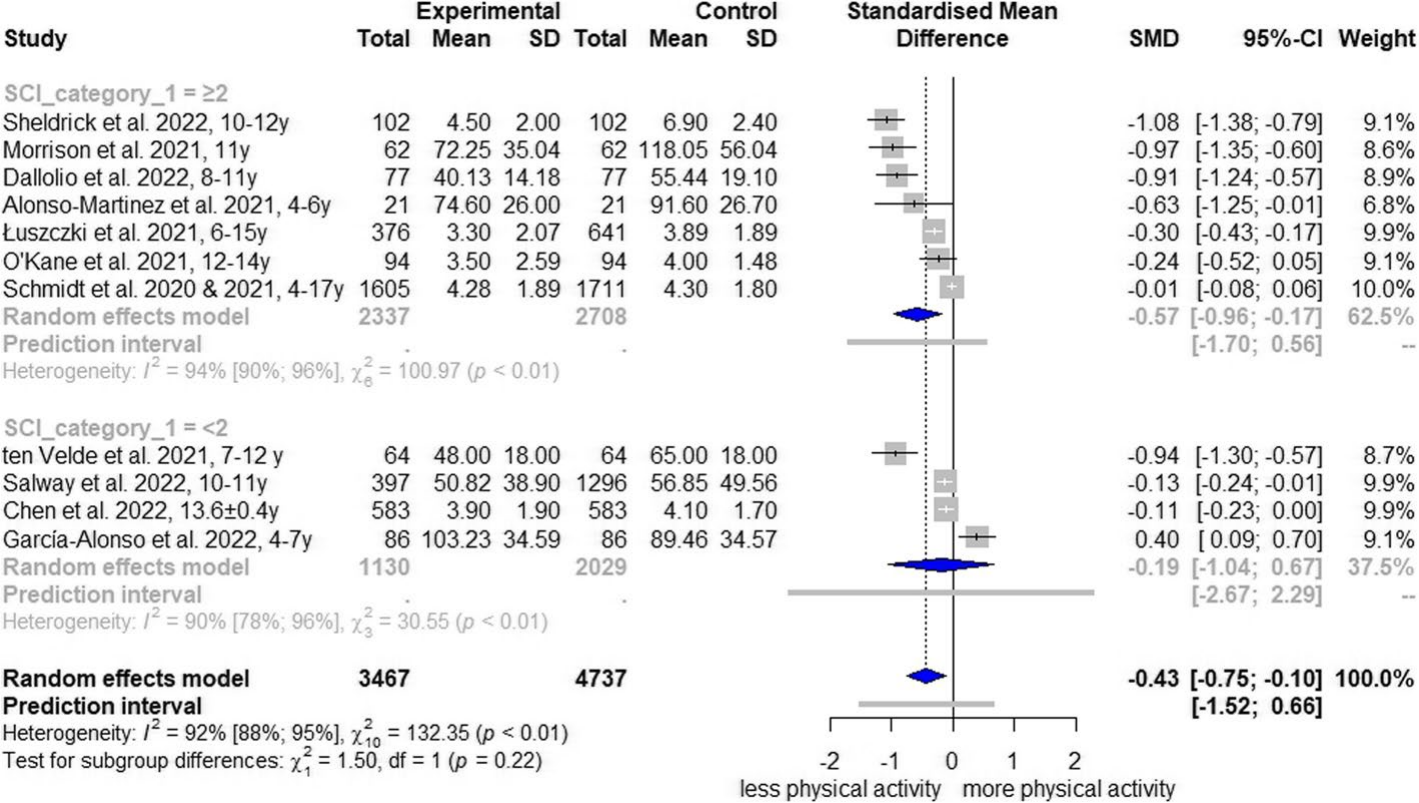
Forest plot of changes in moderate-to-vigorous physical activity comparing school closure indices

Subgroup analysis by gender revealed a SMD of -0.15 (95% CI, -0.48 to 0.18; I^2^ = 78%; AF1: Fig. S15) for female children and adolescents and a SMD of -0.33 (95% CI, -1.01 to 0.35; I^2^ = 90%; AF1: Fig. S15) for male children and adolescents regarding MVPA reduction. Stratification by age groups yielded a reduction, with a SMD of -0.74 (95% CI, -1.45 to -0.04; I^2^ = 95%; AF1: Fig. S16) for middle childhood, while change effect estimates for preschoolers and young teens/teenagers were imprecise and the 95% CI crossed the null effect (preschoolers: SMD, -0.08; 95% CI, -6.60 to 6.44; I^2^ = 88%; young teens/teenagers: SMD, -0.42; 95% CI, -1.56 to 0.72; I^2^ = 89%; Fig. S16). The analysis over time indicates a reduction in spring/summer 2020 (6 studies: SMD, -0.59; 95% CI, -1.14 to -0.04; I^2^ = 97%; AF1: Fig. S17) and winter 2020/spring 2021 (4 studies: SMD, -0.26; 95% CI, -1.09 to 0.57; I^2^ = 91%; AF1: Fig. S17); two studies were excluded from this analysis because measurement periods were too broad [50, 54]. Only comparisons regarding the School Closure Index were possible. In measurement periods with fully or partially closed schools, the reduction in MVPA was considerably higher than in periods with fewer school restrictions (SCI ≥ 2: SMD, -0.57; 95% CI, -0.96 to -0.17 versus SCI < 2: SMD, -0.19; 95% CI, -1.04 to 0.67; Fig. 4). Consideration of the restriction duration prior to measurement did not reveal any trend (Restriction before measurement ≥ 30 days: SMD, -0.36; 95% CI, -0.80 to 0.08; I^2^ = 93%; Fig. S18; Restriction before measurement ≥ 60 days: SMD, -0.30; 95% CI, -0.89 to 0.29; I^2^ = 94%; Fig. S19; Restriction before measurement ≥ 90 days: SMD, -0.36; 95% CI, -1.46 to 0.74; I^2^ = 90%; Fig. S20).

### Sporting activity

Sporting activity was analyzed in three publications with different measurement instruments (self-reported score points [31, 32] and self-reported minutes/week [51]), two of them originating from the same study population with different measurement time points [31, 32]. As a result, no meta-analysis was performed. All of the studies described a statistically significant decline in sporting activity among children and adolescents both for spring 2020 [31, 51] and winter 2020/2021 [32]. Certainty of evidence when considering all three comparisons was rated as ‘low’.

### Heterogeneity, sensitivity analysis and publication bias

The meta-analyses revealed substantial heterogeneity (I^2^ > 50% and wide prediction intervals) for the most part. We conducted meta-regression analyses using a range of different variables; however, none of these variables acted as moderator (AF1: Tables S10-S13).

We also performed sensitivity analyses by comparing the following: (1) cohort studies versus cross-sectional studies versus retrospective studies (if available); (2) converted versus unconverted effect estimates (e.g. summarizing weekday and weekend measures); and (3) adjusted versus unadjusted effect estimates (AF1: Tables S14-S15). We found no significant differences, except when comparing adjusted versus unadjusted effect estimates in TPA, although only one study with adjusted values was available.

To assess publication bias, we created (contour-enhanced) funnel plots for TPA, MVPA and SA (AF1: Figs. S21-S23). Visual inspection suggests some degree of reporting bias for both outcomes. In the application of Egger's test, a reporting bias for MVPA was confirmed (*p* = 0.02; AF1: Table S16) and also indicated for TPA (*p* = 0.052; AF1: Table S16).

## Discussion

Our objective was to assess the impact of the COVID-19 pandemic and PHSM on physical activity among children and adolescents in Europe and to identify possible vulnerable subgroups. Overall, the results of this systematic review indicate a considerable decline in TPA, MVPA and SA in comparison with pre-pandemic values. Our analysis revealed that stringent school closures (partially or fully closed schools) are associated with a higher decline in TPA and MVPA versus schools with either no restrictions or only a small number of restrictions. Furthermore, the analyses emphasized a noticeable decrease in TPA and MVPA in middle childhood (8 to 12 years) and in TPA among adolescents. To our knowledge, this is the first systematic review on physical activity changes among youth from the WHO European Region considering pandemic-related restrictions and various subgroups.

Even before the pandemic, children and adolescents in Europe were not physically active enough [74]. Our study revealed that TPA in European children and adolescents declined further in a pre-during-comparison.. This corresponds to a reduction of approximately 48 min per day when considering accelerometer measurements only. MVPA also decreased in European youth, corresponding to a reduction of 12 min per day in accelerometer measurements. Moreover, SA showed a decline, even without effect pooling. Former reviews have also documented a decline in total PA during the COVID-19 pandemic ranging between 11 to 91 min per day [75, 76] respectively a reduction of 20% for TPA or 28% for MVPA [13]. Our results confirmed the general decline for European children and adolescents and further highlighted that this decline affects all types of physical activity. The decrease of 12 min per day in MVPA represents a 20% drop in what is recommended. However, we assume that there was a large variation in MVPA change as suggested by the wide prediction interval ranging even to more than 53 min decrease in MVPA regarding the lower limit. Also the reduction in TPA of 48 min per day represents a severe change in the daily routine of European youth.

Our analyses outline a possible association between stricter school closures (partial or full closure) and more significant reductions in both TPA and MVPA. This is consistent with two recent meta-analyses, which reported that during stringent PHSM and periods of school closure depression [28] and anxiety symptoms [77] among children and adolescents increased in particular. Thus, school closures seem to represent particularly sensitive periods for suboptimal health outcomes in children and adolescents. Our results must be placed in the context of the formation of research on health habits [6, 78], which proposes that (healthy) habits depend on stability mechanisms. This stability – based on family, social, and school support – was substantially disrupted for children and adolescents during strict lockdowns or school closures. From a public health perspective, it is imperative to note that perpetuation of inactive behaviors in young age contributes to tracking inactive patterns into adulthood, which in turn is associated with numerous suboptimal health consequences [7, 79]. Once restrictions had been lifted, a return to an active daily life seemed to pose a challenge for some children and adolescents [80]. A recent systematic review also points to an association between physical activity and youth’s mental health during the COVID-19 pandemic [81]. School closures also imply the elimination of physical activity in the school setting and for getting from one place to another, which contribute to the overall reduction. This highlights the importance of maintaining physical activity services and opportunities even during times of crisis, considering broader contextual and environmental conditions.

The pandemic-related reduction in physical activity varies between age groups. Our analyses revealed that children in middle childhood, aged approximately 8 to 12 years, recorded the strongest reductions in TPA and MVPA. Adolescents recorded a significant reduction in TPA. In contrast, there was no significant association for children aged 4 to 7 years, who were in pre-school or in the first year of elementary school, which is consistent with previous literature [82]. A decline in youth’s physical activity as they get older – particularly evident in early and late adolescence – was also documented even before the pandemic [7]. However, this trend in inactivity appears to have spread considerably into middle childhood (8 to 12 years of age) during the COVID-19 pandemic. This inactivity expansion in middle childhood could be a consequence of closing schools and restricting access during the COVID-19 pandemic to physical activity opportunities, which are more physically active in (un-) organized sports than younger children [83]. Further, a lack of adult activation and supervision during the COVID-19 pandemic was described as a main barrier for physical activity in middle childhood [84] and also parents’ attitudes towards risk, which have become more severe during the pandemic, correspond with children’s activity status [85]. Therefore, the group of middle childhood might represent a ‘new’ vulnerable group that should be addressed in further analyses.

Analyses based on the measurement time point revealed a significant reduction in TPA and MVPA at the beginning of the pandemic (spring/summer 2020). Taking into account an evident decline in physical activity among adolescents since 2001 [8], we can assume that the COVID-19 pandemic accelerated this process. Further closer monitoring and analyzing of physical activity among children and adolescents is essential to identify trends, specify vulnerable subgroups and ensure appropriate interventions implementation.

Stratification by gender revealed no significant differences. This result is in contrast to some primary studies [35, 42], although other reviews do not confirm a significant difference [13] and some reviews did not analyse a possible difference by gender [14, 76]. Considering all available data, significant decreases were revealed for both TPA and MVPA. When separated by measurement instrument (self-reported vs. accelerometer measurement), only self-reported TPA showed a significant decrease. Indeed, all measurement instruments were validated this could indicate an inaccuracy of the self-rating instruments as already reported in other studies [86, 87]. This emphasizes the need for stratifying results by measurement instrument in systematic reviews addressing physical activity.

The certainty of evidence assessment with the GRADE approach resulted in a low certainty for the analyzed outcomes meaning that the true effect might be markedly different from the estimated effect [88]. However, it must be noted, that GRADE was primarily developed for assessing the certainty of evidence of classical clinical questions according to the PICO-scheme (patient, intervention, comparison, outcome) and that a precise adaptation for public health questions is lacking [89]. Beyond the scope of this systematic review, the GRADE working Group suggested Evidence to Decision (EtD) criteria for making clinical recommendations, health system or public health recommendations. Although the EtD framework cannot be applied completely on our research question, important criteria from a population perspective (e.g. problem priority, desirable [un-]anticipated effects, certainty of evidence, equity, acceptability, and feasibility) allow a placement of our results [90–92]: It can be supposed that the consequences of decreasing physical activity levels during the COVID-19 pandemic among children and adolescents would be serious [1, 2]. Increasing physical activity is associated with a variety of short- and long-term health effects in children and adolescents (see explanations above). Adverse effects of increasing physical activity might be possible, but mainly in elite sports [93]. Thus, the desired effects outweigh the undesired effects. A positive cost-effectiveness rate [94, 95] and a reduction in social inequality [96, 97] can be assumed when interventions to increase physical activity are implemented. Implementation of interventions to increase physical activity is well feasible and should be based on the best available evidence [98].

It can be assumed that the opportunity costs in health terms will be high for the more than 156 million children and adolescents aged 0 to 19 years in Europe [99] due to the decline in physical activity (as outlined in our review), rise in mental health disorders [28, 77], increase in obesity [100] and screen time [101]. Additionally, financial and social constraints [102], and health impairments like immune function and viral and bacterial infections [103, 104] further affect the state of health of children and adolescents. No estimates are available on this yet, however.

Consequently, the downward spiral must be reversed. This is also underlined by the ‘strong recommendation’ of the WHO that ‘Children and adolescents should do at least an average of 60 min per day of moderate-to-vigorous-intensity […]’ [1]. Beyond the findings of this review and considering the scientific evidence, we suggest the following immediate short-term and long-term action by policy-makers and practitioners:

(I) (Re-)increase physical activity through low-threshold, comprehensive, targeted, and evidence-based interventions [1, 105]. Special attention must be given here to vulnerable groups that are either already known or are to be identified. Schools and educational settings in particular are important locations for promoting physical activity as they reach children and adolescents on a broad basis, regardless of their socio-cultural background [1]. In contrast to previous – often unsuccessful – programs in school and educational settings [7], future programs should include multi-component interventions (e.g. comprehensive school physical activity programs [106, 107]). Physical education in the school environment should communicate physical activity as a positive element in an individual’s lifestyle, and one that should be integrated as a constant component in daily life [108, 109]. For this purpose, social support from family and friends as well as access to green places are important components in the implementation and stabilization of an active lifestyle among children and adolescents [7, 105, 110–112]. Moreover, the application of digital interventions to promote physical activity (eHealth) should be strengthened in the design of programs [113, 114]. These can also be applied in periods of crisis.
(II) Implementation of a global and national monitoring and surveillance systems for the adversely impacted youth cohorts over a longer time period in order to assess medium-term and long-term health consequences and to be able to implement targeted health improvement interventions [115–118]. 
(III) ﻿Restriction in youth’s social life and the closure of educational institutions should be carefully considered, taking into account children’s rights [119] the best scientific evidence.

## Strengths and limitations

This systematic review adheres to the methodological recommendation of the Cochrane Handbook for Systematic Reviews [18]. The main strength is the broad number of studies that were able to be included, despite the restrictive inclusion criteria (only studies with a pre-pandemic baseline and instrument validation were incorporated); this improves the trustworthiness of the results. Furthermore, in an improvement over previous studies, outcomes could be separated into TPA, MVPA and SA. Authors of the studies were also contacted to provide further data, enabling to include unpublished data.

The evidence identified in this review also has several limitations. First, RoB was rated high or very high for over 38% of the studies included. Second, there was a high degree of heterogeneity for the most part in the meta-analyses and a publication bias was determined in MVPA. We addressed these by downgrading the certainty of evidence in GRADE and provided further analyses (meta-regression, sensitivity analyses). Third, the data available for young children (under 7 years) was limited. However, this age group appears to meet the TPA and MVPA recommendations [82]. Fourth, the analyses for school closures revealed a wide and overlapping subgroup CI and non-significance of the test for some subgroup analyses. The assumptions set out should therefore be interpreted with caution and further research is needed to confirm or refute these findings. Fifth, only a small number of studies from Eastern Europe were included and no appropriate pooling for single countries was possible. Sixth, subgroup analyses concerning social status were not possible due to a lack of data. Seventh, based on the literature search through to January 2023, analyses of the development of PA in the course of the pandemic and its aftermath are limited. It will take several more years to capture the longer-term trend in physical activity. Eight, the impact of the COVID-19 pandemic on the reduction in physical activity must be interpreted with caution. By performing a pre-during-comparison and stratifying by School Closure Index, we addressed this limitation and attempted to minimize it.

## Conclusions

Among children and adolescents in Europe, TPA, MVPA and SA declined sharply during the COVID-19 pandemic. This was the case in particular for TPA and MVPA among the population groups of middle childhood (8 to 12 years) and for TPA among adolescents. There are indications that reductions were most pronounced during pandemic-related school closures. Our findings suggest that the decline in physical activity during the pandemic could accelerate the long-term trend in declining physical activity among CA. Rigorous strategies and ambitious (school) programs to increase physical activity are therefore required, along with long-term monitoring of further trends.

### Supplementary Information

**Additional file 1: Table S1.** PRISMA item checklist for systematic reviews. **Table S2.** Deviations from the systematic review protocol. **Table S3.** Searched websites of key organizations. **Table S4.** Search strategy. **Table S5.** Reasons for exclusion of studies from the systematic literature search, after full-text screening. **Table S6.** Data conversion. **Table S7.** Criteria for grading evidence according to Grading of Recommendations, Assessment, Development and Evaluations (GRADE). **Table S8.** Evidence profile for grading evidence according to Grading of Recommendations, Assessment, Development and Evaluations (GRADE). **Table S9.** Summary of effect estimates. **Table S10.** Meta-regression for total physical activity with categorical moderators. **Table S11.** Meta-regression for total physical activity with continuous moderators. **Table S12.** Meta-regression for moderate-to-vigorous physical activity with categorical moderators. **Table S13.** Meta-regression for moderate-to-vigorous physical activity with continuous moderators. **Table S14.** Sensitivity analysis for total physical activity. **Table S15.** Sensitivity analysis for moderate-to-vigorous physical activity. **Table S16.** Eggers’ test. **Figure S1.** PRISMA Flow Chart. **Figure S2.** Graphical distribution of the studies included. **Figure S3.** Traffic-light plots of the domain-level judgements for each individual result. **Figure S4.** Weighted-bar plots of the distribution of risk of bias judgements within each bias domain. **Figure S5.** Forest plot of changes in total physical activity comparing before and during COVID-19 pandemic, using Physical Activity Questionnaire for Children and Adolescents. **Figure S6.** Forest plot of changes in total physical activity comparing before and during COVID-19 pandemic, using accelerometer measurements. **Figure S7.** Forest plot of changes in female and male total physical activity comparing before and during COVID-19 pandemic. **Figure S8.** Forest plot of changes according to time course in total physical activity comparing before and during COVID-19 pandemic. **Figure S9.** Forest plot of changes according to a restriction length > 30 days before measurement in total physical activity comparing before and during COVID-19 pandemic. **Figure S10.** Forest plot of changes according to a restriction length > 60 days before measurement in total physical activity comparing before and during COVID-19 pandemic. **Figure S11.** Forest plot of changes according to a restriction length > 90 days before measurement in total physical activity comparing before and during COVID-19 pandemic. **Figure S12.** Forest plot of changes in moderate-to-vigorous physical activity comparing before and during COVID-19 pandemic. **Figure S13.** Forest plot of changes in moderate-to-vigorous physical activity comparing before and during COVID-19 pandemic, using self-reported score measurements. **Figure S14.** Forest plot of changes in moderate-to-vigorous physical activity comparing before and during COVID-19 pandemic, using accelerometer measurements. **Figure S15.** Forest plot of changes in female and male moderate-to-vigorous physical activity comparing before and during COVID-19 pandemic. **Figure S16.** Forest plot of changes in moderate-to-vigorous physical activity comparing different age groups. **Figure S17.** Forest plot of changes according to time course in moderate-to-vigorous physical activity comparing before and during COVID-19 pandemic. **Figure S18.** Forest plot of changes according to a restriction length > 30 days before measurement in moderate-to-vigorous physical activity comparing before and during COVID-19 pandemic. **Figure S19.** Forest plot of changes according to a restriction length > 60 days before measurement in moderate-to-vigorous physical activity comparing before and during COVID-19 pandemic. **Figure S20.** Forest plot of changes according to a restriction length > 90 days before measurement in moderate-to-vigorous physical activity comparing before and during COVID-19 pandemic. **Figure S21.** Funnel plot of changes in total physical activity comparing before and during COVID-19 pandemic. **Figure S22.** Funnel plot of changes in moderate-to-vigorous physical activity comparing before and during COVID-19 pandemic. **Figure S23.** Funnel plot of changes in sporting activity comparing before and during COVID-19 pandemic**.**

## Data Availability

All data are included in the manuscript and additional file.

## Abbreviations

AF: Additional file
CI: Confidence interval
COVID-19: Coronavirus disease 2019
EtD: Evidence to Decision
GRADE: Grading of Recommendations Assessment, Development and Evaluation
MVPA: Moderate-to-vigorous physical activity
PHSM: Public health and social measures
PRISMA: Preferred Reporting Items for Systematic Reviews and Meta-analyses
PRESS: Peer Review of Electronic Search Strategies
PROSPERO: International Prospective Register of Systematic Reviews
RoB: Risk of bias
ROBINS-E: Risk of Bias in Non-randomized Studies - of Exposure
TPA: Total physical activity
SA: Sporting activity
SD: Standard deviation
SMD: Standardized mean differences
