# Supplementary material for: How the COVID-19 pandemic and related school closures reduce physical activity among children and adolescents in the WHO European Region: a systematic review and meta-analysis
Source: Int J Behav Nutr Phys Act. 2023 Dec 19;20:149. doi: 10.1186/s12966-023-01542-x (PMC10731871; doi:10.1186/s12966-023-01542-x)
Supplement: Supplementary file 1 — Additional file 1: Table S1. PRISMA item checklist for systematic reviews. Table S2. Deviations from the systematic review protocol. Table S3. Searched websites of key organizations. Table S4. Search strategy. Table S5. Reasons for exclusion of studies from the systematic literature search, after full-text screening. Table S6. Data conversion. Table S7. Criteria for grading evidence according to Grading of Recommendations, Assessment, Development and Evaluations (GRADE). Table S8. Evidence profile for grading evidence according to Grading of Recommendations, Assessment, Development and Evaluations (GRADE). Table S9. Summary of effect estimates. Table S10. Meta-regression for total physical activity with categorical moderators. Table S11. Meta-regression for total physical activity with continuous moderators. Table S12. Meta-regression for moderate-to-vigorous physical activity with categorical moderators. Table S13. Meta-regression for moderate-to-vigorous physical activity with continuous moderators. Table S14. Sensitivity analysis for total physical activity. Table S15. Sensitivity analysis for moderate-to-vigorous physical activity. Table S16. Eggers’ test. Figure S1. PRISMA Flow Chart. Figure S2. Graphical distribution of the studies included. Figure S3. Traffic-light plots of the domain-level judgements for each individual result. Figure S4. Weighted-bar plots of the distribution of risk of bias judgements within each bias domain. Figure S5. Forest plot of changes in total physical activity comparing before and during COVID-19 pandemic, using Physical Activity Questionnaire for Children and Adolescents. Figure S6. Forest plot of changes in total physical activity comparing before and during COVID-19 pandemic, using accelerometer measurements. Figure S7. Forest plot of changes in female and male total physical activity comparing before and during COVID-19 pandemic. Figure S8. Forest plot of changes according to time course in total physical activity comparing be [file 12966_2023_1542_MOESM1_ESM.zip › Supplement_PhysAct_ChildAdol_COV19.docx]

**Supplementary information**

**How COVID-19 pandemic and related school closures reduce physical activity among children and adolescents in the WHO European Region: A systematic review and meta-analysis**

Helena Ludwig-Walz ^a^; Waldemar SIEMENS ^b,c^; Sarah HEINISCH ^d^; Indra DANNHEIM ^e,f^; Julika LOSS ^g^; Martin BUJARD ^h,i^

^a^ German Institute for Population Research (BiB), Wiesbaden, Germany (HLW),
helena.ludwig-walz@bib.bund.de, ORCID: 0000-0003-1558-8241

^b^ Institute for Evidence in Medicine, Medical Center - University of Freiburg, Faculty of Medicine, Freiburg, Germany (WS), waldemar.siemens@uniklinik-freiburg.de, ORCID: 0000-0003-4238-5327

^c^ Cochrane Germany, Cochrane Germany Foundation, Freiburg, Germany (WS), waldemar.siemens@uniklinik-freiburg.de, ORCID: 0000-0003-4238-5327

^d^ University of Education, Karlsruhe, Germany (SH), sarah.heinisch@ph-karlsruhe.de

^e^ Regional Innovative Centre of Health and Quality of Live Fulda (RIGL), Fulda University of Applied Sciences, Fulda, Germany (ID), indra.dannheim@oe.hs-fulda.de, ORCID: 0000-0002-4478-7808

^f^ Department of Nutritional, Food and Consumer Sciences, Fulda University of Applied Sciences, Fulda, Germany (ID), indra.dannheim@oe.hs-fulda.de, ORCID: 0000-0002-4478-7808

^g^ Department of Epidemiology and Health Monitoring, Robert Koch Institute, Berlin, Germany (JL), [lossj@rki.de](mailto:lossj@rki.de)

^h^ German Institute for Population Research (BiB), Wiesbaden, Germany (MB), martin.bujard@bib.bund.de, ORCID: 0000-0002-3603-4160

^i^ Institute for Medical Psychology, Medical Faculty, University Heidelberg, Heidelberg, Germany (MB), martin.bujard@bib.bund.de, ORCID: 0000-0002-3603-4160

Legend

[Table S1: PRISMA item checklist for systematic reviews 4](#_Toc144810694)

[Table S2: Deviations from the systematic review protocol 7](#_Toc144810695)

[Table S3: Searched websites of key organizations 7](#_Toc144810696)

[Table S4: Search strategy 8](#_Toc144810697)

[Table S5: Reasons for exclusion of studies from the systematic literature search, after full-text screening 16](#_Toc144810698)

[Table S6: Data conversion 20](#_Toc144810699)

[Table S7: Criteria for grading evidence according to Grading of Recommendations, Assessment, Development and Evaluations (GRADE) 21](#_Toc144810700)

[Table S8: Evidence profile for grading evidence according to Grading of Recommendations, Assessment, Development and Evaluations (GRADE) 24](#_Toc144810701)

[Table S9: Summary of effect estimates 26](#_Toc144810702)

[Table S10: Meta-regression for total physical activity with categorical moderators 35](#_Toc144810703)

[Table S11: Meta-regression for total physical activity with continuous moderators 35](#_Toc144810704)

[Table S12: Meta-regression for moderate-to-vigorous physical activity with categorical moderators 35](#_Toc144810705)

[Table S13: Meta-regression for moderate-to-vigorous physical activity with continuous moderators 36](#_Toc144810706)

[Table S14: Sensitivity analysis for total physical activity 36](#_Toc144810707)

[Table S15: Sensitivity analysis for moderate-to-vigorous physical activity 36](#_Toc144810708)

[Table S16: Eggers’ test 36](#_Toc144810709)

[Figure S1: PRISMA Flow Chart 37](#_Toc144810710)

[Figure S2: Graphical distribution of the studies included 38](#_Toc144810711)

[Figure S3: Traffic-light plots of the domain-level judgements for each individual result 39](#_Toc144810712)

[Figure S4: Weighted-bar plots of the distribution of risk of bias judgements within each bias domain 40](#_Toc144810713)

[Figure S5: Forest plot of changes in total physical activity comparing before and during COVID-19 pandemic, using Physical Activity Questionnaire for Children and Adolescents 40](#_Toc144810714)

[Figure S6: Forest plot of changes in total physical activity comparing before and during COVID-19 pandemic, using accelerometer measurements 40](#_Toc144810715)

[Figure S7: Forest plot of changes in female and male total physical activity comparing before and during COVID-19 pandemic 40](#_Toc144810716)

[Figure S8: Forest plot of changes according to time course in total physical activity comparing before and during COVID-19 pandemic 40](#_Toc144810717)

[Figure S9: Forest plot of changes according to a restriction length >30 days before measurement in total physical activity comparing before and during COVID-19 pandemic 40](#_Toc144810718)

[Figure S10: Forest plot of changes according to a restriction length >60 days before measurement in total physical activity comparing before and during COVID-19 pandemic 40](#_Toc144810719)

[Figure S11: Forest plot of changes according to a restriction length >90 days before measurement in total physical activity comparing before and during COVID-19 pandemic 40](#_Toc144810720)

[Figure S12: Forest plot of changes in moderate-to-vigorous physical activity comparing before and during COVID-19 pandemic 40](#_Toc144810721)

[Figure S13: Forest plot of changes in moderate-to-vigorous physical activity comparing before and during COVID-19 pandemic, using self-reported score measurements 40](#_Toc144810722)

[Figure S14: Forest plot of changes in moderate-to-vigorous physical activity comparing before and during COVID-19 pandemic, using accelerometer measurements 40](#_Toc144810723)

[Figure S15: Forest plot of changes in female and male moderate-to-vigorous physical activity comparing before and during COVID-19 pandemic 40](#_Toc144810724)

[Figure S16: Forest plot of changes in moderate-to-vigorous physical activity comparing different age groups 40](#_Toc144810725)

[Figure S17: Forest plot of changes according to time course in moderate-to-vigorous physical activity comparing before and during COVID-19 pandemic 41](#_Toc144810726)

[Figure S18: Forest plot of changes according to a restriction length >30 days before measurement in moderate-to-vigorous physical activity comparing before and during COVID-19 pandemic 41](#_Toc144810727)

[Figure S19: Forest plot of changes according to a restriction length >60 days before measurement in moderate-to-vigorous physical activity comparing before and during COVID-19 pandemic 41](#_Toc144810728)

[Figure S20: Forest plot of changes according to a restriction length >90 days before measurement in moderate-to-vigorous physical activity comparing before and during COVID-19 pandemic 41](#_Toc144810729)

[Figure S21: Funnel plot of changes in total physical activity comparing before and during COVID-19 pandemic 41](#_Toc144810730)

[Figure S22: Funnel plot of changes in moderate-to-vigorous physical activity comparing before and during COVID-19 pandemic 41](#_Toc144810731)

[Figure S23: Funnel plot of changes in sporting activity comparing before and during COVID-19 pandemic 41](#_Toc144810732)

[References 42](#_Toc144810733)

# Table S1: PRISMA item checklist for systematic reviews

| **Topic** | **No.** | **Item** | **Location where item is reported** |
| --- | --- | --- | --- |
| **TITLE** |  |  |  |
| **Title** | 1 | Identify the report as a systematic review. | Title |
| **ABSTRACT** |  |  |  |
| **Abstract** | 2 | See the PRISMA 2020 for Abstracts checklist | not applicable |
| **INTRODUCTION** |  |  |  |
| **Rationale** | 3 | Describe the rationale for the review in the context of existing knowledge. | Background |
| **Objectives** | 4 | Provide an explicit statement of the objective(s) or question(s) the review addresses. | Background |
| **METHODS** |  |  |  |
| **Eligibility criteria** | 5 | Specify the inclusion and exclusion criteria for the review and how studies were grouped for the syntheses. | Methods: Eligibility criteria |
| **Information sources** | 6 | Specify all databases, registers, websites, organisations, reference lists and other sources searched or consulted to identify studies. Specify the date when each source was last searched or consulted. | Methods: Information sources and search strategy |
| **Search strategy** | 7 | Present the full search strategies for all databases, registers and websites, including any filters and limits used. | Table S4 |
| **Selection process** | 8 | Specify the methods used to decide whether a study met the inclusion criteria of the review, including how many reviewers screened each record and each report retrieved, whether they worked independently, and if applicable, details of automation tools used in the process. | Methods: Information sources and search strategy |
| **Data collection process** | 9 | Specify the methods used to collect data from reports, including how many reviewers collected data from each report, whether they worked independently, any processes for obtaining or confirming data from study investigators, and if applicable, details of automation tools used in the process. | Methods: Selection process |
| **Data items** | 10a | List and define all outcomes for which data were sought. Specify whether all results that were compatible with each outcome domain in each study were sought (e.g. for all measures, time points, analyses), and if not, the methods used to decide which results to collect. | Methods: Data extraction |
|  | 10b | List and define all other variables for which data were sought (e.g. participant and intervention characteristics, funding sources). Describe any assumptions made about any missing or unclear information. | Methods: Data extraction |
| **Study risk of bias assessment** | 11 | Specify the methods used to assess risk of bias in the included studies, including details of the tool(s) used, how many reviewers assessed each study and whether they worked independently, and if applicable, details of automation tools used in the process. | Methods: Risk of bias assessment |
| **Effect measures** | 12 | Specify for each outcome the effect measure(s) (e.g. risk ratio, mean difference) used in the synthesis or presentation of results. | Methods: Data extraction |
| **Synthesis methods** | 13a | Describe the processes used to decide which studies were eligible for each synthesis (e.g. tabulating the study intervention characteristics and comparing against the planned groups for each synthesis (item 5)). | Methods: Synthesis methods |
|  | 13b | Describe any methods required to prepare the data for presentation or synthesis, such as handling of missing summary statistics, or data conversions. | Methods: Synthesis methods |
|  | 13c | Describe any methods used to tabulate or visually display results of individual studies and syntheses. | Methods: Synthesis methods |
|  | 13d | Describe any methods used to synthesize results and provide a rationale for the choice(s). If meta-analysis was performed, describe the model(s), method(s) to identify the presence and extent of statistical heterogeneity, and software package(s) used. | Methods: Synthesis methods |
|  | 13e | Describe any methods used to explore possible causes of heterogeneity among study results (e.g. subgroup analysis, meta-regression). | Methods: Synthesis methods |
|  | 13f | Describe any sensitivity analyses conducted to assess robustness of the synthesized results. | Methods: Synthesis methods |
| **Reporting bias assessment** | 14 | Describe any methods used to assess risk of bias due to missing results in a synthesis (arising from reporting biases). | Methods: Synthesis methods |
| **Certainty assessment** | 15 | Describe any methods used to assess certainty (or confidence) in the body of evidence for an outcome. | Methods: Certainty of evidence assessment |
| **RESULTS** |  |  |  |
| **Study selection** | 16a | Describe the results of the search and selection process, from the number of records identified in the search to the number of studies included in the review, ideally using a flow diagram. | Results, Figure S1 |
|  | 16b | Cite studies that might appear to meet the inclusion criteria, but which were excluded, and explain why they were excluded. | Table S5 |
| **Study characteristics** | 17 | Cite each included study and present its characteristics. | Table 1 & Table S9 |
| **Risk of bias in studies** | 18 | Present assessments of risk of bias for each included study. | Figures S3 and S4 |
| **Results of individual studies** | 19 | For all outcomes, present, for each study: (a) summary statistics for each group (where appropriate) and (b) an effect estimate and its precision (e.g. confidence/credible interval), ideally using structured tables or plots. | Table S9 |
| **Results of syntheses** | 20a | For each synthesis, briefly summarise the characteristics and risk of bias among contributing studies. | Results, Table 1 |
|  | 20b | Present results of all statistical syntheses conducted. If meta-analysis was done, present for each the summary estimate and its precision (e.g. confidence/credible interval) and measures of statistical heterogeneity. If comparing groups, describe the direction of the effect. | Results,  Figures 1-4, Figures S5-20 |
|  | 20c | Present results of all investigations of possible causes of heterogeneity among study results. | Results, Table S9-S12 |
|  | 20d | Present results of all sensitivity analyses conducted to assess the robustness of the synthesized results. | Results,  Table S13 and S14 |
| **Reporting biases** | 21 | Present assessments of risk of bias due to missing results (arising from reporting biases) for each synthesis assessed. | Results,  Table S15, Figures S21-S23 |
| **Certainty of evidence** | 22 | Present assessments of certainty (or confidence) in the body of evidence for each outcome assessed. | Results, Tables S7 and S8 |
| **DISCUSSION** |  |  |  |
| **Discussion** | 23a | Provide a general interpretation of the results in the context of other evidence. | Discussion |
|  | 23b | Discuss any limitations of the evidence included in the review. | Discussion: Strengths and limitations |
|  | 23c | Discuss any limitations of the review processes used. | Discussion: Strengths and limitations |
|  | 23d | Discuss implications of the results for practice, policy, and future research. | Discussion |
| **OTHER INFORMATION** |  |  |  |
| **Registration and protocol** | 24a | Provide registration information for the review, including register name and registration number, or state that the review was not registered. | Methods |
|  | 24b | Indicate where the review protocol can be accessed, or state that a protocol was not prepared. | Methods |
|  | 24c | Describe and explain any amendments to information provided at registration or in the protocol. | Methods |
| **Support** | 25 | Describe sources of financial or non-financial support for the review, and the role of the funders or sponsors in the review. | not applicable |
| **Competing interests** | 26 | Declare any competing interests of review authors. | Competing interests |
| **Availability of data, code and other materials** | 27 | Report which of the following are publicly available and where they can be found: template data collection forms; data extracted from included studies; data used for all analyses; analytic code; any other materials used in the review. | Supplementary information (online) |

# Table S2: Deviations from the systematic review protocol

| **Deviations** | - Due to the high number of relevant findings for both physical activity and physical fitness, which allow for in depth differentiation between subgroups, time and three different outcome variables, the author team decided to report the results for the outcomes in two publications. Therefore, the analyses for physical activity are presented in this publication. - Sporting activity was added as primary outcome. - Age categories were defined on the basis of Centers for Disease Control and Prevention [1]. - No subgroup analyses regarding social status were possible. |
| --- | --- |

# Table S3: Searched websites of key organizations

| **Websites of key organizations:** | - Centers for Disease Control and Prevention (CDC) - Council of Europe - European Centre for Disease Prevention and Control - European Union - National Health Service - Save the Children - Sport England - United Nations International Children's Emergency Fund (UNICEF) - World Health Organization (WHO), Regional Office for Europe - Organization for Economic Co-operation and Development (OECD) |
| --- | --- |

# Table S4: Search strategy

**PubMed**

| **Population** | Infan* OR toddler* OR minors OR minors* OR boy OR boys OR boyfriend OR boyhood OR girl* OR kid OR kids OR child OR child* OR children* OR schoolchild* OR schoolchild OR school child[tiab] OR school child*[tiab] OR adolescen* OR juvenil* OR youth* OR teen* OR under*age* OR pubescen* OR pediatrics[mh] OR pediatric* OR paediatric* OR peadiatric* OR school*[tiab] OR kindergarten*[tiab] OR kindergarden*[tiab] |
| --- | --- |
| **Intervention** | ("Covid-19"[Mesh] OR covid*[tiab] OR "SARS-CoV-2"[Mesh] OR “sars-2”[tiab] OR “sars2”[tiab] OR “sars 2”[tiab] OR “sars-cov-19”[tiab] OR “sars-cov19”[tiab] OR “sarscov-19”[tiab] OR “sarscov19”[tiab] OR “sars cov 19”[tiab] OR “sarscov 19”[tiab] OR “sars cov19”[tiab] OR "sars-cov-2"[tiab] OR “sarscov-2”[tiab] OR “sars-cov2”[tiab] OR “sarscov2”[tiab] OR "sars cov 2"[tiab] OR "sarscov 2"[tiab] OR "sars cov2"[tiab] OR "Severe Acute Respiratory Syndrome" [tiab] OR "Severe Acute Respiratory disease"[tiab] OR "coronavirus"[MeSH] OR Coronavirus*[tiab] OR corona-virus*[tiab] OR “corona virus*”[tiab] OR ncov*[tiab] OR n-cov*[tiab] OR “n cov”[tiab] OR novelcov*[tiab] OR novel-cov*[tiab] OR “novel cov*”[tiab]) AND (2019/11/01[PDAT] : 3000/12/31[PDAT]) |
| **Comparison** | Pre-pandemic baseline included in the study *(will be screened manually)* |
| **Outcome** | "Exercise"[Mesh] OR "Physical Fitness"[Mesh] OR "Sports"[Mesh] OR "Dancing"[Mesh] OR "Motor Activity"[Mesh:NoExp] OR "Physical Exertion"[Mesh] OR "Exercise Movement Techniques"[Mesh] OR Human Activities[Mesh] OR ((physical[tiab]) AND (activ*[tiab] OR lifestyle[tiab] OR life-style[tiab] OR "life style"[tiab])) OR exercise*[tiab] OR fitness[tiab] OR sport*[tiab] OR "physical exertion*" OR "motor activ*"[tiab] OR "train*"[tiab] OR "physical effort*"[tiab] OR "physical endurance"[tiab] OR "physical condition*"[tiab] OR gymnastic*[tiab] OR run[tiab] OR runn*[tiab] OR swim*[tiab] OR walk*[tiab] OR jogg*[tiab] OR athletic*[tiab] OR cycl*[tiab] OR bicycl*[tiab] OR aerobic*[tiab] OR move[tiab] |
| **Species** | Humans *(screened manually)* |
| **Language** | No limit |

**Embase**

| **Population** | Infan* OR toddler* OR minors* OR boy OR boys OR boyfriend OR boyhood OR girl* OR kid OR kids OR child* OR schoolchild* OR (‘school child’ OR ‘school child*’):ab,ti OR adolescen* OR juvenil* OR youth* OR teen* OR under*age* OR pubescen* OR 'pediatrics'/exp OR pediatric* OR paediatric* OR (‘peadiatric*’ OR ‘school’ OR ‘school*’):ab,ti OR (kindergarten* OR kindergarden*):ab,ti |
| --- | --- |
| **Intervention** | (covid*:ab,ti OR 'sars 2':ab,ti OR sars2:ab,ti OR 'sars-2':ab,ti OR 'sars-cov-19':ab,ti OR 'sars-cov19':ab,ti OR 'sarscov-19':ab,ti OR 'sarscov19':ab,ti OR 'sars cov 19':ab,ti OR 'sarscov 19':ab,ti OR 'sars cov19':ab,ti OR 'sars-cov-2':ab,ti OR 'sarscov-2':ab,ti OR 'sars-cov2':ab,ti OR 'sarscov2':ab,ti OR 'sars cov 2':ab,ti OR 'sarscov 2':ab,ti OR 'sars cov2':ab,ti OR 'severe acute respiratory syndrome':ab,ti OR 'severe acute respiratory disease':ab,ti OR coronavirus*:ab,ti OR 'corona virus*':ab,ti OR 'corona-virus*':ab,ti OR ncov*:ab,ti OR 'n-cov*':ab,ti OR 'n cov':ab,ti OR novelcov*:ab,ti OR 'novel cov*':ab,ti OR 'novel-cov*':ab,ti OR 'severe acute respiratory syndrome coronavirus 2'/exp OR 'coronavirinae'/exp OR 'coronavirus'/exp) AND [01-11-2019]/sd NOT [31-12-3000]/sd |
| **Comparison** | Pre-pandemic baseline included in the study *(will be screened manually)* |
| **Outcome** | 'exercise'/exp OR 'physical activity'/exp OR 'sport'/exp OR 'dancing'/exp OR 'motor activity'/exp OR (physical:ab,ti AND (activ*:ab,ti OR lifestyle:ab,ti OR 'life style':ab,ti)) OR exercise*:ab,ti OR fitness:ab,ti OR sport*:ab,ti OR 'physical exertion*' OR 'motor activ*':ab,ti OR 'train*':ab,ti OR 'physical effort*':ab,ti OR 'physical endurance':ab,ti OR 'physical condition*':ab,ti OR gymnastic*:ab,ti OR run:ab,ti OR runn*:ab,ti OR swim*:ab,ti OR walk*:ab,ti OR jogg*:ab,ti OR athletic*:ab,ti OR cycl*:ab,ti OR aerobic*:ab,ti OR move:ab,ti OR bicycl*:ab,ti |
| **Species** | Humans *(screened manually)* |
| **Language** | No limit |

**APA PsycInfo (via EBSCOhost)**

| **Population** | (TI (Infan* OR toddler* OR minors OR minors* OR boy OR boys OR boyfriend OR boyhood OR girl* OR kid OR kids OR child OR child* OR children* OR schoolchild* OR schoolchild OR ‘school child’ OR ‘school child*’ OR adolescen* OR juvenil* OR youth* OR teen* OR underage* OR pubescen* OR pediatrics OR pediatric* OR paediatric* OR ‘school*’ OR kindergarten* OR kindergarden*)) OR (AB (Infan* OR toddler* OR minors OR minors* OR boy OR boys OR boyfriend OR boyhood OR girl* OR kid OR kids OR child OR child* OR children* OR schoolchild* OR schoolchild OR ‘school child’ OR ‘school child*’ OR adolescen* OR juvenil* OR youth* OR teen* OR underage* OR pubescen* OR pediatrics OR pediatric* OR paediatric* OR ‘school*’ OR kindergarten* OR kindergarden*)) |
| --- | --- |
| **Intervention** | (DE "COVID-19" OR DE "Coronavirus") OR (TI covid* OR AB covid*) OR (TI covid* OR AB covid*) OR (TI "SARS-CoV-2" OR AB "SARS-CoV-2") OR (TI "sars-2*" OR AB "sars-2*") OR (TI "sars2*" OR AB "sars2*") OR (TI "sars 2" OR AB "sars 2") OR (TI “sars-cov-19” OR AB “sars-cov-19”) OR (TI “sars-cov19” OR AB “sars-cov19”) OR (TI “sarscov-19” OR AB “sarscov-19”) OR (TI “sarscov19” OR AB “sarscov19”) OR (TI “sars cov 19” OR AB “sars cov 19”) OR (TI “sarscov 19” OR AB “sarscov 19”) OR (TI “sars cov19” OR AB “sars cov19”) OR (TI “sarscov-2” OR AB “sarscov-2”) OR (TI “sars-cov-2” OR AB “sars-cov-2”) OR (TI “sarscov2” OR AB “sarscov2”) OR (TI “sars-cov2” OR AB “sars-cov2”) OR (TI “sarscov2” OR AB “sarscov2”) OR (TI “sars cov 2” OR AB “sars cov 2”) OR (TI “sarscov 2” OR AB “sarscov 2”) OR (TI “sars cov2” OR AB “sars cov2”) (TI “Severe Acute Respiratory Syndrome” OR AB “Severe Acute Respiratory Syndrome”) OR (TI “Severe Acute Respiratory disease” OR AB “Severe Acute Respiratory disease”) OR (TI “Coronavirus*” OR AB “Coronavirus*”) OR (TI “Corona-virus*” OR AB “Corona-virus*”) OR (TI “Corona virus*” OR AB “Corona virus*”) OR (TI “ncov*” OR AB “ncov*”) OR (TI “n-cov*” OR AB “n-cov*”) OR (TI “n cov*” OR AB “n cov*”) OR (TI “novelcov*” OR AB “novelcov*”) OR (TI “novel-cov*” OR AB “novel-cov*”) OR (TI “novel cov*” OR AB “novel cov*”) |
| **Comparison** | Pre-pandemic baseline included in the study *(will be screened manually)* |
| **Outcome** |  |
|  | Humans *(screened manually)* |
| **Limits** | No limit |

**Cochrane Library**

| **Population** | #1 MeSH descriptor: [Infant] explode all trees  #2 MeSH descriptor: [Child] explode all trees  #3 MeSH descriptor: [Adolescent] explode all trees  #4 MeSH descriptor: [Pediatrics] explode all trees  #5 MeSH descriptor: [Schools] explode all trees  #6 (Infan* OR toddler* OR minors OR minors* OR boy OR boys OR boyfriend OR boyhood OR girl* OR kid OR kids OR child* OR schoolchild* OR schoolchild OR "school NEXT child*" OR adolescen* OR juvenil* OR youth* OR teen* OR under-age* OR pubescen* OR paediatric* OR peadiatric* OR school* OR kindergarten* OR kindergarden*):ti,kw,ab  #7 #1 OR #2 OR #3 OR #4 OR #5 OR #6 |
| --- | --- |
| **Intervention** | #8 MeSH descriptor: [COVID-19] explode all trees  #9 MeSH descriptor: [Coronavirus] explode all trees  #10 MeSH descriptor: [undefined] explode all trees  #11 (covid* OR sars-2 OR sars2 OR "sars NEXT 2" OR sars-cov-19 OR sars-cov19 OR sarscov-19 OR “sars NEXT cov NEXT 19” OR “sarscov NEXT 19” OR “sars NEXT cov19” OR sarscov19 OR SARS-CoV-2 OR sarscov-2 OR sars-cov2 OR sarscov2 OR "sars NEXT cov NEXT 2" OR "sarscov NEXT 2" OR "sars NEXT cov2" OR "Severe NEXT Acute NEXT Respiratory NEXT Syndrome" OR "Severe NEXT Acute NEXT Respiratory NEXT disease" OR Coronavirus* OR corona-virus* OR "corona NEXT virus*" OR ncov* OR n-cov* OR "n NEXT cov" OR novelcov* OR novel-cov* OR "novel NEXT cov*"):ti,ab,kw (Word variations have been searched)  #12 #8 OR #9 OR #10 OR #11 |
| **Comparison** | Pre-pandemic baseline included in the study *(will be screened manually)* |
| **Outcome** | #13 MeSH descriptor: [Exercise] explode all trees  #14 MeSH descriptor: [Sports] explode all trees  #15 MeSH descriptor: [Dancing] explode all trees  #16 MeSH descriptor: [Motor Activity] explode all trees  #17 MeSH descriptor: [Physical Exertion] explode all trees  #18 MeSH descriptor: [Exercise Movement Techniques] explode all trees  #19 MeSH descriptor: [Human Activities] explode all trees  #20 (((physical) AND (activ* OR lifestyle OR life-style OR "life style")) OR exercise* OR fitness OR sport* OR "physical exertion*" OR "motor activ*" OR "train*" OR "physical effort*" OR "physical endurance" OR "physical condition*" OR gymnastic* OR run OR runn* OR swim* OR walk* OR jogg* OR athletic* OR cycl* OR aerobic* OR move OR bicycl*):ti,ab,kw (Word variations have been searched)  #21 #13 OR #14 OR #15 OR #16 OR #17 OR #18 OR #19 OR #20  #22 #7 AND #12 AND #21 |
|  | Humans *(screened manually)* |
| **Limits** | No limit |

**Web of Science Core Collection**

| **Population** | Infan* OR toddler* OR minors* OR boy OR boys OR boyfriend OR boyhood OR girl* OR kid OR kids OR child* OR children* OR schoolchild* OR schoolchild OR "school child" OR "school child" OR adolescen* OR juvenil* OR youth* OR teen* OR under*age* OR pubescen* OR kindergarten* OR kindergarden* (Topic) |
| --- | --- |
| **Intervention** | covid* OR "sars 2" OR sarsa OR sars-2 OR "sars cov 19" OR "sars cov19" OR "sarscov 19" OR sarscov OR "sars-cov-19" OR "sars-cov19" OR "sarscov-19" OR "sars-cov-2" OR "sarscov 2" OR "sars cov2" OR sarscov OR "sars cov 2" OR "sarscov-2" OR "sars-cov2" OR "severe acute respiratory syndrome" OR "severe acute respiratory disease" OR coronavirus* OR "corona virus*" OR "corona-virus*" OR ncov* OR "n-cov*" OR "n cov" OR novelcov* OR "novel cov*" OR "novel-cov*" (Topic) |
| **Comparison** | Pre-pandemic baseline included in the study *(will be screened manually)* |
| **Outcome** | ((physical) AND (activ* OR lifestyle OR life-style OR "life style")) OR exercise* OR fitness OR sport* OR "physical exertion*" OR "motor activ*" OR "train*" OR "physical effort*" OR "physical endurance" OR "physical condition*" OR gymnastic* OR run OR runn* OR swim* OR walk* OR jogg* OR athletic* OR cycl* OR aerobic* OR move OR bicycl* (Topic) |
| **Species** | Humans *(screened manually)* |
| **Language** | No limit |

**WHO COVID-19 database**

| **Population** | (Infan* toddler* OR minors* OR boy OR boys OR boyfriend OR boyhood OR girl* OR kid OR kids OR child* OR children* OR schoolchild* OR schoolchild OR "school child" OR "school child" OR adolescen* OR juvenil* OR youth* OR teen* OR under*age* OR pubescen* OR kindergarten* OR kindergarden*) |
| --- | --- |
| **Intervention** | (covid* OR "sars 2" OR sars2 OR sars-2 OR "sars cov 19" OR "sars cov19" OR "sarscov 19" OR sarscov19 OR "sars-cov-19" OR "sars-cov19" OR "sarscov-19" OR "sars-cov-2" OR "sarscov 2" OR "sars cov2" OR sarscov2 OR "sars cov 2" OR "sarscov-2" OR "sars-cov2" OR "severe acute respiratory syndrome" OR "severe acute respiratory disease" OR coronavirus* OR "corona virus*" OR "corona-virus*" OR ncov* OR "n-cov*" OR "n cov" OR novelcov* OR "novel cov*" OR "novel-cov*") |
| **Comparison** | Pre-pandemic baseline included in the study *(will be screened manually)* |
| **Outcome** | ((physical) AND (activ* OR lifestyle OR life-style OR "life style")) OR exercise* OR fitness OR sport* OR "physical exertion*" OR "motor activ*" OR "train*" OR "physical effort*" OR "physical endurance" OR "physical condition*" OR gymnastic* OR run OR runn* OR swim* OR walk* OR jogg* OR athletic* OR cycl* OR aerobic* OR move OR bicycl* |
| **Species** | Humans *(screened manually)* |
| **Language** | No limit |

**SPORTDiscus**

| **Population** | (Infan* toddler* OR minors* OR boy OR boys OR boyfriend OR boyhood OR girl* OR kid OR kids OR child* OR children* OR schoolchild* OR schoolchild OR "school child" OR "school child" OR adolescen* OR juvenil* OR youth* OR teen* OR under*age* OR pubescen* OR kindergarten* OR kindergarden*) |
| --- | --- |
| **Intervention** | (covid* OR "sars 2" OR sars2 OR sars-2 OR "sars cov 19" OR "sars cov19" OR "sarscov 19" OR sarscov19 OR "sars-cov-19" OR "sars-cov19" OR "sarscov-19" OR "sars-cov-2" OR "sarscov 2" OR "sars cov2" OR sarscov2 OR "sars cov 2" OR "sarscov-2" OR "sars-cov2" OR "severe acute respiratory syndrome" OR "severe acute respiratory disease" OR coronavirus* OR "corona virus*" OR "corona-virus*" OR ncov* OR "n-cov*" OR "n cov" OR novelcov* OR "novel cov*" OR "novel-cov*") |
| **Comparison** | Pre-pandemic baseline included in the study *(will be screened manually)* |
| **Outcome** | ((physical) AND (activ* OR lifestyle OR life-style OR "life style")) OR exercise* OR fitness OR sport* OR "physical exertion*" OR "motor activ*" OR "train*" OR "physical effort*" OR "physical endurance" OR "physical condition*" OR gymnastic* OR run OR runn* OR swim* OR walk* OR jogg* OR athletic* OR cycl* OR aerobic* OR move OR bicycl* |
| **Species** | Humans *(screened manually)* |
| **Language** | No limit |

# Table S5: Reasons for exclusion of studies from the systematic literature search, after full-text screening

| **Study** | **Reason for exclusion** |
| --- | --- |
| Alvurdu et al. 2022 [2] | Data reporting on physical fitness. |
| Amagliani et al. 2023 [3] | Validation of measurement instrument is lacking. |
| Androutsos et al. 2021 [4] | Validation of measurement instrument is lacking. |
| Basterfield et al. 2022 [5] | Data reporting on physical fitness. |
| Beck et al. 2022 [6] | Same study population as Schmidt et al. 2020 [7]. |
| Béghin et al. 2022 [8] | No pre-pandemic baseline. |
| Bothe et al. 2022 [9] | Validation of measurement instrument is lacking. |
| Braksiek et al. 2022 [10] | Same study population as Schmidt et al. 2020 [7]. |
| Bronikowska et al. 2021 [11] | Validation of measurement instrument is lacking. |
| Brzęk et al. 2021 [12] | Validation of measurement instrument is lacking. |
| Burdzovic et al. 2021 [13] | Validation of measurement instrument is lacking. |
| Carballo-Fazanes et al. 2022 [14] | Data reporting on physical fitness. |
| Carrillo-Diaz et al. 2021 [15] | Validation of measurement instrument is lacking. |
| Carvalho et al. 2021 [16] | Same study population as López-Bueno et al. 2020 [17]. |
| Cassar et al. 2021 [18] | Validation of measurement instrument is lacking. |
| Censi et al. 2022 [19] | Validation of measurement instrument is lacking. |
| Chambonnìere et al. 2021 [20] | Data reporting on physical fitness. |
| Chambonnìere et al. 2021 [21] | Validation of measurement instrument is lacking. |
| Cipolla et al. 2021 [22] | Validation of measurement instrument is lacking. |
| Çoban et al. 2022 [23] | Validation of measurement instrument is lacking. |
| Corica et al. 2022 [24] | Validation of measurement instrument is lacking. |
| Dauty et al. 2021 [25] | Data reporting on physical fitness. |
| Derigny et al. 2022 [26] | Validation of measurement instrument is lacking. |
| Di Renzo et al. 2020 [27] | Validation of measurement instrument is lacking. |
| Dragun et al. 2021 [28] | Validation of measurement instrument is lacking. |
| Dzielska et al.2021 [29] | Validation of measurement instrument is lacking. |
| Eberhardt et al. 2022 [30] | Data reporting on physical fitness. |
| Eisenburger et al. 2022 [31] | Validation of measurement instrument is lacking. |
| Farello et al. 2022 [32] | Validation of measurement instrument is lacking. Only 2 questions of the validated YAP-27 instrument were used. |
| Fehr et al. 2022 [33] | No reporting of detailed values for pre-pandemic and during pandemic measurement. |
| Fillon et al. 2021 [34] | Validation of measurement instrument is lacking. |
| Francisco et al. 2020 [35] | Validation of measurement instrument is lacking. |
| Frömel et al. 2022 [36] | Validation of measurement instrument is lacking. |
| Füzéki et al. 2021 [37] | Validation of measurement instrument is lacking. No use of the complete validated EHIS-PAQ instrument. |
| Galhardo et al. 2021 [38] | Validation of measurement instrument is lacking. |
| Galluccio et al. 2021 [39] | Validation of measurement instrument is lacking. |
| García-Alonso et al. 2022 [40] | Data reporting on physical fitness. |
| Genin et al. 2021 [41] | Validation of measurement instrument is lacking. |
| Gentili et al. 2021 [42] | Validation of measurement instrument is lacking. |
| Gilic et al. 2021 [43] | Same study population as Geets Kesic et al. 2021 [44] |
| Gilic et al. 2020 [45] | Validation of measurement instrument is lacking. |
| Gobbi et al. 2020 [46] | Validation of measurement instrument is lacking. |
| Gomes et al. 2023 [47] | Validation of measurement instrument is lacking. |
| Greier et al. 2021 [48] | Validation of measurement instrument is lacking. |
| Hall-Lopez et al. 2021 [49] | Validation of measurement instrument is lacking. |
| Heymsfield et al. 2020 [50] | Validation of measurement instrument is lacking. |
| Humer et al. 2022 [51] | Validation of measurement instrument is lacking. |
| Iughetti et al. 2022 [52] | Validation of measurement instrument is lacking. |
| Jarnig et al. 2021 [53] | Data reporting on physical fitness. |
| Jarnig et al. 2022 [54] | Data reporting on physical fitness. |
| Jarnig et al. 2022 [55] | Data reporting on physical fitness. |
| Jarnig et al. 2022 [56] | Data reporting on physical fitness. |
| Jaskulska et al. 2022 [57] | Validation of measurement instrument is lacking. |
| Jurak et al. 2021 [58] | No data reporting on physical activity. |
| Katona et al. 2022 [59] | Same population as in Katona et al. 2021 [60] and validation of measurement instrument is lacking. |
| Katona et al. 2021 [60] | Validation of measurement instrument is lacking. |
| Kuygun Karci et al. 2022 [61] | Validation of measurement instrument is lacking. |
| Kołota et al. 2021 [62] | Validation of measurement instrument is lacking. |
| Kołota et al. 2022 [63] | Validation of measurement instrument is lacking. |
| Konstantinou et al. 2021 [64] | Validation of measurement instrument is lacking. |
| Lopez-Bueno et al. 2020 [17] | Validation of measurement instrument is lacking. |
| Lopez-Bueno et al. 2021 [65] | Validation of measurement instrument is lacking. |
| López-Gil et al. 2021 [66] | Validation of measurement instrument is lacking. |
| Martinko et al. 2022 [67] | Validation of measurement instrument is lacking. Only data for the complete SLOfit test were reported. |
| Maugeri et al. 2020 [68] | Validation of measurement instrument is lacking. |
| Medina Cantillo et al. 2022 [69] | Validation of measurement instrument is lacking. |
| Minuto et al. 2021 [70] | Validation of measurement instrument is lacking. |
| Morgül et al. 2020 [71] | Validation of measurement instrument is lacking. |
| Ng et al. 2020 [72] | Validation of measurement instrument is lacking. |
| Nigg et al. 2021 [73] | Same study population as Schmidt et al. 2020 [7]. |
| Nyberg et al. 2022 [74] | Validation of measurement instrument is lacking. |
| Delisle Nyström et al. 2020 [75] | Validation of measurement instrument (questionnaire) is lacking and ActiGraph data were only available for during pandemic not pre-prandemic. |
| Okely et al. 2021 [76] | Validation of measurement instrument is lacking. |
| Orgilés et al. 2020 [77] | Validation of measurement instrument is lacking. |
| Pajek et al. 2022 [78] | No data reporting on physical activity. |
| Palermi et al. 2022 [79] | Validation of measurement instrument is lacking. |
| Peralta et al. 2022 [80] | Validation of measurement instrument is lacking. |
| Pietrobelli et al. 2020 [81] | Validation of measurement instrument is lacking. |
| Pietrobelli et al. 2022 [82] | Validation of measurement instrument is lacking. |
| Planinsec et al. 2022 [83] | Validation of measurement instrument is lacking. |
| Pombo et al. 2021 [84] | Validation of measurement instrument is lacking. |
| Pombo et al. 2021 [85] | No data reporting on physical activity. |
| Pucsok et al. 2021 [86] | No data reporting on physical activity. |
| Pyšná et al. 2022 [87] | Validation of measurement instrument is lacking. |
| Ramos Álvarez et al. 2021 [88] | Data reporting on physical fitness. |
| Rua-Alonso et al. 2022 [89] | No data reporting on physical activity. |
| Rucinska et al. 2022 [90] | Validation of measurement instrument is lacking. |
| Sánchez-Sánchez et al. 2020 [91] | Validation of measurement instrument is lacking. |
| Schmidt et al. 2022 [92] | Validation of measurement instrument is lacking. |
| Sekulic et al. 2021 [93] | Same study population as Geets Kesic et al. 2021 [44] |
| Shneor et al. 2021 [94] | Validation of measurement instrument is lacking. |
| Siachpazidou et al. 2021 [95] | Validation of measurement instrument is lacking. |
| Sport England 2022 [96] | Validation of measurement instrument is lacking. |
| Sunda et al. 2021 [97] | No data reporting on physical activity. |
| Szabó et al. 2020 [98] | Validation of measurement instrument is lacking. |
| Szczepańska et al. 2022 [99] | Validation of measurement instrument is lacking. |
| Tatlibal et al. 2021 [100] | Validation of measurement instrument is lacking. |
| Theis et al. 2021 [101] | Validation of measurement instrument is lacking. |
| Thomas et al. 2021 [102] | Validation of measurement instrument is lacking. |
| Tornaghi et al. 2021 [103] | Validation of measurement instrument is lacking. |
| Tsoukos et al. 2022 [104] | No data reporting on physical activity. |
| Turan et al. 2021 [105] | Validation of measurement instrument is lacking. |
| Ventura et al. 2021 [106] | Validation of measurement instrument is lacking. |
| Vuković et al. 2021 [107] | Validation of measurement instrument is lacking. |
| Welling et al. 2021 [108] | Validation of measurement instrument is lacking. |
| Wessely et al. 2022 [109] | Validation of measurement instrument is lacking. |
| Wunsch et al. 2021 [110] | Same study population as Schmidt et al. 2020 [7]. |
| Zachurzok et al. 2021 [111] | Same study population as Zachurzok et al. 2022 [112] |
| Zachurzok et al. 2022 [112] | Validation of measurement instrument is lacking. |
| Zorcec et al. 2020 [113] | Validation of measurement instrument is lacking. |

# Table S6: Data conversion

| **Reported measurement** | **Conversion in minutes/day** |
| --- | --- |
| Device-based measurements reported as minutes/hour [114]. | Multiplying the mean x 12 and standard deviation (SD) x sqrt [12] (activity times were estimated based on sleep recommendations for CA [115]). |
| Subdivision into weekday and weekend [116, 117]. | Authors were asked to provide combined data; alternatively, we used a 5:2 ratio weight to aggregate the data. |
| Data of one study population with two pandemic measurement points [7, 118]. | Aggregation using weighted factors according to the sample size before included in the meta-analysis. |
| Only gender-stratified reported data [119]. | Pooling was performed before adding them to the meta-analysis |
| Only CI for means were given. | Calculation of standard deviation [120]. |

# Table S7: Criteria for grading evidence according to Grading of Recommendations, Assessment, Development and Evaluations (GRADE)

Developed under consideration of the criteria reported by Schünemann et al. 2013 [121], Schünemann et al. 2019 [122] and Morgan et al. 2019 [123].

| **Domain** | **Application in this review** | **Interpretation** | **GRADE assessment** |
| --- | --- | --- | --- |
| *Reasons for considering lowering certainty* | |  |  |
| Risk of Bias (RoB) | Based on the RoB assessment using the RoB instrument for non-randomized studies of exposures [123]. | 1. Most information is from results at low risk of bias. 2. Most information is from results at low risk of bias or with some concerns; potential limitations are unlikely to lower confidence in the estimate of effect. 3. Most information is from results at low risk of bias or with some concerns; potential limitations are likely to lower confidence in the estimate of effect. 4. The proportion of information from results at high risk of bias is sufficient to affect the interpretation of results; crucial limitation for one criterion, or some limitations for multiple criteria, sufficient to lower confidence in the estimate of effect. 5. The proportion of information from results at high risk of bias is sufficient to affect the interpretation of results; crucial limitation for one or more criteria sufficient to substantially lower confidence in the estimate of effect. | 1. No serious limitations, not downgrade. 2. No serious limitations, not downgrade. 3. Serious limitations, downgrade 1 level. 4. Serious limitations, downgrade 1 level. 5. Very serious limitations, downgrade 2 levels. |
| Inconsistency | Existence of heterogeneity, if   1. Wide variance of point estimates across studies 2. Minimal or no overlap of confidence intervals (CI) 3. Statistical criteria: chi^2^ test and I² statistic    - Significant p-value in chi² test 🡪 indication of heterogeneity    - I² statistic    - < 40% 🡪 heterogeneity may be low    - 30-60% 🡪 heterogeneity may be moderate    - 50-90% 🡪 heterogeneity may be substantial    - 75-100% 🡪 heterogeneity may be considerable | - - - 1. No wide variance of point estimates, minimal or no overlap of CI, chi² test not significant,  I² <60%.       2. Chi² test significant, I² test >50%, further analysis via subgroup analysis, sensitivity analysis, meta-regression analysis.       3. Chi² test significant, I² test >50%, no further analysis. | 1. No serious limitations, not downgrade. 2. Serious limitations, downgrade 1 level. 3. Very serious limitations, downgrade 2 levels. |
| Indirectness | Applicability of the evidence to the relevant research question. Fitting of the characteristics of the included studies to the predefined PECO scheme. | - - - 1. Studies address exactly the research question.       2. Studies indirectly (indirect comparisons) or imprecisely (restricted version of the main review question) address the research question, but an adequate transfer is possible.       3. Studies indirectly (indirect comparisons) or imprecisely (restricted version of the main review question) address the research question, but an adequate transfer is not possible. | 1. No serious limitations, not downgrade. 2. Serious limitations, downgrade 1 level. 3. Very serious limitations, downgrade 2 levels. |
| Imprecision | Risks of random errors because of small sample sizes, broad CIs or inclusion of the “no effect line” in 95% CI | - - - 1. Broad sample size with small to moderate confidence intervals and no overlap of the “no effect line” (mean/SMD=0, OR/RR=1) regarding the 95% CI of the pooled effect.       2. Moderate to broad sample size, with moderate confidence intervals and overlap of the line of no effect of the 95% CI.       3. Small sample size, with moderate to broad confidence intervals and overlap of the line of no effect of the 95% CI. | 1. No serious limitations, not downgrade. 2. Serious limitations, downgrade 1 level. 3. Very serious limitations, downgrade 2 levels. |
| Publication bias | Under-estimation or over-estimation of the underlying beneficial or harmful effect due to the selective publication of studies if   - - Asymmetrical funnel plot   - Significant Egger’s Test when at least 10 studies | 1. No funnel plot asymmetry and no significance in Egger’s Test. 2. Funnel plot asymmetry and/or significance in Egger’s Test. | 1. No serious limitations, not downgrade. 2. Serious limitations, downgrade 1 level. |
|  |  |  |  |
| *Reasons for considering upgrade certainty* | |  |  |
| Large effect | Rating up when effects in observational studies are sufficiently large, particularly if they occur over short periods of time. | 1. RR >2 or RR <0.5 2. RR >5 or RR <0.2   *Note from the GRADE Handbook [121]: these rules apply when effect measure is expressed as relative risk (RR) or hazard ratio (HR). They cannot always be applied when the effect measure is expressed as odds ratio (OR). We suggest converting OR to RR and only then assessing the magnitude of an effect.* | - - - 1. May upgrade 1 level.       2. May upgrade 2 levels. |
| Dose-response | Rating up for a dose-response gradient if effect estimates are higher when pandemic related restrictions are rigorous, measured by the proxy variables “Oxford COVID-19 Stringency Index” and the “School Closure Index”. | 1. No/small differences in pandemic-related restrictions and no substantial subgroup differences. 2. Effect estimates for severe pandemic-related restrictions higher with substantial subgroup differences to moderate/lead restrictions. | 1. No upgrade. 2. May upgrade 1 level. |
| All plausible confounding and bias | All plausible residual confounding from observational studies may be working to reduce the demonstrated effect or increase the effect, if no effect was observed. | 1. No underestimation of an apparent treatment effect. 2. Underestimate of an apparent treatment effect. | 1. No upgrade. 2. May upgrade 1 level. |
|  |  |  |  |
| **Certainty of evidence** | **High – Moderate – Low – Very low** | | |

# Table S8: Evidence profile for grading evidence according to Grading of Recommendations, Assessment, Development and Evaluations (GRADE)

|  |  | ***Reasons for considering lowering certainty*** | | | | |  | ***Reasons for considering upgrade certainty*** | | |  |
| --- | --- | --- | --- | --- | --- | --- | --- | --- | --- | --- | --- |
| Outcome | Number of studies | Risk of bias | Inconsistency | Indirectness | Imprecision | Publication bias | **AFTER DOWNGRADE** Certainty of evidence | Large effect | Dose response | All plausible confounding and bias | **AFTER UPGRADE** Certainty of evidence |
| **Total physical activity**  All studies:  SMD, -0.57 (95% CI,  -0.95 to -0.20) | 14 studies | Downgrade by **-0.5 points,** because some studies had a high or very high risk of bias | Downgrade by **-1 point**, because of considerable heterogeneity indicated by I^2^=96% and the 95% PI: -1.99 to 0.84 | No downgrade | No downgrade | Downgrade by **-0.5 points,** because visual inspection of funnel plot suggests asymmetry and is supported by an almost statistically significant test (p=0.052) | **Low** | No upgrade | No upgrade | No upgrade | **Low** |
| **Moderate-to-vigorous physical activity**  All studies:  SMD, -0.43 (95% CI,  -0.75 to -0.10) | 12 studies | Not downgrade | Downgrade by **-1 point**, because of considerable heterogeneity indicated by I^2^=92% and the 95% PI: -1.52 to 0.66 | No downgrade | No downgrade | Downgrade by **-1 point,** because visual inspection of funnel plot suggests asymmetry and is supported by a statistically significant test (p=0.02) | **Low** | No upgrade | No upgrade | No upgrade | **Low** |
| **Sports activity**  3 studies (no pooling):  SMD, 0.21 (95% CI, 0.14 to 0.28) [26]  SMD, -0.83 (95% CI, -0.90 to -0.75) [21]  SMD, -0.19 (95% CI, -0.37 to -0.01) [47] | 3 studies | Downgrade by **-1 point**, because one study had a high risk of bias and two studies are from the same population | Downgrade by **-1 point**, because of differences in point estimate and no overlap of 95% CI | No downgrade | No downgrade | No downgrade | **Low** | No upgrade | No upgrade | No upgrade | **Low** |

# Table S9: Summary of effect estimates

| **STUDY INFO** | **REPORTED Effect estimate** | | | **(converted) effect estimate*** | | | **Risk of bias** |
| --- | --- | --- | --- | --- | --- | --- | --- |
| **First author, year** | **Diagnostic instrument,**  **validation study** | **Adjustment, subgroups** | **Type of outcome measurement, additional information** | **During pandemic measure, mean (SD) N or n/N** | **Pre-pandemic measure, mean (SD) N or n/N** | **Standardized mean difference or  risk ratio (95% CI)** |  |
| **Bosnia and Herzegovina** | |  |  |  |  |  |  |
| Geets Kesic, 2021 [44] | PAQ for Adolescents (PAQ-A), [124, 125] | No adjustment, no subgroups | Self-reported **total** physical activity,  *Additional data were requested from the authors; no data were provided* | **Total (14-18 y):**  2.50 (0.82) 859 | **Total (14-18 y):**  2.76 (0.79) 859 | **Total (11-19 y):**  -0.32 (-0.42 to -0.23) | **High** |
| **Croatia** | |  |  |  |  |  |  |
| Sekulic, 2020 [126] | PAQ for Adolescents (PAQ-A), [124, 125] | No adjustment, subgroup by gender | Self-reported **total** physical activity,  *Additional data were requested from the authors; no data were provided* | **Total (15-18 y):**  2.67 (0.60) 388  **Female (15-18 y):**  2.59 (0.90) 126  **Male (15-18 y):**  2.79 (0.82) 262 | **Total (15-18 y):**  2.99 (0.70) 388  **Female (15-18 y):**  2.71 (0.66) 126  **Male (15-18 y):**  3.10 (0.78) 262 | **Total (15-18 y):**  -0.49 (-0.63 to -0.35)  **Female (15-18 y):**  -0.15 (-0.40 to 0.10)  **Male (15-18 y):**  -0.39 (-0.56 to -0.21) | **Some concerns** |
| Zenic, 2020 [127] | PAQ for Adolescents (PAQ-A), [124, 125] | No adjustment, subgroup by rural/urban region | Self-reported **total** physical activity,  *No further data requested* | **Total (11-17 y):**  2.63 (0.68) 823 | **Total (11-17 y):**  2.97 (0.61) 823 | **Total (11-17 y):**  -0.53 (-0.62 to -0.43[128]) | **High** |
| **Czech Republic** | |  |  |  |  |  |  |
| Štveráková, 2021 [129] | PAQ for Children (PAQ-C), [130] | No adjustment, subgroup by gender | Self-reported **total** physical activity,  *No further data requested* | **Total (8-12 y):**  2.30 (0.66) 98  **Female (8-12 y):**  2.29 (0.64) 56  **Male (8-12 y):**  2.32 (0.69) 42 | **Total (8-12 y):**  2.69 (0.59) 206  **Female (8-12 y):**  2.68 (0.56) 100  **Male (8-12 y):**  2.69 (0.62) 106 | **Total (8-12 y):**  -0.63 (-0.88 to -0.39)  **Female (8-12 y):**  -0.68 (-1.02 to -0.35)  **Male (8-12 y):**  -0.57 (-0.94 to -0.21) | **Some concerns** |
| **Germany** |  |  |  |  |  |  |  |
| Kurz, 2022 [12] | Bayer-Questionnaire,  [131] | Adjustment (child age, child gender, year of school enrollment, maternal educational attainment), subgroups by gender | Parent-reported **total** physical activity  *Additional data were provided by authors* | **Total (6-7 y):**  1.83 (2.91) 63³  **Female (6-7 y):**  1.75 (2.42) 32  **Male (6-7 y):**  1.90 (3.38) 31 | **Total (6-7 y):**  1.52 (2.97) 296³  **Female (6-7 y):**  1.51 (3.04) 157  **Male (6-7 y):**  1.54 (2.89) 139 | **Total (6-7 y):**  0.10 (-0.17 to 0.38)³  **Female (6-7 y):**  0.08 (-0.30 to 0.46)  **Male (6-7 y):**  0.12 (-0.27 to 0.51) | **High** |
| Schmidt, 2020 [26] | MoMo-PAQ, [132] | No adjustment, subgroups by gender and age | Self- and parent-reported **moderate-to-vigorous** physical activity | **Total (4-17 y):**  4.70 (2.00) 1,711²  **Female (4-17y):**  4.60 (1.90) 852²  **Male (4-17 y):**  4.80 (2.00) 859² | **Total (4-17 y):**  4.30 (1.80) 1,711²  **Female (4-17 y):**  4.10 (1.80) 852²  **Male (4-17 y):**  4.40 (1.80) 859² | **Total (4-17 y):**  0.21 (0.14 to 0.28)²  **Female (4-17 y):**  0.27 (0.17 to 0.37)²  **Male (4-17 y):**  0.21 (0.12 to 0.30)² | **Some concerns** |
|  |  |  | Self- and parent-reported **sports** activity  *Additional data were provided by authors* | **Total (4-17 y):**  24.30 (36.20) 1,711²  **Female (4-17y):**  24.00 (33.40) 852²  **Male (4-17 y):**  24.60 (38.80) 859² | **Total (4-17 y):**  34.90 (26.00) 1,711²  **Female (4-17 y):**  33.50 (25.10) 852²  **Male (4-17 y):**  36.30 (26.80) 859² | **Total (4-17 y):**  -0.34 (-0.40 to -0.27)²  **Female (4-17 y):**  -0.32 (-0.42 to -0.23)²  **Male (4-17 y):**  -0.35 (-0.45 to -0.26)² |  |
| Schmidt, 2021 [21] | MoMo-PAQ, [132] | No adjustment, subgroups by gender and age | Self- and parent-reported **moderate-to-vigorous** physical activity | **Total (4-17 y):**  3.83 (1.99) 1,483²  **Female (4-17y):**  3.78 (1.92) 728²  **Male (4-17 y):**  3.88 (2.05) 755² | **Total (4-17 y):**  4.30 (1.80) 1,711²  **Female (4-17 y):**  4.10 (1.80) 852²  **Male (4-17 y):**  4.40 (1.80) 859² | **Total (4-17 y):**  -0.25 (-0.32 to -0.18)²  **Female (4-17 y):**  -0.17 (-0.27 to -0.07)²  **Male (4-17 y):**  -0.27 (-0.37 to -0.17)² | **Some concerns** |
|  |  |  | Self- and parent-reported **sports** activity  *Additional data were provided by authors* | **Total (4-17 y):**  13.26 (26.40) 1,483²  **Female (4-17y):**  13.35 (25.71) 728²  **Male (4-17 y):**  13.16 (27.07) 755² | **Total (4-17 y):**  34.90 (26.00) 1,711²  **Female (4-17 y):**  33.50 (25.10) 852²  **Male (4-17 y):**  36.30 (26.80) 859² | **Total (4-17 y):**  -0.83 (-0.90 to -0.75)²  **Female (4-17 y):**  -0.79 (-0.90 to -0.69)²  **Male (4-17 y):**  -0.86 (-0.96 to -0.76)² |  |
| **Ireland** |  |  |  |  |  |  |  |
| O’Kane, 2021 [128] | PACE+, [133] | No adjustment, no subgroups | Self-reported **moderate-to-vigorous** physical activity  *Additional data were requested from the authors; no data were provided* | **Total (12-14 y):**  3.50 (2.59) 94^4^ | **Total (12-14 y):**  4.00 (1.48) 281^4^ | **Total (12-14 y):**  -0.24 (-0.52 to 0.05)^4^ | **Some concerns** |
| **Italy** |  |  |  |  |  |  |  |
| Mastorci, 2021 [134] | PAQ for Children (PAQ-C), [130] | No adjustment, subgroups by gender | Self-reported **total** physical activity  *No further data requested* | **Total (10-14 y):**  2.70 (0.80) 1,289  **Female (10-14 y):**  2.60 (0.70) 667  **Male (10-14 y):**  2.70 (0.80) 622 | **Total (10-14 y):**  2.6 (0.70) 1,289  **Female (10-14 y):**  2.50 (0.70) 667  **Male (10-14 y):**  2.80 (0.70) 622 | **Total (10-14 y):**  0.13 (0.06 to 0.21)  **Female (10-14 y):**  0.20 (-0.24 to 0.63)  **Male (10-14 y):**  -0.13 (-0.24 to -0.03) | **Some concerns** |
| Dallolio, 2022 [135] | PAQ for Children (PAQ-C), [130] | No adjustment, subgroups by gender | Self-reported **total** physical activity (min/day)  *No further data requested* | **Total (8-11 y):**  2.19 (0.57) 52  **Female (8-11 y):**  2.08 (0.64) 29  **Male (8-11 y):**  2.25 (0.60) 48 | **Total (8-11 y):**  3.06 (0.75) 52  **Female (8-11 y):**  2.69 (0.78) 29  **Male (8-11 y):** 3.34 (0.72) 48 | **Total (8-11 y):**  -1.30 (-1.72 to -0.87)  **Female (8-11 y):**  -0.84 (-1.38 to -0.30)  **Male (8-11 y):**  -1.63 (-2.10 to -1.17) | **Some concerns** |
|  | ActiGraph GT3X, [136–138] | No adjustment, subgroups by gender | Accelerometer measured **moderate-to-vigorous** physical activity (min/day)  *No further data requested* | **Total (8-11 y):**  40.13 (14.18) 77  **Female (8-11 y):**  37.30 (13.01) 29  **Male (8-11 y):**  41.83 (14.70) 48 | **Total (8-11 y):**  55.44 (19.10) 77  **Female (8-11 y):**  45.63 (16.18) 29  **Male (8-11 y):** 61.38 (19.58) 48 | **Total (8-11 y):**  -15.31 (-20.62 to -10.00)  **Female (8-11 y):**  -0.56 (-1.09 to -0.03)  **Male (8-11 y):**  -1.12 (-1.55 to -0.69) |  |
| **Netherlands** | |  |  |  |  |  |  |
| ten Velde, 2021 [139] | ActiGraph GT3X, [136–138] | No adjustment, subgroups by gender | Accelerometer measured **moderate-to-vigorous** physical activity (min/day)  *No further data requested* | **Total (7-12 y):**  48.00 (18.00) 64  **Female (7-12 y):**  No information  **Male (7-12 y):**  No information | **Total (7-12 y):**  65.00 (18.00) 64  **Female (7-12 y):**  No information  **Male (7-12 y):**  No information | **Total (7-12 y):**  -17.00 (-23.24 to -10.76)  **Female (7-12 y),** mean±SD**:**  -18±20  **Male (7-12 y),** mean±SD**:**  -15±16 | **Some concerns** |
| **Poland** |  |  |  |  |  |  |  |
| Kołota, 2021 [63] | Question regarding number of days with >60 minute of MVPA, including "include any activity that increases their heart rate and makes them get out of breath […]", [140] | No adjustment, no subgroups | Self-reported **moderate-to-vigorous** physical activity  (not meeting recommendation)  *No further data requested* | **Total (10-16 y):**  542/1334 | **Total (10-16 y):**  501/1334 | **Total (10-16 y):**  1.08 (0.98 to 1.19) | **High** |
| Łuszczki, 2021 [141] | Question regarding number of days with >60 minute of MVPA, including "increases your heart rate and makes you get out of breath some of the time […]", [140] | No adjustment, no subgroups | Self- and parent-reported **moderate-to-vigorous** physical activity  *No further data requested* | **Total (6-15 y):**  3.30 (2.07) 376 | **Total (6-15 y):**  3.89 (1.89) 641 | **Total (6-15 y):**  -0.24 (-0.52 to 0.05) | **High** |

| **Portugal** |  |  |  |  |  |  |  |
| --- | --- | --- | --- | --- | --- | --- | --- |
| Mercê, 2022 [117] | Pictorial Children's Physical Activity Questionnaire, [142] | No adjustment, no subgroups | Self-reported **total** physical activity  *Additional data were provided by authors* | **Total (5-17 y):**  2.27 (0.97) 61^1^ | **Total (5-17 y):**  2.88 (0.94) 61^1^ | **Total (5-17 y):**  -0.64 (-1.26 to -0.02)^1^ | **Very high** |
| **Slovenia** |  |  |  |  |  |  |  |
| Blazević, 2021 [143] | PAQ for Adolescents (PAQ-A), [124, 125] | No adjustment, subgroups by gender | Self-reported **total** physical activity  *No further data requested* | **Total (15-17 y):**  2.81 (0.81) 209  **Female (15-17 y):**  2.80 (0.70) 120  **Male (15-17 y):**  2.90 (0.85) 89 | **Total (15-17 y):**  3.04 (0.61) 209  **Female (15-17 y):**  2.78 (0.65) 120  **Male (15-17 y):** 3.13 (0.76) 89 | **Total (15-17 y):**  -0.32 (-0.51 to -0.13)  **Female (15-17 y):**  0.03 (-0.22 to 0.28)  **Male (15-17 y):**  -0.28 (-0.58 to 0.01) | **Some concerns** |
| Morrison, 2021 [144] | School Health Action, Planning, and Evaluation System (SHAPES), [145] | No adjustment, subgroups by gender | Self-reported **moderate-to-vigorous** physical activity  *No further data requested* | **Total (8-11 y):**  72.20 (35.00) 62  **Female (8-11 y):**  75.40 (34.70) 31  **Male (8-11 y):**  69.1 (35.70) 31 | **Total (8-11 y):**  118.10 (56.00) 62  **Female (8-11 y):**  111.9 (62.50) 31  **Male (8-11 y):** 124.2 (49.00) 31 | **Total (8-11 y):**  -15.31 (-20.62 to -10.00)  **Female (8-11 y):**  -0.71 (-1.23 to -0.20)  **Male (8-11 y):**  -1.27 (-1.82 to -0.72) | **Some concerns** |
| **Spain** |  |  |  |  |  |  |  |
| Alonso-Martinez, 2021 [146] | GENEActiv tri-axial accelerometer, [147, 148] | No adjustment, no subgroups | Accelerometer measured **total** physical activity (min/day) | **Total (4-6 y):**  303.60 (76.50) 21 | **Total (4-6 y):**  346.90 (54.60) 21 | **Total (4-6 y):**  -0.64 (-1.26 to -0.02) | **Very high** |
|  |  |  | Accelerometer measured **moderate-to-vigorous** physical activity (min/day)  *No further data requested* | **Total (4-6 y):**  74.6 (26.00) 21 | **Total (4-6 y):**  91.60 (26.70) 21 | **Total (4-6 y):**  -17.00 (-32.94 to -1.06) |  |
| García-Alonso, 2022 [40] | GENEActiv tri-axial accelerometer, [147, 148] | No adjustment, subgroups by gender | Accelerometer measured **total** physical activity (min/day) | **Total (4-7 y):**  375.58 (63.11) 86  **Female (4-7 y):**  375.46 (73.08) 41  **Male (4-7 y):**  375.68 (53.27) 45 | **Total (4-7 y):**  366.23 (72.77) 86  **Female (4-7 y):**  359.90 (83.35) 41  **Male (4-7 y):** 372.00 (61.98) 45 | **Total (4-7 y):**  0.14 (-0.90 to -0.04)  **Female (4-7 y):**  0.20 (-0.24 to 0.63)  **Male (4-7 y):**  0.06 (-0.35 to 0.48) | Some concerns |
|  |  |  | Accelerometer measured **moderate-to-vigorous** physical activity (min/day)  *Additional data were provided by authors* | **Total (4-7 y):**  103.23 (34.59) 86  **Female (4-7 y):**  94.33 (29.89) 41  **Male (4-7 y):**  111.34 (36.85) 45 | **Total (4-7 y):**  89.46 (34.57) 86  **Female (4-7 y):**  78.33 (32.32) 41  **Male (4-7 y):** 99.59 (33.75) 45 | **Total (4-7 y):**  0.40 (0.09 to 0.70)  **Female (4-7 y):**  0.51 (0.07 to 0.95)  **Male (4-7 y):**  0.33 (-0.09 to 0.75) |  |
| Medrano, 2021 [149] | ActiGraph (no further information) | No adjustment, no subgroups | Accelerometer measured **total** physical activity (min/day) | **Total (8-16 y):**  63.00 (39.00) 106 | **Total (8-16 y):**  154.00 (40.00) 106 | **Total (8-16 y):**  -2.30 (-2.64 to -1.95) | High |
|  |  |  | Accelerometer measured **moderate-to-vigorous** physical activity (not meeting recommendation)  *Additional data were requested from the authors; no data were provided* | **Total (8-16 y):**  134/224 | **Total (8-16 y):**  45/89 | **Total (8-16 y):**  1.18 (0.94 to 1.49) |  |
| Tapia-Serrano, 2022 [150] | PAQ for Adolescents (PAQ-A), [124, 125] | No adjustment, subgroups by gender | Self-reported **total** physical activity  *Additional data were provided by authors* | **Total (15-17 y):**  2.37 (0.54) 501  **Female (15-17 y):**  2.26 (0.50) 277  **Male (15-17 y):**  2.51 (0.55) 224 | **Total (15-17 y):**  2.54 (0.61) 844  **Female (15-17 y):**  2.40 (0.57) 360  **Male (15-17 y):** 2.64 (0.62) 484 | **Total (15-17 y):**  -0.29 (-0.40 to -0.18)  **Female (15-17 y):**  -0.26 (-0.42 to -0.10)  **Male (15-17 y):**  -0.22 (-0.38 to -0.06) | Some concerns |
| **Sweden** |  |  |  |  |  |  |  |
| Chen, 2022 [46] | WHO HBSC physical activity questionnaire, [151] | No adjustment, subgroups by gender | Self-reported **moderate-to-vigorous** physical activity  *Additional data were provided by authors* | **Total (~13 y):**  3.90 (1.90) 583  **Female (~13 y):**  3.60 (1.80) 310  **Male (~13 y):**  4.30 (1.90) 273 | **Total (~13 y):**  4.10 (1.70) 583  **Female (~13 y):**  3.90 (1.80) 310  **Male (~13 y):** 4.20 (1.70) 273 | **Total (~13 y):**  -0.11 (-0.23 to 0.00)  **Female (~13 y):**  -0.17 (-0.32 to -0.01)  **Male (~13 y):**  -0.06 (-0.11 to 0.22) | Some concerns |
| **Switzerland** |  |  |  |  |  |  |  |
| Zehnder, 2022 [47] | German Physical Activity, Exercise and Sport Questionnaire  (BSA-F), [152] | No adjustment, no subgroups | Self-reported **sports** activity (min/week)  *No further data requested* | **Total (7-16 y):**  294.20 (294.90) 237 | **Total (7-16 y):**  349.80 (279.86) 237 | **Total (7-16 y):**  -0.19 (-0.37 to -0.01) | High |
| **United Kingdom** | |  |  |  |  |  |  |
| Bingham, 2021 [153] | PP: PAQ for Children (PAQ-C) [130]; DP: Youth Activity Profile- English Youth Version (YAP) [154] | No adjustment, no subgroups | Self-reported **total** physical activity  (not meeting recommendation)  *No further data requested* | **Total (9-13 y):**  455/638 *(measured with Youth Activity Profile)* | **Total (9-13 y):**  194/634  *(measured with PAQ for Children)* | **Total (9-13 y):**  2.33 (2.05 to 2.65) | High |
| James, 2021 [155] | HAPPEN survey [140] | No adjustment, subgroups by gender and free school meal | Self-reported **moderate-to-vigorous** physical activity (meeting recommendation)  *Additional data were provided by authors* | **Total (8-11 y):**  27.32%  **Female (8-11 y):**  24.53%  **Male (8-11 y):**  29.64%  **Free school meal (8-11 y):**  15.10%  **No free school meal  (8-11y):**  28.23% | **Total (8-11 y):**  22.78%  **Female (8-11 y):**  18.32%  **Male (8-11 y):** 26.90%  **Free school meal (8-11 y):**  20.54%  **No free school meal  (8-11y):**  23.95% | **Total (8-11 y):**  4.54% (0.93% to 8.14%)  **Female (8-11 y):**  6.21% (0.13% to 12.60%)  **Male (8-11 y):**  2.74% (-3.60% to 9.25%)  **Free school meal (8-11 y):**  -5.44% (-7.83% to 15.70%)  **No free school meal  (8-11y):**  4.28% (-0.52% to 9.12%) | Some concerns |
| Salway, 2022 [116] | ActiGraph wGT3X-BT [138] | No adjustment, subgroups by gender | Accelerometer measured **moderate-to-vigorous** physical activity (min/day) | **Total (10-11 y):**  50.82 (38.90) 397^1^  **Female (10-11 y):**  44.50 (31.60) 189^1^  **Male (10-11 y):**  60.20 (27.50) 193^1^ | **Total (10-11 y):**  56.85 (49.56) 1296^1^  **Female (10-11 y):**  51.20 (42.60) 680^1^  **Male (10-11 y):** 66.01 (53.3) 616^1^ | **Total (10-11 y):**  -0.13 (-0.24 to -0.01)  **Female (10-11 y):**  -0.17 (-0.33 to -0.00)  **Male (10-11 y):**  -0.12 (-0.28 to 0.04) | Some concerns |
|  |  | Adjustment for seasonality, accelerometer wear time, COVID restrictions, child age and household education; ‘Total‘ model additionally adjusted for child gender.  Subgroups by gender | Accelerometer measured **moderate-to-vigorous** physical activity (min/day) |  |  | **Total (10-11 y),** estimate (95% CI)**:**  -4.90 (-9.09 to -0.70)  **Female (10-11 y),** estimate (95% CI)**:**  -5.11 (-10.16 to -0.05)  **Male (10-11 y),** estimate (95% CI)**:**  -4.71 (-9.54 to 0.12) |  |
| Sheldrick, 2022 [114] | ActiGraph GT9X [156] | No adjustment, no subgroups | Accelerometer measured **total** physical activity (min/h) | **Total (10-12 y):**  16.40 (4.30) 102^5^ | **Total (10-12 y):**  21.60 (4.80) 102^5^ | **Total (10-12 y):**  -1.14 (-1.43 to -0.84) | Some concerns |
|  |  |  | Accelerometer measured **moderate-to-vigorous** physical activity (min/h)  *Additional data were requested from the authors; no data were provided* | **Total (10-12 y):**  4.50 (2.00) 102^5^ | **Total (10-12 y):**  6.90 (2.40) 102^5^ | **Total (10-12 y):**  -1.08 (-1.38 to -0.79) |  |

^1^ Effect estimates of weekday and weekend were aggregated with a 5:2 ratio weight.

² Effect estimates were aggregated for meta-analysis by using weighted factors according to the sample size.

³ Effect estimates for female and male were pooled before adding to the meta-analysis.

^4^ Conversion of median (interquartile range) in mean (standard deviation) using the recommended Cochrane formula [120].

^5^ Conversion of measurement data from minutes/hour in minutes/day by multiplying the mean x 12 and SD x sqrt[12].

DP, during pandemic; h, hour; min, minutes; M, mean; n, events; N, total; NI, no information; PP, pre-pandemic; SD, Standard deviation; y, years of age

# Table S10: Meta-regression for total physical activity with categorical moderators

| **Categorical moderators** | **k** | **Estimate (95% CI)** | **Test of moderators** | **Test for residual heterogeneity** |
| --- | --- | --- | --- | --- |
| Risk of bias (some concerns vs high) | 14 | 0.23 (-0.55 to 1.01) | p=0.53 | p<0.0001 |
| Age (child vs adolescent) | 14 | -0.07 (-0.80 to 0.66) | P=0.85 | p<0.0001 |
| Symptom reporter (self vs accelerometer) | 14 | 0.57 (-0.23 to 1.38) | p=0.15 | p<0.0001 |
| Country | 14 | *Single values not listed* | p=0.99 | p<0.0001 |
| Stringency Index | 14 | *Not possible, all studies have an Oxford Stringency Index > 60* | | |
| School Closure Index (≥2 vs <2) | 14 | -0.58 (-1.62 to 0.47) | p=0.25 | p<0.0001 |
| Study design (cohort vs cross-sectional vs retrospective) | 14 | Cross-sectional:  0.39 (-0.59 to 1.37)  Retrospective: 0.03 (-1.56 to 1.62) | p=0.69 | p<0.0001 |

# Table S11: Meta-regression for total physical activity with continuous moderators

| **Continuous moderators** | **k** | **Estimate (95% CI)** | **Test of moderators** | **Test for residual heterogeneity** |
| --- | --- | --- | --- | --- |
| Time of measurement during pandemic | 14 | 0.005(-0.11 to 0.10) | p=0.92 | p<0.0001 |
| Publication year | 14 | 0.02 (-0.46 to 0.50) | p=0.93 | p<0.0001 |
| Stringency Index | 14 | 0.01 (-0.02 to 0.04) | p=0.33 | p<0.0001 |
| School Closure Index | 14 | -0.16 (-0.74 to 0.42) | p=0.56 | p<0.0001 |
| Sample size | 14 | 0.00 (-0.00 to 0.00) | p=0.18 | p<0.0001 |

# Table S12: Meta-regression for moderate-to-vigorous physical activity with categorical moderators

| **Categorical moderators** | **k** | **Estimate (95% CI)** | **Test of moderators** | | **Test for residual heterogeneity** |
| --- | --- | --- | --- | --- | --- |
| Risk of bias (some concerns vs high) | 11 | 0.01 (-0.92 to 0.94) | p=0.98 | | p<0.0001 |
| Age (child vs adolescent) | 11 | -0.11 (-0.95 to 0.73) | P=0.76 | | p<0.0001 |
| Symptom reporter (self vs accelerometer) | 11 | 0.11 (-0.61 to 0.82) | p=0.75 | | p<0.0001 |
| Country | 11 | *Single values not listed* | p=0.88 | | p<0.0001 |
| Stringency Index (>60 vs ≤60) | 11 | 0.33 (-0.81 to 1.47) | p=0.52 | p<0.0001 | |
| School Closure Index (≥2 vs <2) | 11 | -0.39 (-1.05 to 0.27) | p=0.21 | | p<0.0001 |
| Study design (cohort vs cross-sectional vs retrospective) | 11 | Cross-sectional:  0.30 (-0.62 to 1.22)  Retrospective: 0.29 (-0.99 to 1.55) | p=0.71 | | p<0.0001 |

# Table S13: Meta-regression for moderate-to-vigorous physical activity with continuous moderators

| **Continuous moderators** | **k** | **Estimate (95% CI)** | **Test of moderators** | **Test for residual heterogeneity** |
| --- | --- | --- | --- | --- |
| Time of measurement during pandemic | 11 | 0.02(-0.05 to 0.09) | p=0.50 | p<0.0001 |
| Publication year | 11 | 0.14 (-0.55 to 0.83) | p=0.67 | p<0.0001 |
| Stringency Index | 11 | -0.01 (-0.04 to 0.03) | p=0.69 | p<0.0001 |
| School Closure Index | 11 | -0.25 (-0.66 to 0.16) | p=0.20 | p<0.0001 |
| Sample size | 11 | 0.00 (-0.00 to 0.00) | p=0.16 | p<0.0001 |

# Table S14: Sensitivity analysis for total physical activity

| **Population** | **Comparison (number of studies per comparison group in parentheses)** | **Effect estimates  for all studies**  **SMD (95% CI); I²** | **Test for subgroup differences**  **Chi² (p-value)** |
| --- | --- | --- | --- |
| Total | Cohort studies (11) vs  Cross sectional studies (3) | -0.65 (-0.94 to -0.36); 97%  -0.28 (-0.61 to 0.05); 87% | 2.70 (p=0.10) |
| Total | Adjusted studies (1) vs  Unadjusted studies (13) | 0.10 (-0.17 to 0.38), ***only 1 study***  -0.61 (-0.85 to -0.37); 97% | **15.03 (p=0.0001)** |
| Total | Unconverted studies (12) vs  Converted studies (2) | -0.61 (-0.86 to -0.36); 98%  -0.26 (-0.98 to 0.47); 90% | 0.83 (p=0.36) |

# Table S15: Sensitivity analysis for moderate-to-vigorous physical activity

| **Population** | **Comparison (number of studies per comparison group in parentheses)** | **Effect estimates  for all studies**  **SMD (95% CI); I²** | **Test for subgroup differences**  **Chi² (p-value)** |
| --- | --- | --- | --- |
| Total | Cohort studies (11) vs  Cross sectional studies (3) vs  Retrospective studies (1) | -0.66 (-1.07 to -0.25); 94%  -0.17 (-0.40 to 0.06); 88%  -0.13 (-0.24 to -0.01); ***only 1 study*** | 4.21 (p=0.12) |
| Total | Adjusted studies vs  Unadjusted studies | *Not possible, as no study reported appropriate adjusted effect estimates for meta-analysis* | |
| Total | Unconverted studies (12) vs  Converted studies (2) | -0.51 (-0.79 to -0.23), 92%  -0.36 (-0.72 to -0.01); 96% | 0.41 (p=0.52) |

# Table S16: Eggers’ test

| **Population** | **p** |
| --- | --- |
| Total physical activity | 0.052 |
| Moderate-to-vigorous physical activity | 0.02 |

# Figure S1: PRISMA Flow Chart


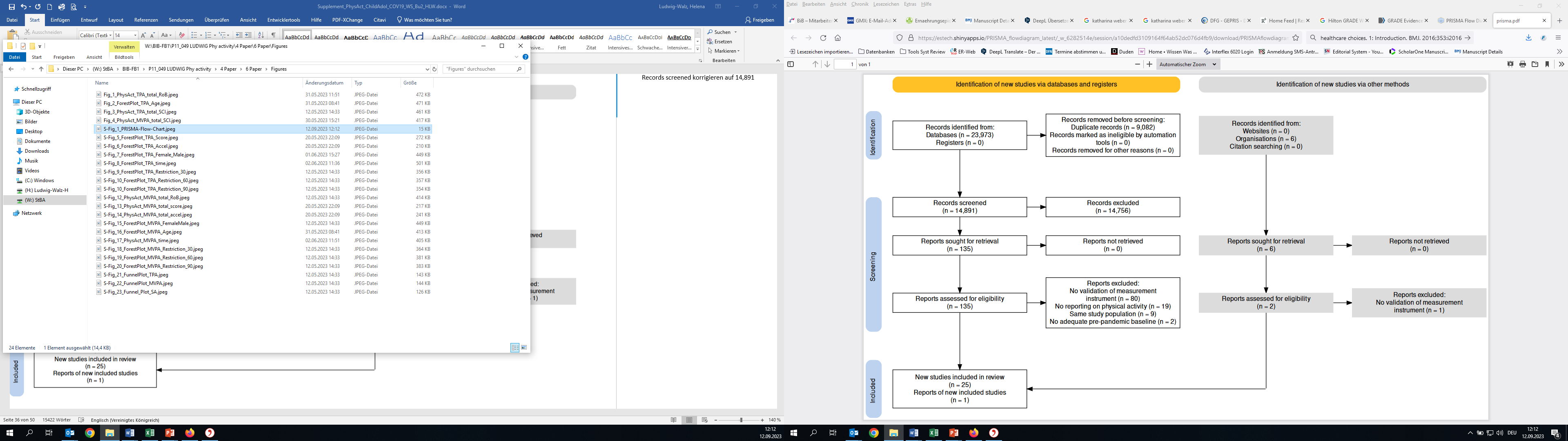


# Figure S2: Graphical distribution of the studies included


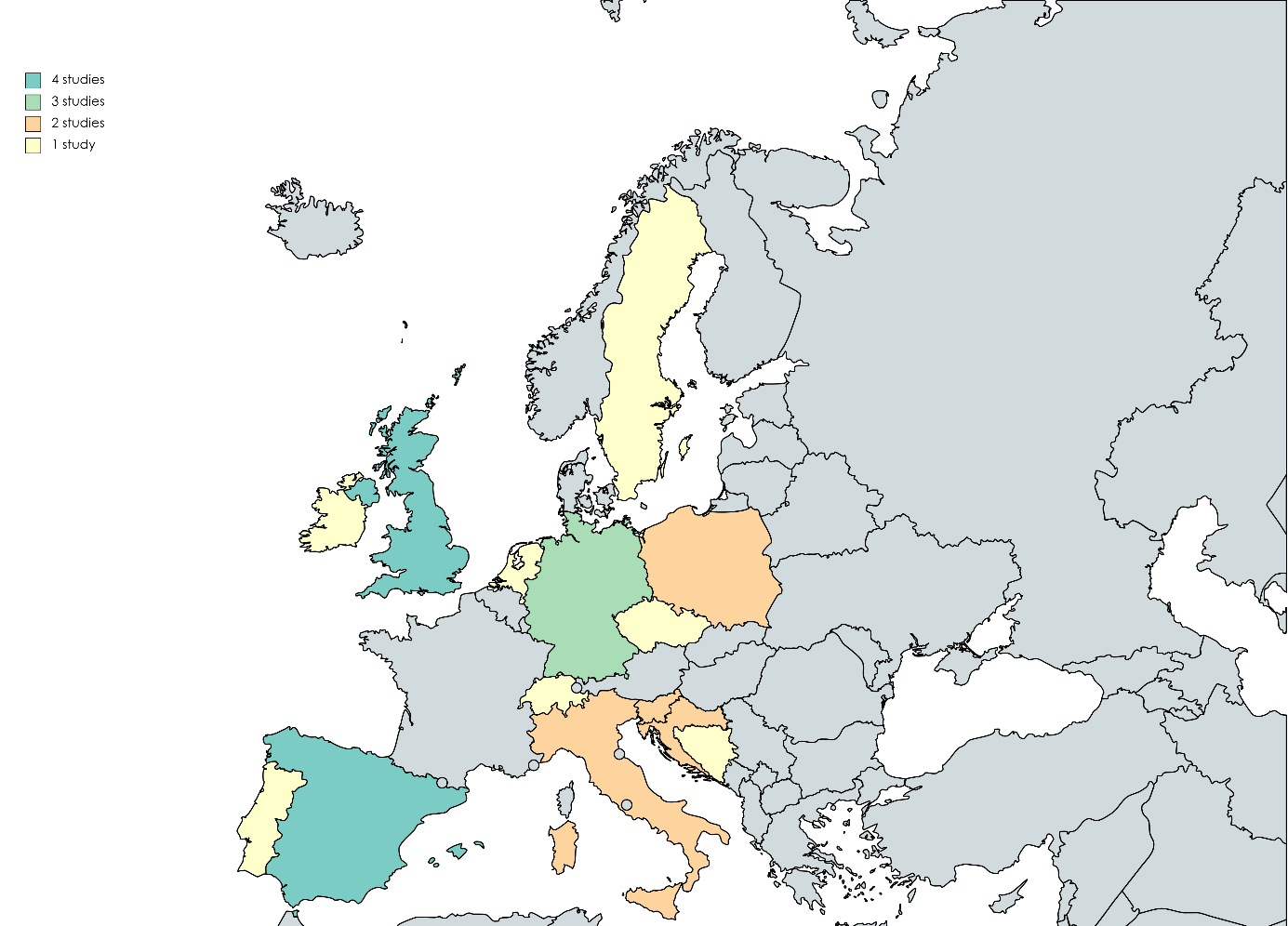


Figure was created using the tool *mapchart.net.*

# Figure S3: Traffic-light plots of the domain-level judgements for each individual result

**
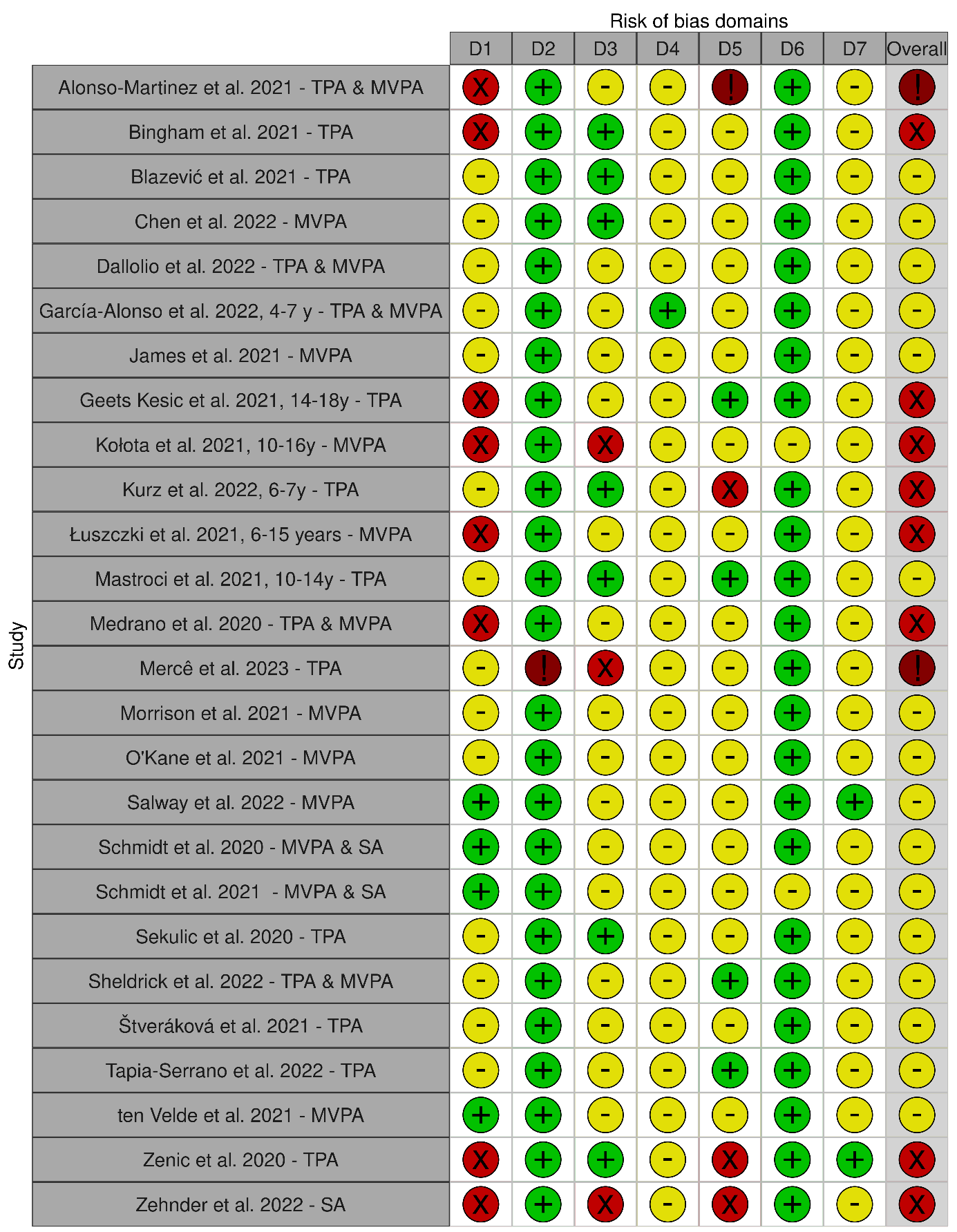
**


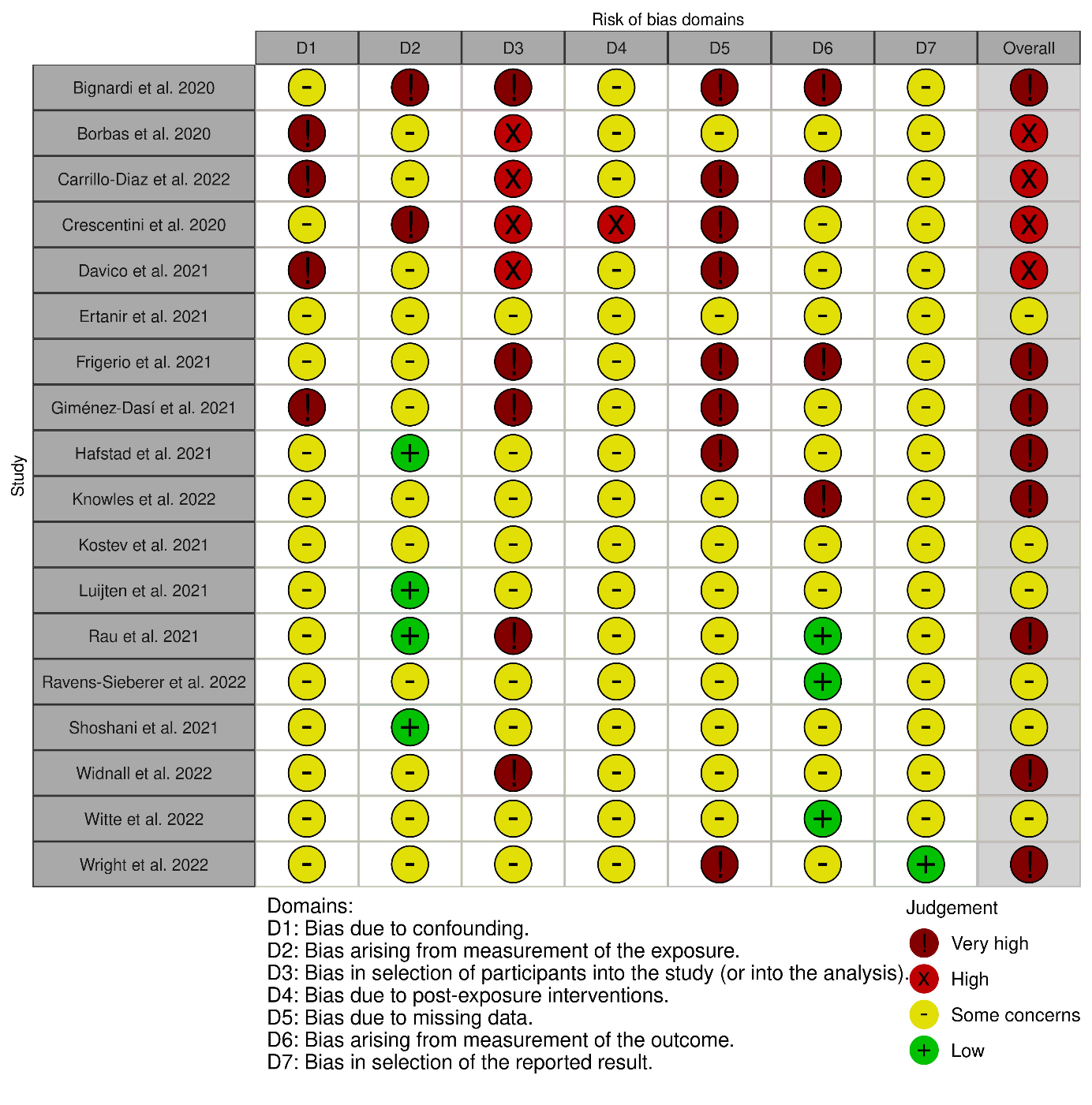


MVPA=moderate-to-vigorous; SA=sports activity; TPA=total physical activity;

Figure was created using the tool *robvis* [157].

# Figure S4: Weighted-bar plots of the distribution of risk of bias judgements within each bias domain


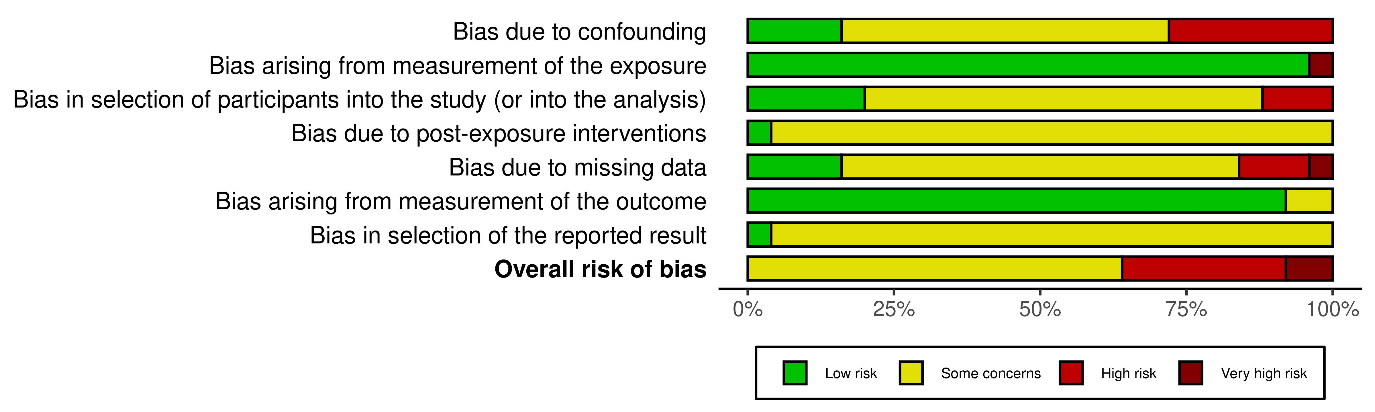


Figure was created using the tool *robvis* [157].

# Figure S5: Forest plot of changes in total physical activity comparing before and during COVID-19 pandemic, using Physical Activity Questionnaire for Children and Adolescents

*Figures S5 to S22 are attached as separate files.*

# Figure S6: Forest plot of changes in total physical activity comparing before and during COVID-19 pandemic, using accelerometer measurements

# Figure S7: Forest plot of changes in female and male total physical activity comparing before and during COVID-19 pandemic

# Figure S8: Forest plot of changes according to time course in total physical activity comparing before and during COVID-19 pandemic

# Figure S9: Forest plot of changes according to a restriction length >30 days before measurement in total physical activity comparing before and during COVID-19 pandemic

# Figure S10: Forest plot of changes according to a restriction length >60 days before measurement in total physical activity comparing before and during COVID-19 pandemic

# Figure S11: Forest plot of changes according to a restriction length >90 days before measurement in total physical activity comparing before and during COVID-19 pandemic

# Figure S12: Forest plot of changes in moderate-to-vigorous physical activity comparing before and during COVID-19 pandemic

# Figure S13: Forest plot of changes in moderate-to-vigorous physical activity comparing before and during COVID-19 pandemic, using self-reported score measurements

# Figure S14: Forest plot of changes in moderate-to-vigorous physical activity comparing before and during COVID-19 pandemic, using accelerometer measurements

# Figure S15: Forest plot of changes in female and male moderate-to-vigorous physical activity comparing before and during COVID-19 pandemic

# Figure S16: Forest plot of changes in moderate-to-vigorous physical activity comparing different age groups

# Figure S17: Forest plot of changes according to time course in moderate-to-vigorous physical activity comparing before and during COVID-19 pandemic

# Figure S18: Forest plot of changes according to a restriction length >30 days before measurement in moderate-to-vigorous physical activity comparing before and during COVID-19 pandemic

# Figure S19: Forest plot of changes according to a restriction length >60 days before measurement in moderate-to-vigorous physical activity comparing before and during COVID-19 pandemic

# Figure S20: Forest plot of changes according to a restriction length >90 days before measurement in moderate-to-vigorous physical activity comparing before and during COVID-19 pandemic

# Figure S21: Funnel plot of changes in total physical activity comparing before and during COVID-19 pandemic

# Figure S22: Funnel plot of changes in moderate-to-vigorous physical activity comparing before and during COVID-19 pandemic

# Figure S23: Funnel plot of changes in sporting activity comparing before and during COVID-19 pandemic

*Figures S5 to S22 are attached as separate files.*

References

1. Centers for Disease Control and Prevention. Child Development. 2021. https://www.cdc.gov/ncbddd/childdevelopment/positiveparenting/index.html. Accessed 30 May 2023.

2. Alvurdu S, Baykal C, Akyildiz Z, Şenel Ö, Silva AF, Conte D, Clemente FM. Impact of Prolonged Absence of Organized Training on Body Composition, Neuromuscular Performance, and Aerobic Capacity: A Study in Youth Male Soccer Players Exposed to COVID-19 Lockdown. Int J Environ Res Public Health 2022. doi:10.3390/ijerph19031148.

3. Amagliani G, Baldelli G, Italiano P, Frisina P, Schiavano GF, Brandi G, Santi M de. Effect of COVID-19 isolation measures on physical activity of children and their parents, and role of the family environment: a cross-sectional study. Ann Ig. 2023;35:159–77. doi:10.7416/ai.2022.2535.

4. Androutsos O, Perperidi M, Georgiou C, Chouliaras G. Lifestyle Changes and Determinants of Children's and Adolescents' Body Weight Increase during the First COVID-19 Lockdown in Greece: The COV-EAT Study. Nutrients 2021. doi:10.3390/nu13030930.

5. Basterfield L, Burn NL, Galna B, Batten H, Goffe L, Karoblyte G, et al. Changes in children's physical fitness, BMI and health-related quality of life after the first 2020 COVID-19 lockdown in England: A longitudinal study. J Sports Sci. 2022;40:1088–96. doi:10.1080/02640414.2022.2047504.

6. Beck F, Schmidt SCE, Woll A, Reimers AK. Family predictors of physical activity change during the COVID-19 lockdown in preschool children in Germany. J Behav Med. 2022:1–13. doi:10.1007/s10865-022-00382-7.

7. Schmidt SCE, Anedda B, Burchartz A, Eichsteller A, Kolb S, Nigg C, et al. Physical activity and screen time of children and adolescents before and during the COVID-19 lockdown in Germany: a natural experiment. Sci Rep. 2020;10:21780. doi:10.1038/s41598-020-78438-4.

8. Béghin L, Thivel D, Baudelet J-B, Deschamps T, Ovigneur H, Vanhelst J. Change in physical fitness due to the COVID-19 pandemic lockdown in French adolescents: a comparison between two independent large samples from Diagnoform battery. Eur J Pediatr. 2022;181:3955–63. doi:10.1007/s00431-022-04610-9.

9. Bothe K, Schabus M, Eigl E-S, Kerbl R, Hoedlmoser K. Self-reported changes in sleep patterns and behavior in children and adolescents during COVID-19. Sci Rep. 2022;12:20412. doi:10.1038/s41598-022-24509-7.

10. Braksiek M, Lindemann U, Pahmeier I. Physical Activity and Stress of Children and Adolescents during the COVID-19 Pandemic in Germany-A Cross-Sectional Study in Rural Areas. Int J Environ Res Public Health 2022. doi:10.3390/ijerph19148274.

11. Bronikowska M, Krzysztoszek J, Łopatka M, Ludwiczak M, Pluta B. Comparison of Physical Activity Levels in Youths before and during a Pandemic Lockdown. Int J Environ Res Public Health 2021. doi:10.3390/ijerph18105139.

12. Brzęk A, Strauss M, Sanchis-Gomar F, Leischik R. Physical Activity, Screen Time, Sedentary and Sleeping Habits of Polish Preschoolers during the COVID-19 Pandemic and WHO's Recommendations: An Observational Cohort Study. Int J Environ Res Public Health 2021. doi:10.3390/ijerph182111173.

13. Burdzovic Andreas J, Brunborg GS. Self-reported Mental and Physical Health Among Norwegian Adolescents Before and During the COVID-19 Pandemic. JAMA Netw Open. 2021;4:e2121934. doi:10.1001/jamanetworkopen.2021.21934.

14. Carballo-Fazanes A, Rodrigues LP, Silva R, Lopes VP, Abelairas-Gómez C. The Developmental Trajectory of Motor Competence of Children That Lived the COVID-19 Confinement Period: A Four-Year Follow-Up Study in Portuguese Children. J Funct Morphol Kinesiol 2022. doi:10.3390/jfmk7030064.

15. Carrillo-Diaz M, Ortega-Martínez AR, Romero-Maroto M, González-Olmo MJ. Lockdown impact on lifestyle and its association with oral parafunctional habits and bruxism in a Spanish adolescent population. Int J Paediatr Dent. 2022;32:185–93. doi:10.1111/ipd.12843.

16. Carvalho S, Sousa B. Effect of the covid-19 pandemic on the nutritional status and eating habits of portuguese children and adolescents. Clinical Nutrition ESPEN. 2021;46:S780. doi:10.1016/j.clnesp.2021.09.671.

17. López-Bueno R, López-Sánchez GF, Casajús JA, Calatayud J, Gil-Salmerón A, Grabovac I, et al. Health-Related Behaviors Among School-Aged Children and Adolescents During the Spanish Covid-19 Confinement. Front Pediatr. 2020;8:573. doi:10.3389/fped.2020.00573.

18. Cassar D, Bonello C, Grixti, KG, Falzon N, Bartolo M. The impact of COVID-19 on fitness behaviour amongst a sample of the Maltese population. Malta Medical Journal. 2021;33:49–59.

19. Censi L, Ruggeri S, Galfo M, Buonocore P, Roccaldo R. Eating behaviour, physical activity and lifestyle of Italian children during lockdown for COVID-19. Int J Food Sci Nutr. 2022;73:93–105. doi:10.1080/09637486.2021.1921127.

20. Chambonnière C, Fearnbach N, Pelissier L, Genin P, Fillon A, Boscaro A, et al. Adverse Collateral Effects of COVID-19 Public Health Restrictions on Physical Fitness and Cognitive Performance in Primary School Children. Int J Environ Res Public Health 2021. doi:10.3390/ijerph182111099.

21. Chambonniere C, Lambert C, Fearnbach N, Tardieu M, Fillon A, Genin P, et al. Effect of the COVID-19 lockdown on physical activity and sedentary behaviors in French children and adolescents: New results from the ONAPS national survey. Eur J Integr Med. 2021;43:101308. doi:10.1016/j.eujim.2021.101308.

22. Cipolla C, Curatola A, Ferretti S, Giugno G, Condemi C, Delogu AB, et al. Eating habits and lifestyle in children with obesity during the COVID19 lockdown: a survey in an Italian center. Acta Biomed. 2021;92:e2021196. doi:10.23750/abm.v92i2.10912.

23. Çoban AE, Kaptan N. Psychological and Behavioral Impacts of the COVID-19 Pandemic on Children and Adolescents in Turkey. Int J Environ Res Public Health 2022. doi:10.3390/ijerph192316207.

24. Corica D, Li Pomi A, Curatola S, Pepe G, Giandalia A, Tropeano A, et al. Impact of Covid-19 Pandemic on the Effectiveness of Outpatient Counseling in Childhood Obesity Management. Front Endocrinol (Lausanne). 2022;13:879440. doi:10.3389/fendo.2022.879440.

25. Dauty M, Menu P, Fouasson-Chailloux A. Effects of the COVID-19 confinement period on physical conditions in young elite soccer players. J Sports Med Phys Fitness. 2021;61:1252–7. doi:10.23736/S0022-4707.20.11669-4.

26. Derigny T, Schnitzler C, Gandrieau J, Potdevin F. Resilience of adolescents in physical activity during the covid-19 pandemic: a preliminary case study in France. Physical Activity Review. 2022;10:86–97.

27. Di Renzo L, Gualtieri P, Pivari F, Soldati L, Attinà A, Cinelli G, et al. Eating habits and lifestyle changes during COVID-19 lockdown: an Italian survey. J Transl Med. 2020;18:229. doi:10.1186/s12967-020-02399-5.

28. Dragun R, Veček NN, Marendić M, Pribisalić A, Đivić G, Cena H, et al. Have Lifestyle Habits and Psychological Well-Being Changed among Adolescents and Medical Students Due to COVID-19 Lockdown in Croatia? Nutrients 2020. doi:10.3390/nu13010097.

29. am Dzielska, Nałȩcz H, Kleszczewska D, Mazur J. Consequences of the COVID-19 pandemic on adolescents' health and health behaviour. Cogent Medicine. 2021;8.

30. Eberhardt T, Bös K, Niessner C. Changes in Physical Fitness during the COVID-19 Pandemic in German Children. Int J Environ Res Public Health 2022. doi:10.3390/ijerph19159504.

31. Eisenburger N, Friesen D, Haas F, Klaudius M, Schmidt L, Vandeven S, Joisten C. Short report: Weight management of children and adolescents with obesity during the COVID-19 pandemic in Germany. PLoS One. 2022;17:e0267601. doi:10.1371/journal.pone.0267601.

32. Farello G, D'Andrea M, Quarta A, Grossi A, Pompili D, Altobelli E, et al. Children and Adolescents Dietary Habits and Lifestyle Changes during COVID-19 Lockdown in Italy. Nutrients 2022. doi:10.3390/nu14102135.

33. Fehr U, Strobl H. Entwicklung der körperlichen Leistungsfähigkeit bei Kindern während der coronabedingten Einschränkungen im Frühjahr/Sommer 2020. [Changes in Children's Physical Fitness during Corona-Related Restrictions in Spring/Summer 2020]. Gesundheitswesen. 2022;84:1006–9. doi:10.1055/a-1657-9863.

34. Fillon A, Lambert C, Tardieu M, Genin P, Larras B, Melsens P, et al. Impact of the COVID-19 confinement on movement behaviors among French young children: the ONAPS national survey. Minerva Pediatr (Torino) 2021. doi:10.23736/S2724-5276.21.06194-2.

35. Francisco R, Pedro M, Delvecchio E, Espada JP, Morales A, Mazzeschi C, Orgilés M. Psychological Symptoms and Behavioral Changes in Children and Adolescents During the Early Phase of COVID-19 Quarantine in Three European Countries. Front Psychiatry. 2020;11:570164. doi:10.3389/fpsyt.2020.570164.

36. Frömel K, Groffik D, Valach P, ŠafáŘ M, MitáŠ J. The Impact of Distance Education during the COVID-19 Pandemic on Physical Activity and Well-Being of Czech and Polish Adolescents. J Sch Health. 2022;92:1137–47. doi:10.1111/josh.13232.

37. Füzéki E, Schröder J, Carraro N, Merlo L, Reer R, Groneberg DA, Banzer W. Physical Activity during the First COVID-19-Related Lockdown in Italy. Int J Environ Res Public Health 2021. doi:10.3390/ijerph18052511.

38. Galhardo J, Dinis I, Limbert C. Glycaemic control in T1D children and adolescents during Portuguese COVID-19 first lockdown: A family's survey report. Pediatric Diabetes. 2021;22(SUPPL 30).

39. Galluccio A, Caparello G, Avolio E, Manes E, Ferraro S, Giordano C, et al. Self-Perceived Physical Activity and Adherence to the Mediterranean Diet in Healthy Adolescents during COVID-19: Findings from the DIMENU Pilot Study. Healthcare (Basel) 2021. doi:10.3390/healthcare9060622.

40. García-Alonso Y, García-Hermoso A, Izquierdo M, Legarra-Gorgoñon G, Ramírez-Vélez R, Alonso-Martínez AM. Relationship between parents' and children's objectively assessed movement behaviours prior to and during the COVID-19 pandemic. Pediatr Obes. 2022;17:e12923. doi:10.1111/ijpo.12923.

41. Genin PM, Lambert C, Larras B, Pereira B, Toussaint J-F, Baker JS, et al. How Did the COVID-19 Confinement Period Affect Our Physical Activity Level and Sedentary Behaviors? Methodology and First Results From the French National ONAPS Survey. J Phys Act Health. 2021;18:296–303. doi:10.1123/jpah.2020-0449.

42. Gentili F, Cafiero G, Perrone MA, Bianco M, Salvati A, Giordano U, et al. The Effects of Physical Inactivity and Exercise at Home in Young Patients with Congenital Heart Disease during the COVID-19 Pandemic. Int J Environ Res Public Health 2021. doi:10.3390/ijerph181910065.

43. Gilic B, Zenic N, Separovic V, Jurcev Savicevic A, Sekulic D. Evidencing the influence of pre-pandemic sports participation and substance misuse on physical activity during the COVID‑19 lockdown: a prospective analysis among older adolescents. Int J Occup Med Environ Health. 2021;34:151–63. doi:10.13075/ijomeh.1896.01733.

44. Geets Kesic M, Gilic B, Cerkez Zovko I, Drid P, Korovljev D, Sekulic D. Differential impact of COVID-19 lockdown on physical activity in younger and older adolescents - prospective study. Med Pr. 2021;72:633–43. doi:10.13075/mp.5893.01180.

45. Gilic B, Ostojic L, Corluka M, Volaric T, Sekulic D. Contextualizing Parental/Familial Influence on Physical Activity in Adolescents before and during COVID-19 Pandemic: A Prospective Analysis. Children (Basel) 2020. doi:10.3390/children7090125.

46. Gobbi E, Maltagliati S, Sarrazin P, Di Fronso S, Colangelo A, Cheval B, et al. Promoting Physical Activity during School Closures Imposed by the First Wave of the COVID-19 Pandemic: Physical Education Teachers' Behaviors in France, Italy and Turkey. Int J Environ Res Public Health 2020. doi:10.3390/ijerph17249431.

47. Gomes L, Martins J, Ramos M, Da Carreiro Costa F. Physical activity levels of Portuguese adolescents in the first period of confinement due to the COVID-19 pandemic and the first activities of teachers and coaches: a cross-sectional study. Retos. 2023;47:701–9. doi:10.47197/retos.v47.93923.

48. Greier K, Drenowatz C, Bischofer T, Petrasch G, Greier C, Cocca A, Ruedl G. Physical activity and sitting time prior to and during COVID-19 lockdown in Austrian high-school students. AIMS Public Health. 2021;8:531–40. doi:10.3934/publichealth.2021043.

49. Hall-López JA, Ochoa-Martínez PY, Alarcón-Meza EI, Teixeira AM. Actividad física evaluada en la clase de educación física en estudiantes de secundaria con discapacidad y sin discapacidad antes y durante la pandemia por COVID-19 (Physical activity assessed in physical education class in disabled and non-disabled high. Retos. 2021;43:447–51. doi:10.47197/retos.v43i0.89497.

50. Heymsfield S, Pecoraro L, Ferruzzi A, Heo M, Faith M, Zoller T, et al. Effects of COVID-19 Lockdown on Lifestyle Behaviors in Children With Obesity: A Longitudinal Study. Special Issue: Abstracts from the 38th Annual Meeting of the Obesity Society at Obesityweek® Interactive November 2–6, 2020. 2020;28:40–187. doi:10.1002/oby.23063.

51. Humer E, Probst T, Wagner-Skacel J, Pieh C. Association of Health Behaviors with Mental Health Problems in More than 7000 Adolescents during COVID-19. Int J Environ Res Public Health 2022. doi:10.3390/ijerph19159072.

52. Iughetti L, Candia F, Stefanelli F, Trevisani V, Bruzzi P, Predieri B. Auxological data and glycemic control in children and adolescents with type 1 diabetes 2-years after the COVID-19 pandemic. Pediatric Diabetes. 2022;23:44–178. doi:10.1111/pedi.13400.

53. Jarnig G, Jaunig J, van Poppel MNM. Association of COVID-19 Mitigation Measures With Changes in Cardiorespiratory Fitness and Body Mass Index Among Children Aged 7 to 10 Years in Austria. JAMA Netw Open. 2021;4:e2121675. doi:10.1001/jamanetworkopen.2021.21675.

54. Jarnig G, Kerbl R, van Poppel MNM. Change in BMI and Fitness among Primary School Children in Austria: A 24-Month Follow-Up Study of 303 Children Measured before and during the Ongoing COVID-19 Pandemic. Sports (Basel) 2022. doi:10.3390/sports10050078.

55. Jarnig G, Kerbl R, van Poppel MNM. The Impact of COVID-19-Related Mitigation Measures on the Health and Fitness Status of Primary School Children in Austria: A Longitudinal Study with Data from 708 Children Measured before and during the Ongoing COVID-19 Pandemic. Sports (Basel) 2022. doi:10.3390/sports10030043.

56. Jarnig G, Kerbl R, van Poppel MN. Changes in Children’s Fitness and Health Status Over the Course of the COVID-19 Pandemic: A 34-month Longitudinal Study of 331 Primary School Children (September 2019 to June 2022); 2022.

57. Jaskulska S, Jankowiak B, Marciniak M, Klichowski M. Assessment of Physical Well-Being and Leisure Time of Polish Students during the COVID-19 Outbreak. Int J Environ Res Public Health 2022. doi:10.3390/ijerph19148358.

58. Jurak G, Morrison SA, Kovač M, Leskošek B, Sember V, Strel J, Starc G. A COVID-19 Crisis in Child Physical Fitness: Creating a Barometric Tool of Public Health Engagement for the Republic of Slovenia. Front Public Health. 2021;9:644235. doi:10.3389/fpubh.2021.644235.

59. Katona ZB, Takács J, Gyömörei T, Soldos P, Ihász F. A fizikai aktivitás és a szubjektív egészségi állapot értékelése magyar középiskolások körében a COVID-19-pandémia okán elrendelt távoktatási időszakban. [Assessing physical activity and subjective health status among Hungarian secondary school students during the distance learning period caused by the COVID-19 pandemic]. Orv Hetil. 2022;163:655–62. doi:10.1556/650.2022.32481.

60. Katona ZB, Takács J, Kerner L, Alföldi Z, Soós I, Gyömörei T, et al. Physical Activity and Screen Time among Hungarian High School Students during the COVID-19 Pandemic Caused Distance Education Period. Int J Environ Res Public Health 2021. doi:10.3390/ijerph182413024.

61. Kuygun Karci C, Arici Gurbuz A. Challenges of children and adolescents with attention-deficit/hyperactivity disorder during the COVID-19 pandemic. Nord J Psychiatry. 2022;76:372–9. doi:10.1080/08039488.2021.1980610.

62. Kołota A, Głąbska D. Analysis of Food Habits during Pandemic in a Polish Population-Based Sample of Primary School Adolescents: Diet and Activity of Youth during COVID-19 (DAY-19) Study. Nutrients 2021. doi:10.3390/nu13113711.

63. Kołota A, Głąbska D. Analysis of Association between Adolescents' Food Habits and Body Mass Change in a Population-Based Sample: Diet and Activity of Youth during COVID-19 (DAY-19) Study. Int J Environ Res Public Health 2022. doi:10.3390/ijerph191811772.

64. Konstantinou C, Andrianou XD, Constantinou A, Perikkou A, Markidou E, Christophi CA, Makris KC. Exposome changes in primary school children following the wide population non-pharmacological interventions implemented due to COVID-19 in Cyprus: A national survey. EClinicalMedicine. 2021;32:100721. doi:10.1016/j.eclinm.2021.100721.

65. López-Bueno R, Calatayud J, Andersen LL, Casaña J, Ezzatvar Y, Casajús JA, et al. Cardiorespiratory fitness in adolescents before and after the COVID-19 confinement: a prospective cohort study. Eur J Pediatr. 2021;180:2287–93. doi:10.1007/s00431-021-04029-8.

66. López-Gil JF, Tremblay MS, Brazo-Sayavera J. Changes in Healthy Behaviors and Meeting 24-h Movement Guidelines in Spanish and Brazilian Preschoolers, Children and Adolescents during the COVID-19 Lockdown. Children (Basel) 2021. doi:10.3390/children8020083.

67. Martinko A, Starc G, Jurak G, Soric M. Changes in physical fitness during and in the aftermath of COVID-19 restrictions among children in Slovenia: is there evidence for weight-based inequalities? Obesity Reviews 2022. doi:10.1111/obr.13502.

68. Maugeri G, Castrogiovanni P, Battaglia G, Pippi R, D'Agata V, Palma A, et al. The impact of physical activity on psychological health during Covid-19 pandemic in Italy. Heliyon. 2020;6:e04315. doi:10.1016/j.heliyon.2020.e04315.

69. Medina Cantillo J, Moya O, Rosello M, Roca S, Leon D, Claramunt M, et al. Effects of confinement in Spain during the COVID pandemic on the Duchenne muscular dystrophy pediatric population: The importance of daily physical activity. Develop Med Child Neuro. 2022;64:54–100. doi:10.1111/dmcn.15215.

70. Minuto N, Bassi M, Montobbio C, Vinci F, Mercuri C, Perri FN, et al. The Effect of Lockdown and Physical Activity on Glycemic Control in Italian Children and Young Patients With Type 1 Diabetes. Front Endocrinol (Lausanne). 2021;12:690222. doi:10.3389/fendo.2021.690222.

71. Morgül E, Kallitsoglou A, Essau CA. Psychological effects of the COVID-19 lockdown on children and families in the UK. rpcna. 2020;7:42–8. doi:10.21134/rpcna.2020.mon.2049.

72. Ng K, Cooper J, McHale F, Clifford J, Woods C. Barriers and facilitators to changes in adolescent physical activity during COVID-19. BMJ Open Sport Exerc Med. 2020;6:e000919. doi:10.1136/bmjsem-2020-000919.

73. Nigg C, Oriwol D, Wunsch K, Burchartz A, Kolb S, Worth A, et al. Population density predicts youth's physical activity changes during Covid-19 - Results from the MoMo study. Health Place. 2021;70:102619. doi:10.1016/j.healthplace.2021.102619.

74. Nyberg G, Kjellenberg K, Helgadóttir B, Ekblom Ö. P05-10 Changes in mental health and physical activity patterns before and during the covid-19 pandemic in Swedish adolescents - a longitudinal study. European Journal of Public Health 2022. doi:10.1093/eurpub/ckac095.077.

75. Delisle Nyström C, Alexandrou C, Henström M, Nilsson E, Okely AD, Wehbe El Masri S, Löf M. International Study of Movement Behaviors in the Early Years (SUNRISE): Results from SUNRISE Sweden's Pilot and COVID-19 Study. Int J Environ Res Public Health 2020. doi:10.3390/ijerph17228491.

76. Okely AD, Kariippanon KE, Guan H, Taylor EK, Suesse T, Cross PL, et al. Global effect of COVID-19 pandemic on physical activity, sedentary behaviour and sleep among 3- to 5-year-old children: a longitudinal study of 14 countries. BMC Public Health. 2021;21:940. doi:10.1186/s12889-021-10852-3.

77. Orgilés M, Morales A, Delvecchio E, Mazzeschi C, Espada JP. Immediate Psychological Effects of the COVID-19 Quarantine in Youth From Italy and Spain. Front Psychol. 2020;11:579038. doi:10.3389/fpsyg.2020.579038.

78. Pajek SV. Impact of the COVID-19 Pandemic on the Motor Development of Schoolchildren in Rural and Urban Environments. Biomed Res Int. 2022;2022:8937693. doi:10.1155/2022/8937693.

79. Palermi S, Vecchiato M, Pennella S, Marasca A, Spinelli A, Luca M de, et al. The Impact of the COVID-19 Pandemic on Childhood Obesity and Lifestyle-A Report from Italy. Pediatr Rep. 2022;14:410–8. doi:10.3390/pediatric14040049.

80. Peralta GP, Camerini A-L, Haile SR, Kahlert CR, Lorthe E, Marciano L, et al. Lifestyle Behaviours of Children and Adolescents During the First Two Waves of the COVID-19 Pandemic in Switzerland and Their Relation to Well-Being: An Observational Study. Int J Public Health. 2022;67:1604978. doi:10.3389/ijph.2022.1604978.

81. Pietrobelli A, Pecoraro L, Ferruzzi A, Heo M, Faith M, Zoller T, et al. Effects of COVID-19 Lockdown on Lifestyle Behaviors in Children with Obesity Living in Verona, Italy: A Longitudinal Study. Obesity. 2020;28:1382–5. doi:10.1002/oby.22861.

82. Pietrobelli A, Fearnbach N, Ferruzzi A, Vrech M, Heo M, Faith M, et al. Effects of COVID-19 lockdown on lifestyle behaviors in children with obesity: Longitudinal study update. Obes Sci Pract. 2022;8:525–8. doi:10.1002/osp4.581.

83. Planinšec J, Matejek Č, Pišot S, Pišot R, Šimunič B. Consequences of COVID-19 Lockdown Restrictions on Children Physical Activity-A Slovenian Study. Front Public Health. 2022;10:843448. doi:10.3389/fpubh.2022.843448.

84. Pombo A, Luz C, Rodrigues LP, Cordovil R. Effects of COVID-19 Confinement on the Household Routines Of Children in Portugal. J Child Fam Stud. 2021;30:1664–74. doi:10.1007/s10826-021-01961-z.

85. Pombo A, Luz C, Sá C de, Rodrigues LP, Cordovil R. Effects of the COVID-19 Lockdown on Portuguese Children's Motor Competence. Children (Basel) 2021. doi:10.3390/children8030199.

86. Pucsok JM, Kovács M, Ráthonyi G, Pocsai B, Balogh L. The Impact of COVID-19 Lockdown on Agility, Explosive Power, and Speed-Endurance Capacity in Youth Soccer Players. Int J Environ Res Public Health 2021. doi:10.3390/ijerph18189604.

87. Pyšná J, Pyšný L, Cihlář D, Petrů D, Müllerová LH, Čtvrtečka L, et al. Physical Activity and BMI before and after the Situation Caused by COVID-19 in Upper Primary School Pupils in the Czech Republic. Int J Environ Res Public Health 2022. doi:10.3390/ijerph19053068.

88. Ramos Álvarez O, Arufe Giráldez V, Cantarero Prieto D, Ibáñez García A. Changes in Physical Fitness, Dietary Habits and Family Habits for Spanish Children during SARS-CoV-2 Lockdown. Int J Environ Res Public Health 2021. doi:10.3390/ijerph182413293.

89. Rúa-Alonso M, Rial-Vázquez J, Nine I, Lete-Lasa JR, Clavel I, Giráldez-García MA, et al. Comparison of Physical Fitness Profiles Obtained before and during COVID-19 Pandemic in Two Independent Large Samples of Children and Adolescents: DAFIS Project. Int J Environ Res Public Health 2022. doi:10.3390/ijerph19073963.

90. Rucińska M, Rutkowska N, Skowronek M, Matusik P, Zachurzok A. Desirable and undesirable lifestyle changes in Polish children resulting from the COVID-19 pandemic. polp. 2022;97:213–20. doi:10.5114/polp.2022.120196.

91. Sánchez-Sánchez E, Ramírez-Vargas G, Avellaneda-López Y, Orellana-Pecino JI, García-Marín E, Díaz-Jimenez J. Eating Habits and Physical Activity of the Spanish Population during the COVID-19 Pandemic Period. Nutrients 2020. doi:10.3390/nu12092826.

92. Schmidt T, Pawlowski CS. Physical Activity in Crisis: The Impact of COVID-19 on Danes' Physical Activity Behavior. Front Sports Act Living. 2020;2:610255. doi:10.3389/fspor.2020.610255.

93. Sekulic D, Ostojic D, Decelis A, Castro-Piñero J, Jezdimirovic T, Drid P, et al. The Impact of Scholastic Factors on Physical Activity Levels during the COVID-19 Lockdown: A Prospective Study on Adolescents from Bosnia and Herzegovina. Children (Basel) 2021. doi:10.3390/children8100877.

94. Shneor E, Doron R, Levine J, Zimmerman DR, Benoit JS, Ostrin LA, Gordon-Shaag A. Objective Behavioral Measures in Children before, during, and after the COVID-19 Lockdown in Israel. Int J Environ Res Public Health 2021. doi:10.3390/ijerph18168732.

95. Siachpazidou DI, Kotsiou OS, Chatziparasidis G, Papagiannis D, Vavougios GD, Gogou E, et al. Action and Reaction of Pre-Primary and Primary School-Age Children to Restrictions during COVID-19 Pandemic in Greece. J Pers Med 2021. doi:10.3390/jpm11060451.

96. Sport England. Active Lives Children and Young People Survey: Academic year 2021-22; 2022.

97. Sunda M, Gilic B, Peric I, Jurcev Savicevic A, Sekulic D. Evidencing the Influence of the COVID-19 Pandemic and Imposed Lockdown Measures on Fitness Status in Adolescents: A Preliminary Report. Healthcare (Basel) 2021. doi:10.3390/healthcare9060681.

98. Szabó T, Stocker M, Ács P, Morvay-Sey K, Pálvölgyi Á, Laczkó T. Impact of COVID-19 on the physical activity and well-being of hungarian athletes and sports professionals. hpc. 2020;14:165–73. doi:10.5114/hpc.2020.98471.

99. Szczepańska E, Janota B. Lifestyle of Families with Children Aged 4-8 Years before and during Lockdown Due to COVID-19 Pandemic in Poland. Int J Environ Res Public Health 2022. doi:10.3390/ijerph192215064.

100. Tatlibal P, Oral O, Aydin I. Vitamin, Mineral Use and Participation in Exercise during the Coronavirus-19 Pandemic Period. PJMHS. 2021;15:2957–60. doi:10.53350/pjmhs2115102957.

101. Theis N, Campbell N, Leeuw J de, Owen M, Schenke KC. The effects of COVID-19 restrictions on physical activity and mental health of children and young adults with physical and/or intellectual disabilities. Disabil Health J. 2021;14:101064. doi:10.1016/j.dhjo.2021.101064.

102. Thomas J, Bowes N, Meyers R, Thirlaway K. Mental well-being and physical activity of young people experiencing homelessness before and during COVID-19 lockdown: A longitudinal study. Ment Health Phys Act. 2021;21:100407. doi:10.1016/j.mhpa.2021.100407.

103. Tornaghi M, Lovecchio N, Vandoni M, Chirico A, Codella R. Physical activity levels across COVID-19 outbreak in youngsters of Northwestern Lombardy. J Sports Med Phys Fitness. 2021;61:971–6. doi:10.23736/S0022-4707.20.11600-1.

104. Tsoukos A, Bogdanis GC. The Effects of a Five-Month Lockdown Due to COVID-19 on Physical Fitness Parameters in Adolescent Students: A Comparison between Cohorts. Int J Environ Res Public Health 2021. doi:10.3390/ijerph19010326.

105. Turan H, Güneş Kaya D, Tarçın G, Evliyaoğlu SO. Effect of the COVID-19 quarantine on metabolic control in children and adolescents with type 1 diabetes. Endocrinol Diabetes Nutr (Engl Ed). 2021;69:201–8. doi:10.1016/j.endinu.2021.05.003.

106. Ventura PS, Ortigoza AF, Castillo Y, Bosch Z, Casals S, Girbau C, et al. Children's Health Habits and COVID-19 Lockdown in Catalonia: Implications for Obesity and Non-Communicable Diseases. Nutrients 2021. doi:10.3390/nu13051657.

107. Vuković J, Matić RM, Milovanović IM, Maksimović N, Krivokapić D, Pišot S. Children's Daily Routine Response to COVID-19 Emergency Measures in Serbia. Front Pediatr. 2021;9:656813. doi:10.3389/fped.2021.656813.

108. Welling MS, Abawi O, van den Eynde E, van Rossum EFC, Halberstadt J, Brandsma AE, et al. Impact of the COVID-19 Pandemic and Related Lockdown Measures on Lifestyle Behaviors and Well-Being in Children and Adolescents with Severe Obesity. Obes Facts. 2022;15:186–96. doi:10.1159/000520718.

109. Wessely S, Ferrari N, Friesen D, Grauduszus M, Klaudius M, Joisten C. Changes in Motor Performance and BMI of Primary School Children over Time-Influence of the COVID-19 Confinement and Social Burden. Int J Environ Res Public Health 2022. doi:10.3390/ijerph19084565.

110. Wunsch K, Nigg C, Niessner C, Schmidt SCE, Oriwol D, Hanssen-Doose A, et al. The Impact of COVID-19 on the Interrelation of Physical Activity, Screen Time and Health-Related Quality of Life in Children and Adolescents in Germany: Results of the Motorik-Modul Study. Children (Basel) 2021. doi:10.3390/children8020098.

111. Zachurzok A, Wójcik M, Gawlik A, Starzyk J, Mazur A. Restriction of physical activity is the main cause of childhood obesity during the COVID-19 pandemic - reflections from a study conducted in 3 clinical centers in southern Poland. 59th Annual Meeting of the European Society for Paediatric Endocrinology. 2021;94.

112. Zachurzok A, Wójcik M, Gawlik A, Starzyk JB, Mazur A. An Attempt to Assess the Impact of Pandemic Restrictions on the Lifestyle, Diet, and Body Mass Index of Children with Endocrine Diseases-Preliminary Results. Nutrients 2022. doi:10.3390/nu14010156.

113. Zorcec T, Jakovska T, Micevska V, Boskovska K, Cholakovska VC. Pandemic with COVID-19 and Families with Children with Chronic Respiratory Diseases. Pril (Makedon Akad Nauk Umet Odd Med Nauki). 2020;41:95–101. doi:10.2478/prilozi-2020-0038.

114. Sheldrick MPR, Swindell NJ, Richards AB, Fairclough SJ, Stratton G. Homes became the "everything space" during COVID-19: impact of changes to the home environment on children's physical activity and sitting. Int J Behav Nutr Phys Act. 2022;19:134. doi:10.1186/s12966-022-01346-5.

115. National Center for Chronic Disease Prevention and Health Promotion. Do Your Children Get Enough Sleep? 2021. https://www.cdc.gov/chronicdisease/resources/infographic/children-sleep.htm. Accessed 25 Apr 2023.

116. Salway R, Foster C, Vocht F de, Tibbitts B, Emm-Collison L, House D, et al. Accelerometer-measured physical activity and sedentary time among children and their parents in the UK before and after COVID-19 lockdowns: a natural experiment. Int J Behav Nutr Phys Act. 2022;19:51. doi:10.1186/s12966-022-01290-4.

117. Mercê C, Cordeiro J, Romão C, Branco M, Catela D. Levels of Physical Activity in Portuguese Children: the Impact of the Covid-19 Pandemic. Retos. 2022;47:174–80. doi:10.47197/retos.v47.94936.

118. Schmidt SCE, Burchartz A, Kolb S, Niessner C, Oriwol D, Hanssen-Doose A, et al. Zur Situation der körperlich-sportlichen Aktivität von Kindern und Jugendlichen während der COVID-19 Pandemie in Deutschland: Die Motorik-Modul Studie (MoMo). 165th ed.; 2021.

119. Kurz D, Braig S, Genuneit J, Rothenbacher D. Lifestyle changes, mental health, and health-related quality of life in children aged 6-7 years before and during the COVID-19 pandemic in South Germany. Child Adolesc Psychiatry Ment Health. 2022;16:20. doi:10.1186/s13034-022-00454-1.

120. Higgins JP, Li T, Deeks JJ. Chapter 6: Choosing effect measures and computing estimates of effect. In: Cochrane Handbook for Systematic Reviews of Interventions. 6th ed.; 2022.

121. Schünemann H, Brożek J, Guyatt G, Oxman A. GRADE Handbook: Handbook for grading the quality of evidence and the strength of recommendations using the GRADE approach. Updated October 2013. https://gdt.gradepro.org/app/handbook/handbook.html#h.w6r7mtvq3mjz. Accessed 31 Aug 2022.

122. Schünemann HJ, Cuello C, Akl EA, Mustafa RA, Meerpohl JJ, Thayer K, et al. GRADE guidelines: 18. How ROBINS-I and other tools to assess risk of bias in nonrandomized studies should be used to rate the certainty of a body of evidence. J Clin Epidemiol. 2019;111:105–14. doi:10.1016/j.jclinepi.2018.01.012.

123. Morgan RL, Thayer KA, Santesso N, Holloway AC, Blain R, Eftim SE, et al. A risk of bias instrument for non-randomized studies of exposures: A users' guide to its application in the context of GRADE. Environ Int. 2019;122:168–84. doi:10.1016/j.envint.2018.11.004.

124. Janz KF, Lutuchy EM, Wenthe P, Levy SM. Measuring activity in children and adolescents using self-report: PAQ-C and PAQ-A. Med Sci Sports Exerc. 2008;40:767–72. doi:10.1249/MSS.0b013e3181620ed1.

125. Kowalski KC, Crocker PR, Kowalski NP. Convergent Validity of the Physical Activity Questionnaire for Adolescents. Pediatric Exercise Science. 1997;9:342–52. doi:10.1123/pes.9.4.342.

126. Sekulic D, Blazevic M, Gilic B, Kvesic I, Zenic N. Prospective Analysis of Levels and Correlates of Physical Activity during COVID-19 Pandemic and Imposed Rules of Social Distancing; Gender Specific Study among Adolescents from Southern Croatia. Sustainability. 2020;12:4072. doi:10.3390/su12104072.

127. Zenic N, Taiar R, Gilic B, Blazevic M, Maric D, Pojskic H, Sekulic D. Levels and Changes of Physical Activity in Adolescents during the COVID-19 Pandemic: Contextualizing Urban vs. Rural Living Environment. Applied Sciences. 2020;10:3997. doi:10.3390/app10113997.

128. O'Kane SM, Lahart IM, Gallagher AM, Carlin A, Faulkner M, Jago R, Murphy MH. Changes in Physical Activity, Sleep, Mental Health, and Social Media Use During COVID-19 Lockdown Among Adolescent Girls: A Mixed-Methods Study. J Phys Act Health. 2021;18:677–85. doi:10.1123/jpah.2020-0649.

129. Štveráková T, Jačisko J, Busch A, Šafářová M, Kolář P, Kobesová A. The impact of COVID-19 on Physical Activity of Czech children. PLoS One. 2021;16:e0254244. doi:10.1371/journal.pone.0254244.

130. Cuberek R, Janíková M, Dygrýn J. Adaptation and validation of the Physical Activity Questionnaire for Older Children (PAQ-C) among Czech children. PLoS One. 2021;16:e0245256. doi:10.1371/journal.pone.0245256.

131. Bayer O, Jarczok M, Fischer J, Kries R von, Bock F de. Validation and extension of a simple questionnaire to assess physical activity in pre-school children. Public Health Nutr. 2012;15:1611–9. doi:10.1017/S1368980012001243.

132. Jekauc D, Wagner MO, Kahlert D, Woll A. Reliabilität und Validität des MoMo-Aktivitätsfragebogens für Jugendliche (MoMo-AFB). Diagnostica. 2013;59:100–11. doi:10.1026/0012-1924/a000083.

133. Hardie Murphy M, Rowe DA, Belton S, Woods CB. Validity of a two-item physical activity questionnaire for assessing attainment of physical activity guidelines in youth. BMC Public Health. 2015;15:1080. doi:10.1186/s12889-015-2418-6.

134. Mastorci F, Piaggi P, Doveri C, Trivellini G, Casu A, Pozzi M, et al. Health-Related Quality of Life in Italian Adolescents During Covid-19 Outbreak. Front Pediatr. 2021;9:611136. doi:10.3389/fped.2021.611136.

135. Dallolio L, Marini S, Masini A, Toselli S, Stagni R, Bisi MC, et al. The impact of COVID-19 on physical activity behaviour in Italian primary school children: a comparison before and during pandemic considering gender differences. BMC Public Health. 2022;22:52. doi:10.1186/s12889-021-12483-0.

136. Pate RR, Almeida MJ, McIver KL, Pfeiffer KA, Dowda M. Validation and calibration of an accelerometer in preschool children. Obesity (Silver Spring). 2006;14:2000–6. doi:10.1038/oby.2006.234.

137. Delisle Nyström C, Pomeroy J, Henriksson P, Forsum E, Ortega FB, Maddison R, et al. Evaluation of the wrist-worn ActiGraph wGT3x-BT for estimating activity energy expenditure in preschool children. Eur J Clin Nutr. 2017;71:1212–7. doi:10.1038/ejcn.2017.114.

138. Hänggi JM, Phillips LRS, Rowlands AV. Validation of the GT3X ActiGraph in children and comparison with the GT1M ActiGraph. J Sci Med Sport. 2013;16:40–4. doi:10.1016/j.jsams.2012.05.012.

139. Velde G ten, Lubrecht J, Arayess L, van Loo C, Hesselink M, Reijnders D, Vreugdenhil A. Physical activity behaviour and screen time in Dutch children during the COVID-19 pandemic: Pre-, during- and post-school closures. Pediatr Obes. 2021;16:e12779. doi:10.1111/ijpo.12779.

140. Prochaska JJ, Sallis JF, Long B. A physical activity screening measure for use with adolescents in primary care. Arch Pediatr Adolesc Med. 2001;155:554–9. doi:10.1001/archpedi.155.5.554.

141. Łuszczki E, Bartosiewicz A, Pezdan-Śliż I, Kuchciak M, Jagielski P, Oleksy Ł, et al. Children's Eating Habits, Physical Activity, Sleep, and Media Usage before and during COVID-19 Pandemic in Poland. Nutrients 2021. doi:10.3390/nu13072447.

142. Morera-Castro M, Jiménez-Díaz J, Araya-Vargas G, Herrera-González E. Cuestionario Pictórico de la Actividad Física Infantil: diseño y validación. Act. Inv. en Educ. 2018. doi:10.15517/aie.v18i2.33127.

143. Blazević M, Gilic B, Peric I, Sekulic D. Physical activity before and during COVID-19 pandemic; Analysis of changes and correlates in croatian adolescents. KINSI. 2021;27:5–17. doi:10.52165/kinsi.27.2.5-17.

144. Morrison SA, Meh K, Sember V, Starc G, Jurak G. The Effect of Pandemic Movement Restriction Policies on Children's Physical Fitness, Activity, Screen Time, and Sleep. Front Public Health. 2021;9:785679. doi:10.3389/fpubh.2021.785679.

145. Adamo KB, Prince SA, Tricco AC, Connor-Gorber S, Tremblay M. A comparison of indirect versus direct measures for assessing physical activity in the pediatric population: a systematic review. Int J Pediatr Obes. 2009;4:2–27. doi:10.1080/17477160802315010.

146. Alonso-Martínez AM, Ramírez-Vélez R, García-Alonso Y, Izquierdo M, García-Hermoso A. Physical Activity, Sedentary Behavior, Sleep and Self-Regulation in Spanish Preschoolers during the COVID-19 Lockdown. Int J Environ Res Public Health 2021. doi:10.3390/ijerph18020693.

147. Duncan MJ, Wilson S, Tallis J, Eyre E. Validation of the Phillips et al. GENEActiv accelerometer wrist cut-points in children aged 5-8 years old. Eur J Pediatr. 2016;175:2019–21. doi:10.1007/s00431-016-2795-6.

148. Adolph AL, Puyau MR, Vohra FA, Nicklas TA, Zakeri IF, Butte NF. Validation of uniaxial and triaxial accelerometers for the assessment of physical activity in preschool children. J Phys Act Health. 2012;9:944–53. doi:10.1123/jpah.9.7.944.

149. Medrano M, Cadenas-Sanchez C, Oses M, Arenaza L, Amasene M, Labayen I. Changes in lifestyle behaviours during the COVID-19 confinement in Spanish children: A longitudinal analysis from the MUGI project. Pediatr Obes. 2021;16:e12731. doi:10.1111/ijpo.12731.

150. Tapia-Serrano MA, Sánchez-Oliva D, Sevil-Serrano J, Marques A, Sánchez-Miguel PA. 24-h movement behaviours in Spanish youth before and after 1-year into the covid-19 pandemic and its relationship to academic performance. Sci Rep. 2022;12:16660. doi:10.1038/s41598-022-21096-5.

151. Booth ML, Okely AD, Chey T, Bauman A. The reliability and validity of the physical activity questions in the WHO health behaviour in schoolchildren (HBSC) survey: a population study. Br J Sports Med. 2001;35:263–7. doi:10.1136/bjsm.35.4.263.

152. Fuchs R, Klaperski S, Gerber M, Seelig H. Messung der Bewegungs- und Sportaktivität mit dem BSA-Fragebogen. Zeitschrift für Gesundheitspsychologie. 2015;23:60–76. doi:10.1026/0943-8149/a000137.

153. Bingham DD, Daly-Smith A, Hall J, Seims A, Dogra SA, Fairclough SJ, et al. Covid-19 lockdown: Ethnic differences in children's self-reported physical activity and the importance of leaving the home environment; a longitudinal and cross-sectional study from the Born in Bradford birth cohort study. Int J Behav Nutr Phys Act. 2021;18:117. doi:10.1186/s12966-021-01183-y.

154. Fairclough SJ, Christian DL, Saint-Maurice PF, Hibbing PR, Noonan RJ, Welk GJ, et al. Calibration and Validation of the Youth Activity Profile as a Physical Activity and Sedentary Behaviour Surveillance Tool for English Youth. Int J Environ Res Public Health 2019. doi:10.3390/ijerph16193711.

155. James M, Marchant E, Defeyter MA, Woodside J, Brophy S. Impact of school closures on the health and well-being of primary school children in Wales UK: a routine data linkage study using the HAPPEN Survey (2018-2020). BMJ Open. 2021;11:e051574. doi:10.1136/bmjopen-2021-051574.

156. Mora-Gonzalez J, Gould ZR, Moore CC, Aguiar EJ, Ducharme SW, Schuna JM, et al. A catalog of validity indices for step counting wearable technologies during treadmill walking: the CADENCE-adults study. Int J Behav Nutr Phys Act. 2022;19:117. doi:10.1186/s12966-022-01350-9.

157. McGuinness LA, Higgins JPT. Risk-of-bias VISualization (robvis): An R package and Shiny web app for visualizing risk-of-bias assessments. Res Synth Methods. 2021;12:55–61. doi:10.1002/jrsm.1411.
