# Supplementary figures and images for: How the COVID-19 pandemic and related school closures reduce physical activity among children and adolescents in the WHO European Region: a systematic review and meta-analysis
Source: Int J Behav Nutr Phys Act. 2023 Dec 19;20:149. doi: 10.1186/s12966-023-01542-x (PMC10731871; doi:10.1186/s12966-023-01542-x)

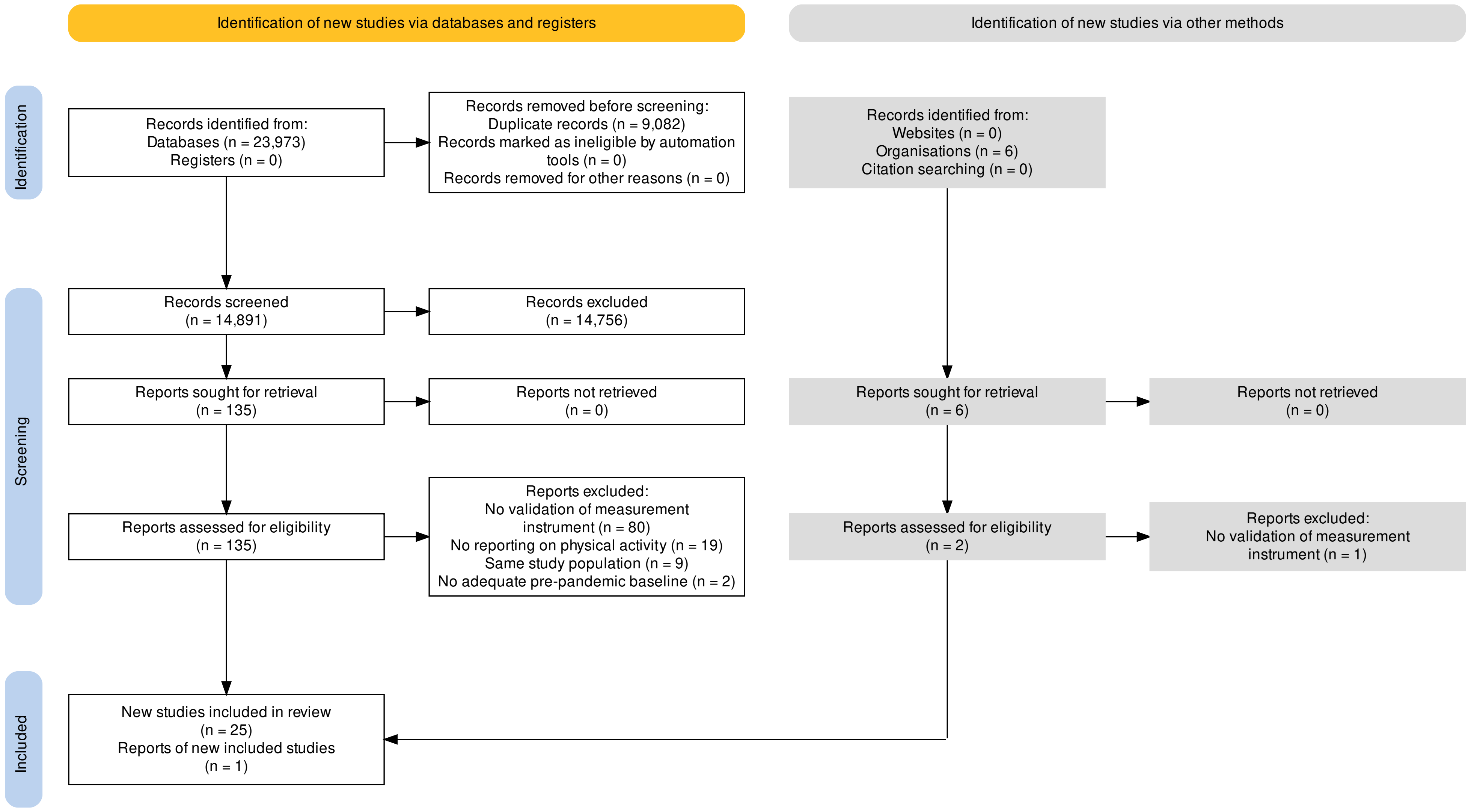

Supplement: Supplementary file 1 — Additional file 1: Table S1. PRISMA item checklist for systematic reviews. Table S2. Deviations from the systematic review protocol. Table S3. Searched websites of key organizations. Table S4. Search strategy. Table S5. Reasons for exclusion of studies from the systematic literature search, after full-text screening. Table S6. Data conversion. Table S7. Criteria for grading evidence according to Grading of Recommendations, Assessment, Development and Evaluations (GRADE). Table S8. Evidence profile for grading evidence according to Grading of Recommendations, Assessment, Development and Evaluations (GRADE). Table S9. Summary of effect estimates. Table S10. Meta-regression for total physical activity with categorical moderators. Table S11. Meta-regression for total physical activity with continuous moderators. Table S12. Meta-regression for moderate-to-vigorous physical activity with categorical moderators. Table S13. Meta-regression for moderate-to-vigorous physical activity with continuous moderators. Table S14. Sensitivity analysis for total physical activity. Table S15. Sensitivity analysis for moderate-to-vigorous physical activity. Table S16. Eggers’ test. Figure S1. PRISMA Flow Chart. Figure S2. Graphical distribution of the studies included. Figure S3. Traffic-light plots of the domain-level judgements for each individual result. Figure S4. Weighted-bar plots of the distribution of risk of bias judgements within each bias domain. Figure S5. Forest plot of changes in total physical activity comparing before and during COVID-19 pandemic, using Physical Activity Questionnaire for Children and Adolescents. Figure S6. Forest plot of changes in total physical activity comparing before and during COVID-19 pandemic, using accelerometer measurements. Figure S7. Forest plot of changes in female and male total physical activity comparing before and during COVID-19 pandemic. Figure S8. Forest plot of changes according to time course in total physical activity comparing be [file 12966_2023_1542_MOESM1_ESM.zip › S-Fig_1_PRISMA-Flow-Chart.jpeg]

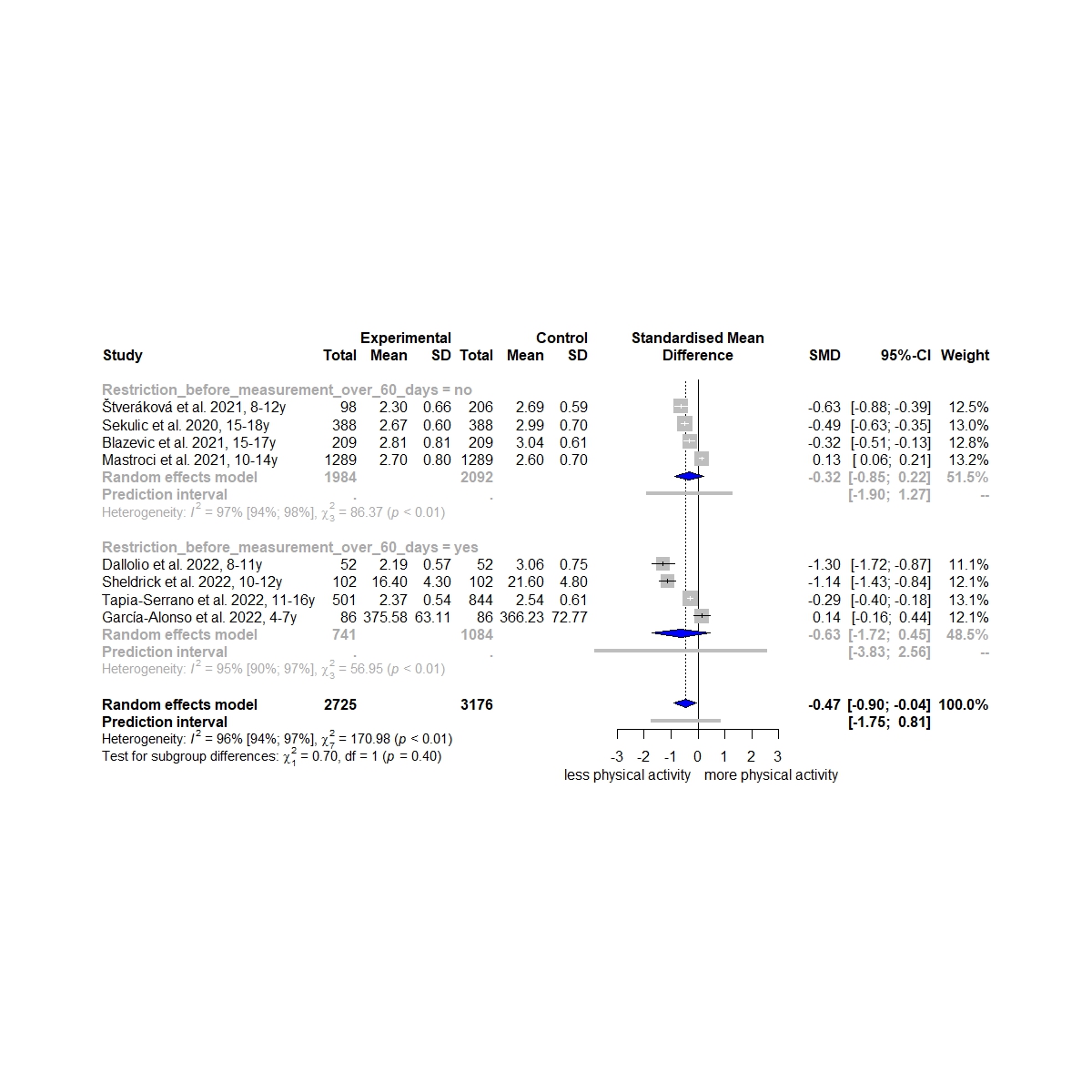

Supplement: Supplementary file 1 — Additional file 1: Table S1. PRISMA item checklist for systematic reviews. Table S2. Deviations from the systematic review protocol. Table S3. Searched websites of key organizations. Table S4. Search strategy. Table S5. Reasons for exclusion of studies from the systematic literature search, after full-text screening. Table S6. Data conversion. Table S7. Criteria for grading evidence according to Grading of Recommendations, Assessment, Development and Evaluations (GRADE). Table S8. Evidence profile for grading evidence according to Grading of Recommendations, Assessment, Development and Evaluations (GRADE). Table S9. Summary of effect estimates. Table S10. Meta-regression for total physical activity with categorical moderators. Table S11. Meta-regression for total physical activity with continuous moderators. Table S12. Meta-regression for moderate-to-vigorous physical activity with categorical moderators. Table S13. Meta-regression for moderate-to-vigorous physical activity with continuous moderators. Table S14. Sensitivity analysis for total physical activity. Table S15. Sensitivity analysis for moderate-to-vigorous physical activity. Table S16. Eggers’ test. Figure S1. PRISMA Flow Chart. Figure S2. Graphical distribution of the studies included. Figure S3. Traffic-light plots of the domain-level judgements for each individual result. Figure S4. Weighted-bar plots of the distribution of risk of bias judgements within each bias domain. Figure S5. Forest plot of changes in total physical activity comparing before and during COVID-19 pandemic, using Physical Activity Questionnaire for Children and Adolescents. Figure S6. Forest plot of changes in total physical activity comparing before and during COVID-19 pandemic, using accelerometer measurements. Figure S7. Forest plot of changes in female and male total physical activity comparing before and during COVID-19 pandemic. Figure S8. Forest plot of changes according to time course in total physical activity comparing be [file 12966_2023_1542_MOESM1_ESM.zip › S-Fig_10_ForestPlot_TPA_Restriction_60.jpeg]

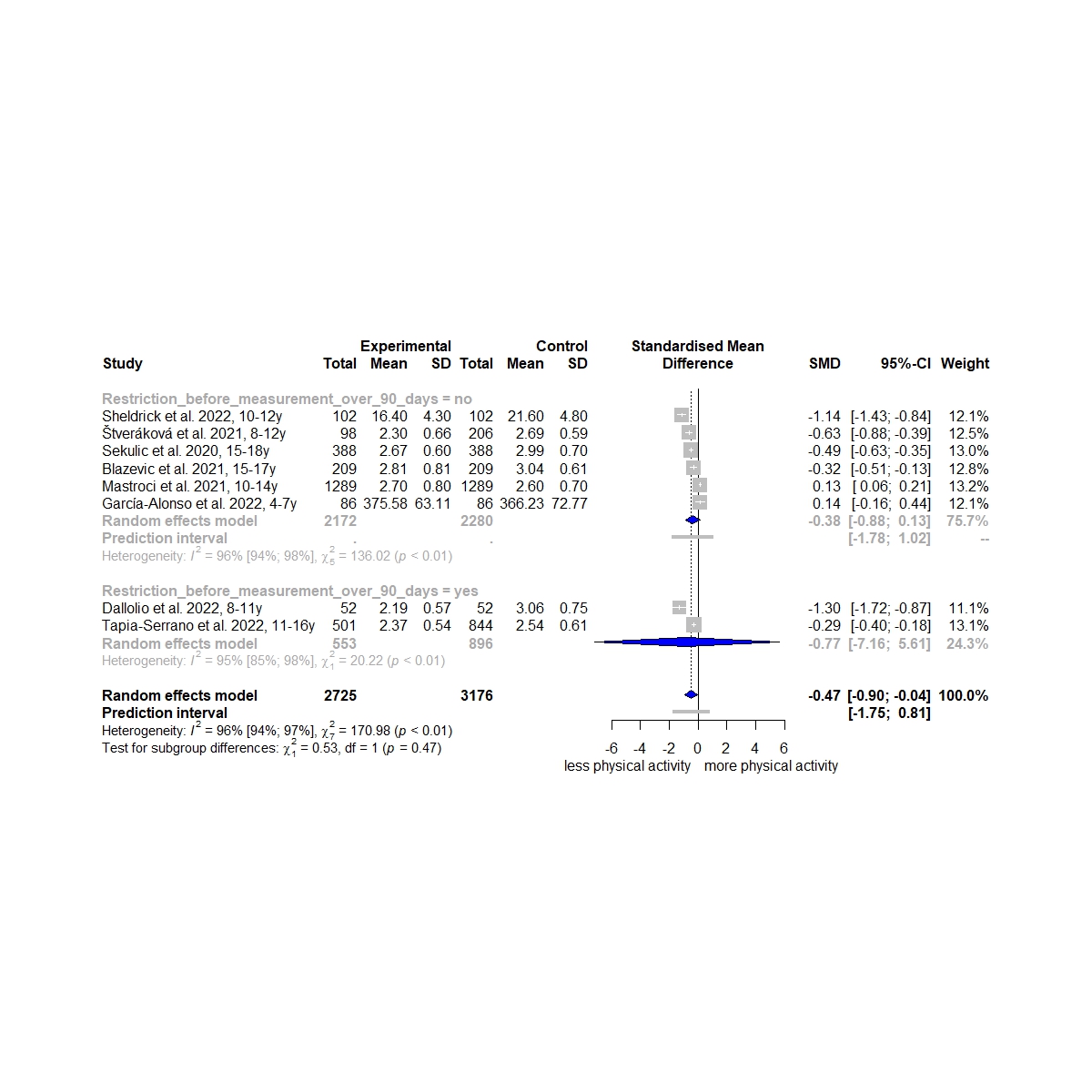

Supplement: Supplementary file 1 — Additional file 1: Table S1. PRISMA item checklist for systematic reviews. Table S2. Deviations from the systematic review protocol. Table S3. Searched websites of key organizations. Table S4. Search strategy. Table S5. Reasons for exclusion of studies from the systematic literature search, after full-text screening. Table S6. Data conversion. Table S7. Criteria for grading evidence according to Grading of Recommendations, Assessment, Development and Evaluations (GRADE). Table S8. Evidence profile for grading evidence according to Grading of Recommendations, Assessment, Development and Evaluations (GRADE). Table S9. Summary of effect estimates. Table S10. Meta-regression for total physical activity with categorical moderators. Table S11. Meta-regression for total physical activity with continuous moderators. Table S12. Meta-regression for moderate-to-vigorous physical activity with categorical moderators. Table S13. Meta-regression for moderate-to-vigorous physical activity with continuous moderators. Table S14. Sensitivity analysis for total physical activity. Table S15. Sensitivity analysis for moderate-to-vigorous physical activity. Table S16. Eggers’ test. Figure S1. PRISMA Flow Chart. Figure S2. Graphical distribution of the studies included. Figure S3. Traffic-light plots of the domain-level judgements for each individual result. Figure S4. Weighted-bar plots of the distribution of risk of bias judgements within each bias domain. Figure S5. Forest plot of changes in total physical activity comparing before and during COVID-19 pandemic, using Physical Activity Questionnaire for Children and Adolescents. Figure S6. Forest plot of changes in total physical activity comparing before and during COVID-19 pandemic, using accelerometer measurements. Figure S7. Forest plot of changes in female and male total physical activity comparing before and during COVID-19 pandemic. Figure S8. Forest plot of changes according to time course in total physical activity comparing be [file 12966_2023_1542_MOESM1_ESM.zip › S-Fig_11_ForestPlot_TPA_Restriction_90.jpeg]

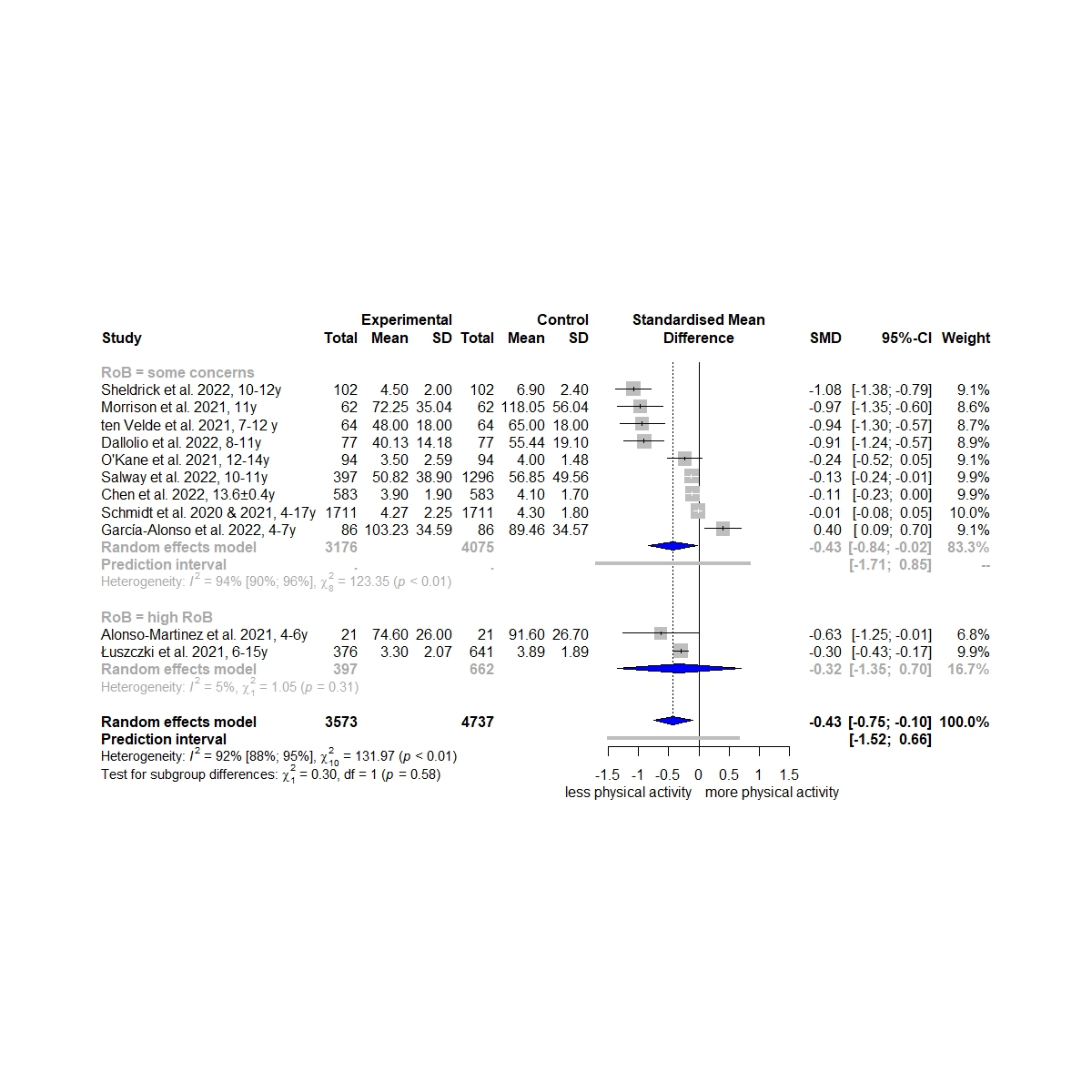

Supplement: Supplementary file 1 — Additional file 1: Table S1. PRISMA item checklist for systematic reviews. Table S2. Deviations from the systematic review protocol. Table S3. Searched websites of key organizations. Table S4. Search strategy. Table S5. Reasons for exclusion of studies from the systematic literature search, after full-text screening. Table S6. Data conversion. Table S7. Criteria for grading evidence according to Grading of Recommendations, Assessment, Development and Evaluations (GRADE). Table S8. Evidence profile for grading evidence according to Grading of Recommendations, Assessment, Development and Evaluations (GRADE). Table S9. Summary of effect estimates. Table S10. Meta-regression for total physical activity with categorical moderators. Table S11. Meta-regression for total physical activity with continuous moderators. Table S12. Meta-regression for moderate-to-vigorous physical activity with categorical moderators. Table S13. Meta-regression for moderate-to-vigorous physical activity with continuous moderators. Table S14. Sensitivity analysis for total physical activity. Table S15. Sensitivity analysis for moderate-to-vigorous physical activity. Table S16. Eggers’ test. Figure S1. PRISMA Flow Chart. Figure S2. Graphical distribution of the studies included. Figure S3. Traffic-light plots of the domain-level judgements for each individual result. Figure S4. Weighted-bar plots of the distribution of risk of bias judgements within each bias domain. Figure S5. Forest plot of changes in total physical activity comparing before and during COVID-19 pandemic, using Physical Activity Questionnaire for Children and Adolescents. Figure S6. Forest plot of changes in total physical activity comparing before and during COVID-19 pandemic, using accelerometer measurements. Figure S7. Forest plot of changes in female and male total physical activity comparing before and during COVID-19 pandemic. Figure S8. Forest plot of changes according to time course in total physical activity comparing be [file 12966_2023_1542_MOESM1_ESM.zip › S-Fig_12_PhysAct_MVPA_total_RoB.jpeg]

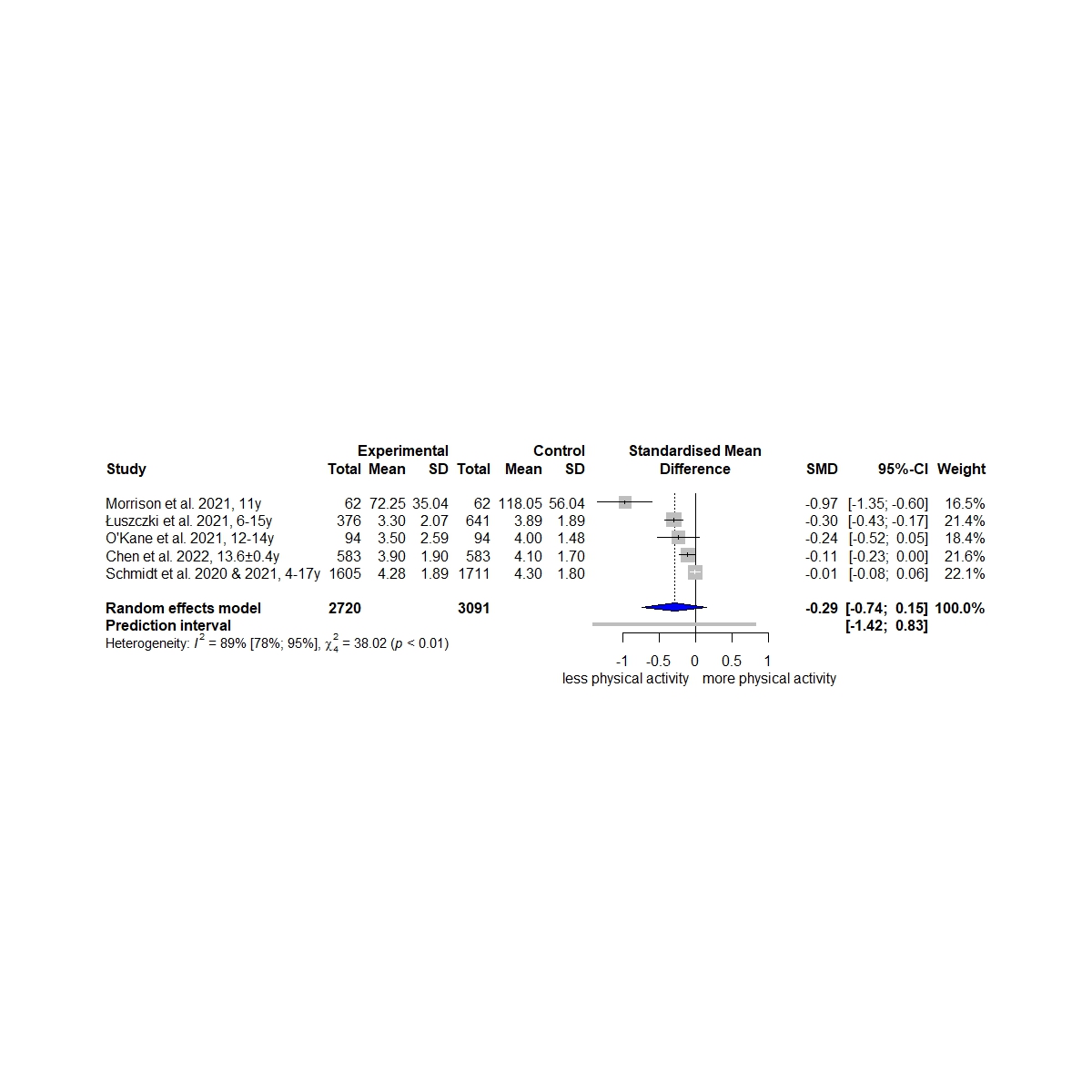

Supplement: Supplementary file 1 — Additional file 1: Table S1. PRISMA item checklist for systematic reviews. Table S2. Deviations from the systematic review protocol. Table S3. Searched websites of key organizations. Table S4. Search strategy. Table S5. Reasons for exclusion of studies from the systematic literature search, after full-text screening. Table S6. Data conversion. Table S7. Criteria for grading evidence according to Grading of Recommendations, Assessment, Development and Evaluations (GRADE). Table S8. Evidence profile for grading evidence according to Grading of Recommendations, Assessment, Development and Evaluations (GRADE). Table S9. Summary of effect estimates. Table S10. Meta-regression for total physical activity with categorical moderators. Table S11. Meta-regression for total physical activity with continuous moderators. Table S12. Meta-regression for moderate-to-vigorous physical activity with categorical moderators. Table S13. Meta-regression for moderate-to-vigorous physical activity with continuous moderators. Table S14. Sensitivity analysis for total physical activity. Table S15. Sensitivity analysis for moderate-to-vigorous physical activity. Table S16. Eggers’ test. Figure S1. PRISMA Flow Chart. Figure S2. Graphical distribution of the studies included. Figure S3. Traffic-light plots of the domain-level judgements for each individual result. Figure S4. Weighted-bar plots of the distribution of risk of bias judgements within each bias domain. Figure S5. Forest plot of changes in total physical activity comparing before and during COVID-19 pandemic, using Physical Activity Questionnaire for Children and Adolescents. Figure S6. Forest plot of changes in total physical activity comparing before and during COVID-19 pandemic, using accelerometer measurements. Figure S7. Forest plot of changes in female and male total physical activity comparing before and during COVID-19 pandemic. Figure S8. Forest plot of changes according to time course in total physical activity comparing be [file 12966_2023_1542_MOESM1_ESM.zip › S-Fig_13_PhysAct_MVPA_total_score.jpeg]

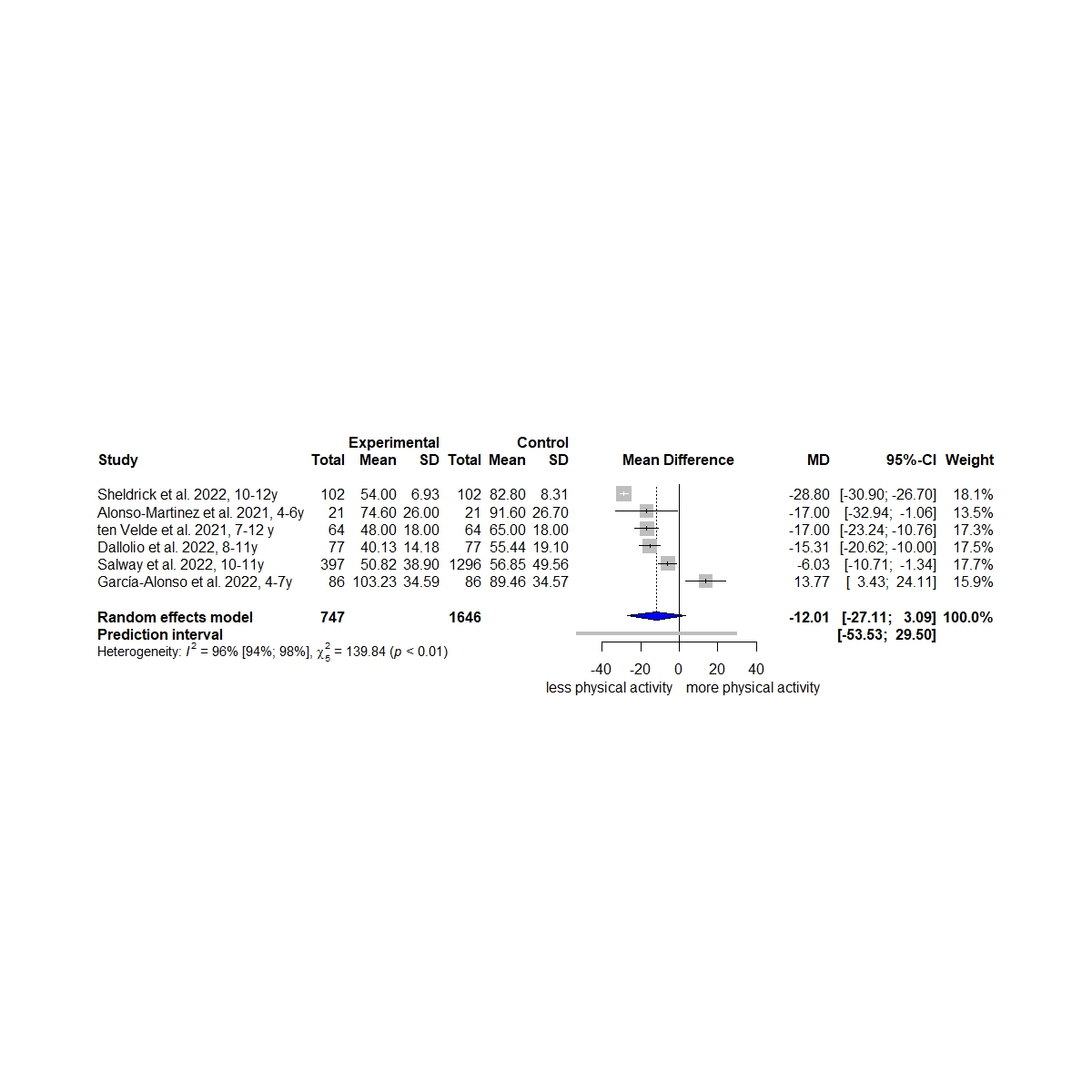

Supplement: Supplementary file 1 — Additional file 1: Table S1. PRISMA item checklist for systematic reviews. Table S2. Deviations from the systematic review protocol. Table S3. Searched websites of key organizations. Table S4. Search strategy. Table S5. Reasons for exclusion of studies from the systematic literature search, after full-text screening. Table S6. Data conversion. Table S7. Criteria for grading evidence according to Grading of Recommendations, Assessment, Development and Evaluations (GRADE). Table S8. Evidence profile for grading evidence according to Grading of Recommendations, Assessment, Development and Evaluations (GRADE). Table S9. Summary of effect estimates. Table S10. Meta-regression for total physical activity with categorical moderators. Table S11. Meta-regression for total physical activity with continuous moderators. Table S12. Meta-regression for moderate-to-vigorous physical activity with categorical moderators. Table S13. Meta-regression for moderate-to-vigorous physical activity with continuous moderators. Table S14. Sensitivity analysis for total physical activity. Table S15. Sensitivity analysis for moderate-to-vigorous physical activity. Table S16. Eggers’ test. Figure S1. PRISMA Flow Chart. Figure S2. Graphical distribution of the studies included. Figure S3. Traffic-light plots of the domain-level judgements for each individual result. Figure S4. Weighted-bar plots of the distribution of risk of bias judgements within each bias domain. Figure S5. Forest plot of changes in total physical activity comparing before and during COVID-19 pandemic, using Physical Activity Questionnaire for Children and Adolescents. Figure S6. Forest plot of changes in total physical activity comparing before and during COVID-19 pandemic, using accelerometer measurements. Figure S7. Forest plot of changes in female and male total physical activity comparing before and during COVID-19 pandemic. Figure S8. Forest plot of changes according to time course in total physical activity comparing be [file 12966_2023_1542_MOESM1_ESM.zip › S-Fig_14_PhysAct_MVPA_total_accel.jpeg]

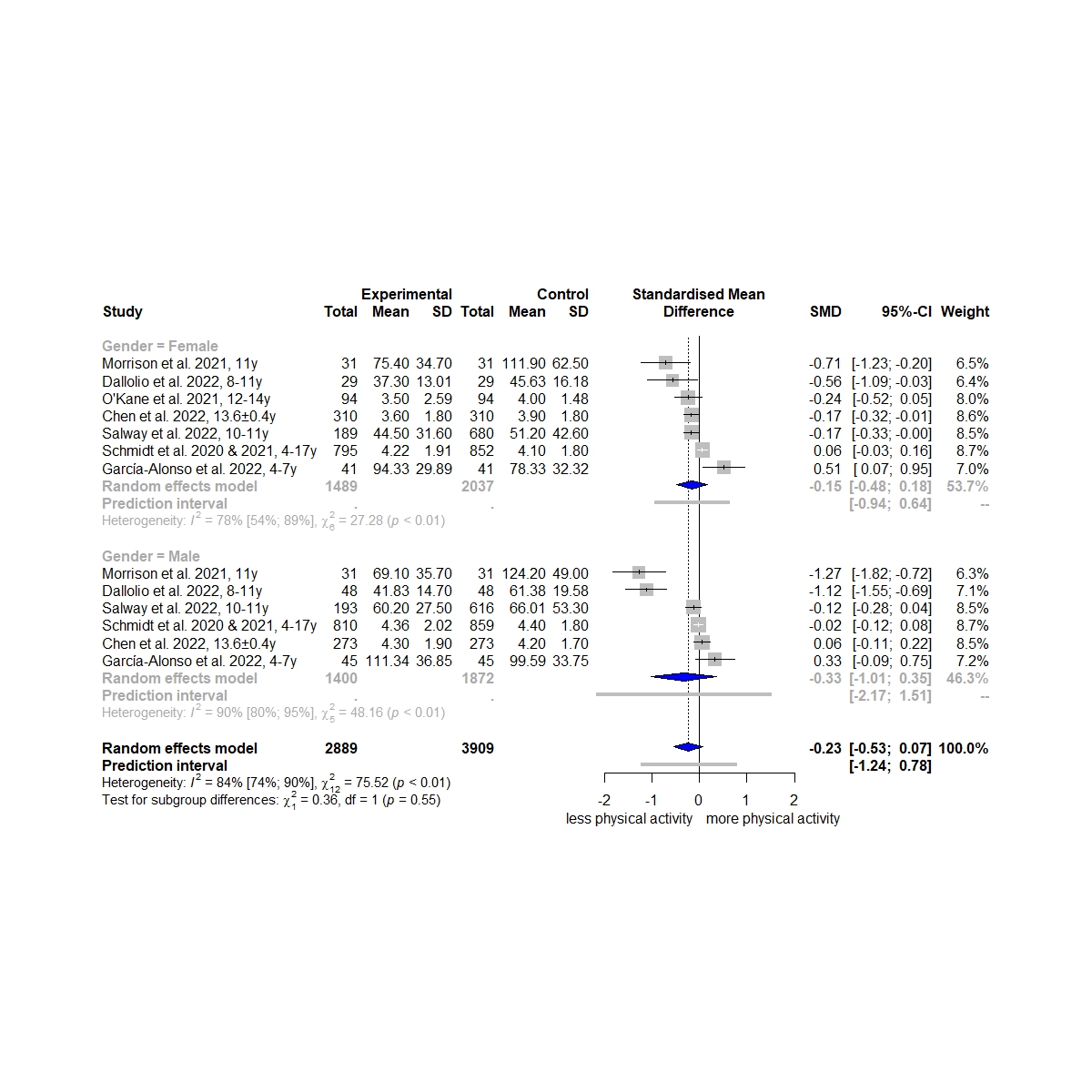

Supplement: Supplementary file 1 — Additional file 1: Table S1. PRISMA item checklist for systematic reviews. Table S2. Deviations from the systematic review protocol. Table S3. Searched websites of key organizations. Table S4. Search strategy. Table S5. Reasons for exclusion of studies from the systematic literature search, after full-text screening. Table S6. Data conversion. Table S7. Criteria for grading evidence according to Grading of Recommendations, Assessment, Development and Evaluations (GRADE). Table S8. Evidence profile for grading evidence according to Grading of Recommendations, Assessment, Development and Evaluations (GRADE). Table S9. Summary of effect estimates. Table S10. Meta-regression for total physical activity with categorical moderators. Table S11. Meta-regression for total physical activity with continuous moderators. Table S12. Meta-regression for moderate-to-vigorous physical activity with categorical moderators. Table S13. Meta-regression for moderate-to-vigorous physical activity with continuous moderators. Table S14. Sensitivity analysis for total physical activity. Table S15. Sensitivity analysis for moderate-to-vigorous physical activity. Table S16. Eggers’ test. Figure S1. PRISMA Flow Chart. Figure S2. Graphical distribution of the studies included. Figure S3. Traffic-light plots of the domain-level judgements for each individual result. Figure S4. Weighted-bar plots of the distribution of risk of bias judgements within each bias domain. Figure S5. Forest plot of changes in total physical activity comparing before and during COVID-19 pandemic, using Physical Activity Questionnaire for Children and Adolescents. Figure S6. Forest plot of changes in total physical activity comparing before and during COVID-19 pandemic, using accelerometer measurements. Figure S7. Forest plot of changes in female and male total physical activity comparing before and during COVID-19 pandemic. Figure S8. Forest plot of changes according to time course in total physical activity comparing be [file 12966_2023_1542_MOESM1_ESM.zip › S-Fig_15_ForestPlot_MVPA_FemaleMale.jpeg]

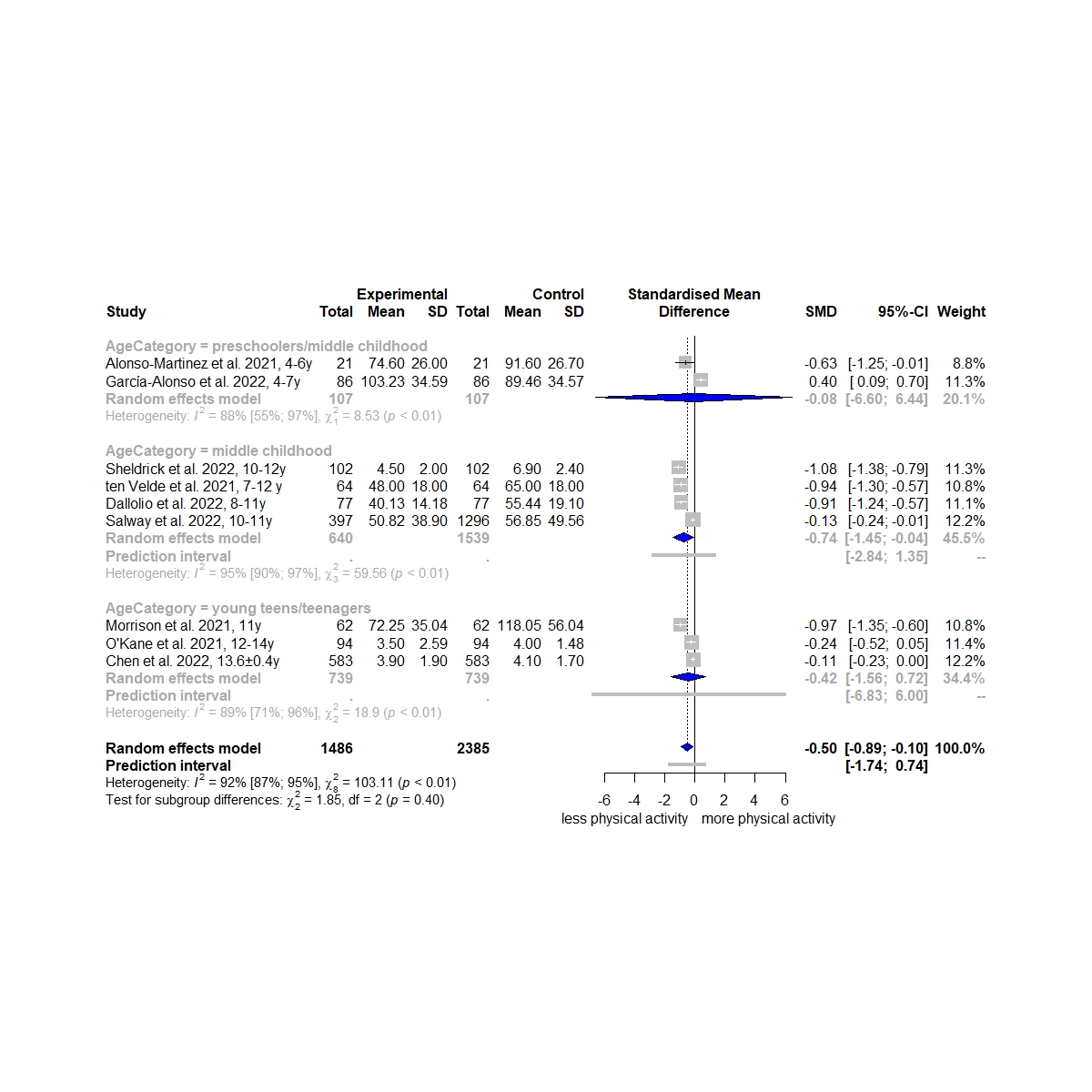

Supplement: Supplementary file 1 — Additional file 1: Table S1. PRISMA item checklist for systematic reviews. Table S2. Deviations from the systematic review protocol. Table S3. Searched websites of key organizations. Table S4. Search strategy. Table S5. Reasons for exclusion of studies from the systematic literature search, after full-text screening. Table S6. Data conversion. Table S7. Criteria for grading evidence according to Grading of Recommendations, Assessment, Development and Evaluations (GRADE). Table S8. Evidence profile for grading evidence according to Grading of Recommendations, Assessment, Development and Evaluations (GRADE). Table S9. Summary of effect estimates. Table S10. Meta-regression for total physical activity with categorical moderators. Table S11. Meta-regression for total physical activity with continuous moderators. Table S12. Meta-regression for moderate-to-vigorous physical activity with categorical moderators. Table S13. Meta-regression for moderate-to-vigorous physical activity with continuous moderators. Table S14. Sensitivity analysis for total physical activity. Table S15. Sensitivity analysis for moderate-to-vigorous physical activity. Table S16. Eggers’ test. Figure S1. PRISMA Flow Chart. Figure S2. Graphical distribution of the studies included. Figure S3. Traffic-light plots of the domain-level judgements for each individual result. Figure S4. Weighted-bar plots of the distribution of risk of bias judgements within each bias domain. Figure S5. Forest plot of changes in total physical activity comparing before and during COVID-19 pandemic, using Physical Activity Questionnaire for Children and Adolescents. Figure S6. Forest plot of changes in total physical activity comparing before and during COVID-19 pandemic, using accelerometer measurements. Figure S7. Forest plot of changes in female and male total physical activity comparing before and during COVID-19 pandemic. Figure S8. Forest plot of changes according to time course in total physical activity comparing be [file 12966_2023_1542_MOESM1_ESM.zip › S-Fig_16_ForestPlot_MVPA_Age.jpeg]

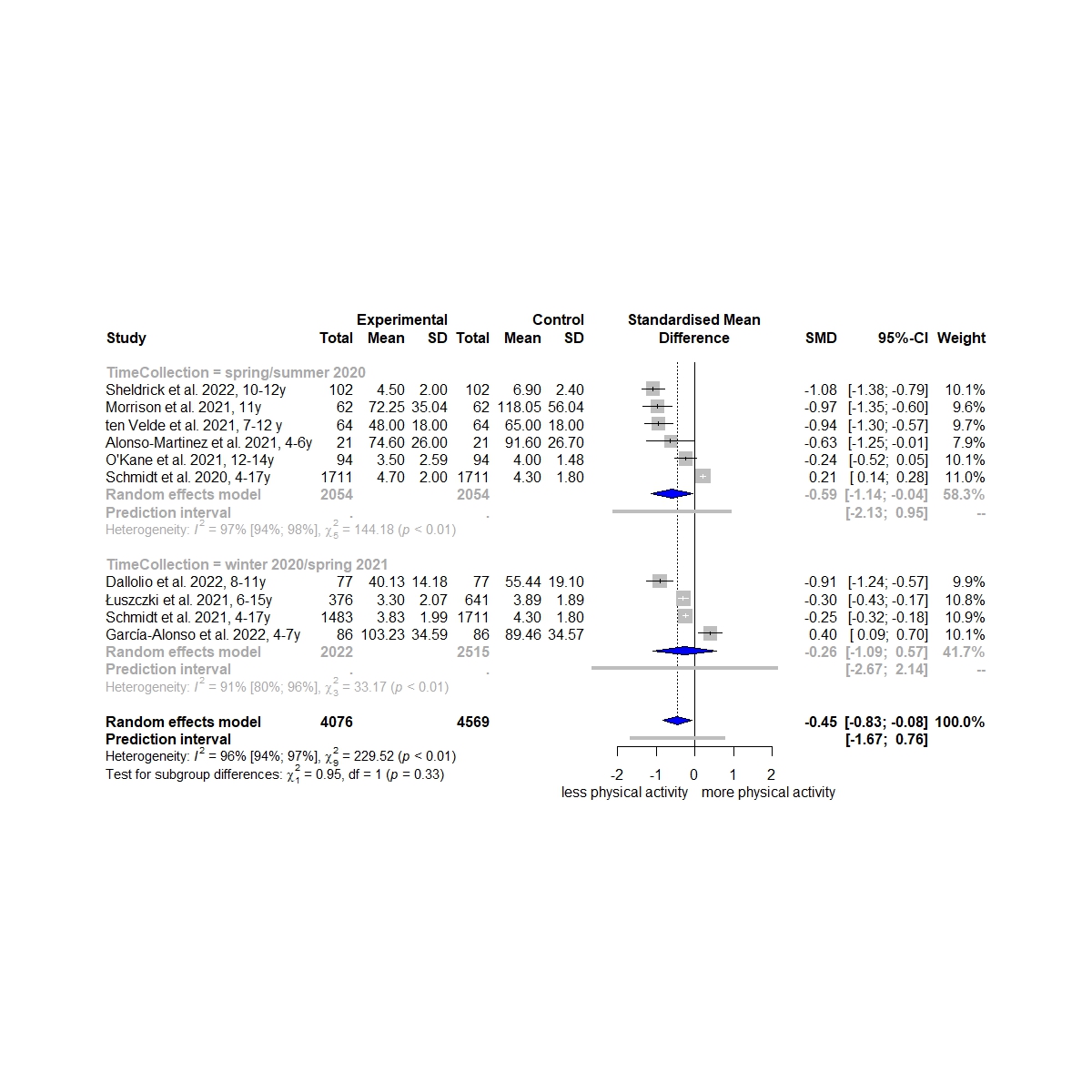

Supplement: Supplementary file 1 — Additional file 1: Table S1. PRISMA item checklist for systematic reviews. Table S2. Deviations from the systematic review protocol. Table S3. Searched websites of key organizations. Table S4. Search strategy. Table S5. Reasons for exclusion of studies from the systematic literature search, after full-text screening. Table S6. Data conversion. Table S7. Criteria for grading evidence according to Grading of Recommendations, Assessment, Development and Evaluations (GRADE). Table S8. Evidence profile for grading evidence according to Grading of Recommendations, Assessment, Development and Evaluations (GRADE). Table S9. Summary of effect estimates. Table S10. Meta-regression for total physical activity with categorical moderators. Table S11. Meta-regression for total physical activity with continuous moderators. Table S12. Meta-regression for moderate-to-vigorous physical activity with categorical moderators. Table S13. Meta-regression for moderate-to-vigorous physical activity with continuous moderators. Table S14. Sensitivity analysis for total physical activity. Table S15. Sensitivity analysis for moderate-to-vigorous physical activity. Table S16. Eggers’ test. Figure S1. PRISMA Flow Chart. Figure S2. Graphical distribution of the studies included. Figure S3. Traffic-light plots of the domain-level judgements for each individual result. Figure S4. Weighted-bar plots of the distribution of risk of bias judgements within each bias domain. Figure S5. Forest plot of changes in total physical activity comparing before and during COVID-19 pandemic, using Physical Activity Questionnaire for Children and Adolescents. Figure S6. Forest plot of changes in total physical activity comparing before and during COVID-19 pandemic, using accelerometer measurements. Figure S7. Forest plot of changes in female and male total physical activity comparing before and during COVID-19 pandemic. Figure S8. Forest plot of changes according to time course in total physical activity comparing be [file 12966_2023_1542_MOESM1_ESM.zip › S-Fig_17_PhysAct_MVPA_time.jpeg]

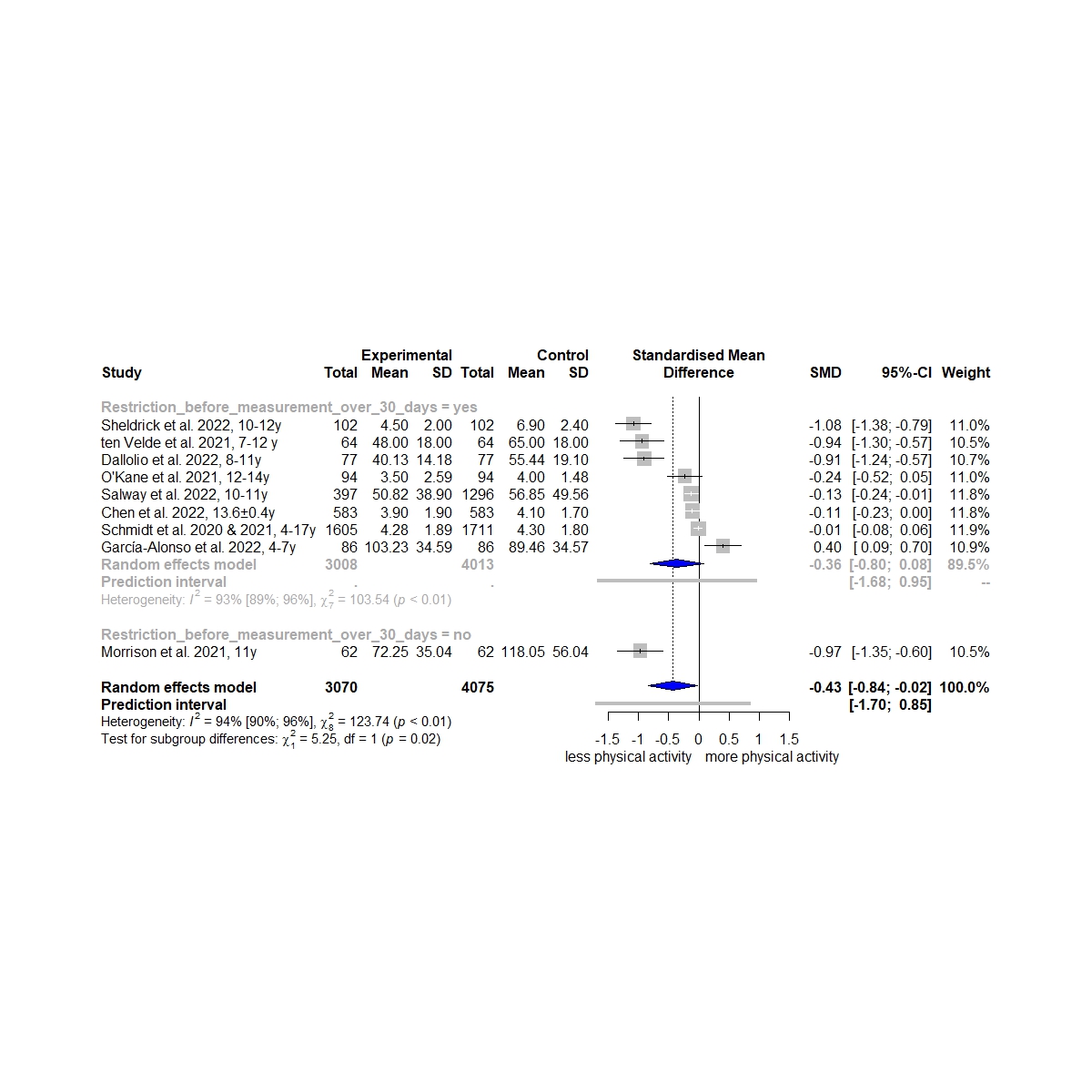

Supplement: Supplementary file 1 — Additional file 1: Table S1. PRISMA item checklist for systematic reviews. Table S2. Deviations from the systematic review protocol. Table S3. Searched websites of key organizations. Table S4. Search strategy. Table S5. Reasons for exclusion of studies from the systematic literature search, after full-text screening. Table S6. Data conversion. Table S7. Criteria for grading evidence according to Grading of Recommendations, Assessment, Development and Evaluations (GRADE). Table S8. Evidence profile for grading evidence according to Grading of Recommendations, Assessment, Development and Evaluations (GRADE). Table S9. Summary of effect estimates. Table S10. Meta-regression for total physical activity with categorical moderators. Table S11. Meta-regression for total physical activity with continuous moderators. Table S12. Meta-regression for moderate-to-vigorous physical activity with categorical moderators. Table S13. Meta-regression for moderate-to-vigorous physical activity with continuous moderators. Table S14. Sensitivity analysis for total physical activity. Table S15. Sensitivity analysis for moderate-to-vigorous physical activity. Table S16. Eggers’ test. Figure S1. PRISMA Flow Chart. Figure S2. Graphical distribution of the studies included. Figure S3. Traffic-light plots of the domain-level judgements for each individual result. Figure S4. Weighted-bar plots of the distribution of risk of bias judgements within each bias domain. Figure S5. Forest plot of changes in total physical activity comparing before and during COVID-19 pandemic, using Physical Activity Questionnaire for Children and Adolescents. Figure S6. Forest plot of changes in total physical activity comparing before and during COVID-19 pandemic, using accelerometer measurements. Figure S7. Forest plot of changes in female and male total physical activity comparing before and during COVID-19 pandemic. Figure S8. Forest plot of changes according to time course in total physical activity comparing be [file 12966_2023_1542_MOESM1_ESM.zip › S-Fig_18_ForestPlot_MVPA_Restriction_30.jpeg]

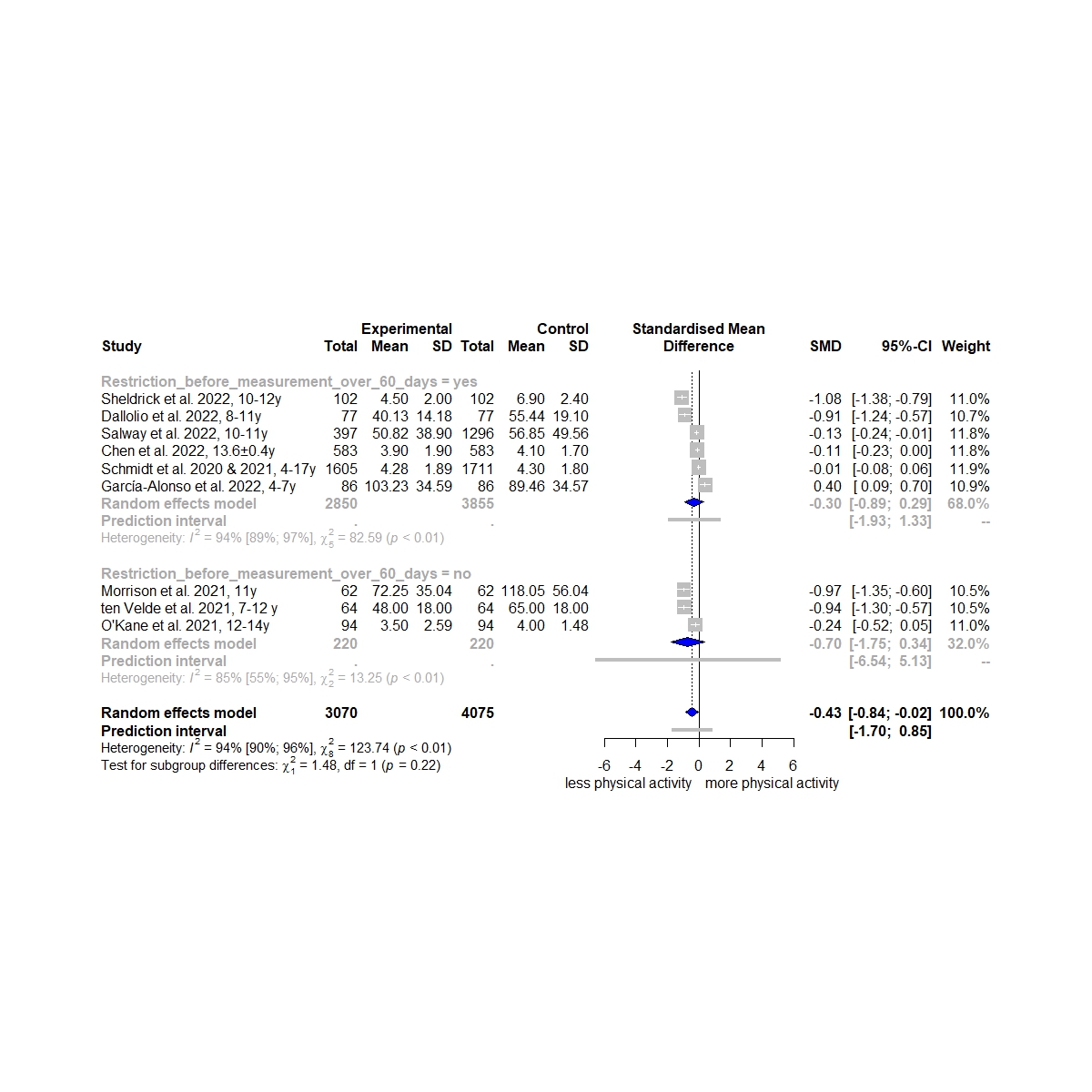

Supplement: Supplementary file 1 — Additional file 1: Table S1. PRISMA item checklist for systematic reviews. Table S2. Deviations from the systematic review protocol. Table S3. Searched websites of key organizations. Table S4. Search strategy. Table S5. Reasons for exclusion of studies from the systematic literature search, after full-text screening. Table S6. Data conversion. Table S7. Criteria for grading evidence according to Grading of Recommendations, Assessment, Development and Evaluations (GRADE). Table S8. Evidence profile for grading evidence according to Grading of Recommendations, Assessment, Development and Evaluations (GRADE). Table S9. Summary of effect estimates. Table S10. Meta-regression for total physical activity with categorical moderators. Table S11. Meta-regression for total physical activity with continuous moderators. Table S12. Meta-regression for moderate-to-vigorous physical activity with categorical moderators. Table S13. Meta-regression for moderate-to-vigorous physical activity with continuous moderators. Table S14. Sensitivity analysis for total physical activity. Table S15. Sensitivity analysis for moderate-to-vigorous physical activity. Table S16. Eggers’ test. Figure S1. PRISMA Flow Chart. Figure S2. Graphical distribution of the studies included. Figure S3. Traffic-light plots of the domain-level judgements for each individual result. Figure S4. Weighted-bar plots of the distribution of risk of bias judgements within each bias domain. Figure S5. Forest plot of changes in total physical activity comparing before and during COVID-19 pandemic, using Physical Activity Questionnaire for Children and Adolescents. Figure S6. Forest plot of changes in total physical activity comparing before and during COVID-19 pandemic, using accelerometer measurements. Figure S7. Forest plot of changes in female and male total physical activity comparing before and during COVID-19 pandemic. Figure S8. Forest plot of changes according to time course in total physical activity comparing be [file 12966_2023_1542_MOESM1_ESM.zip › S-Fig_19_ForestPlot_MVPA_Restriction_60.jpeg]

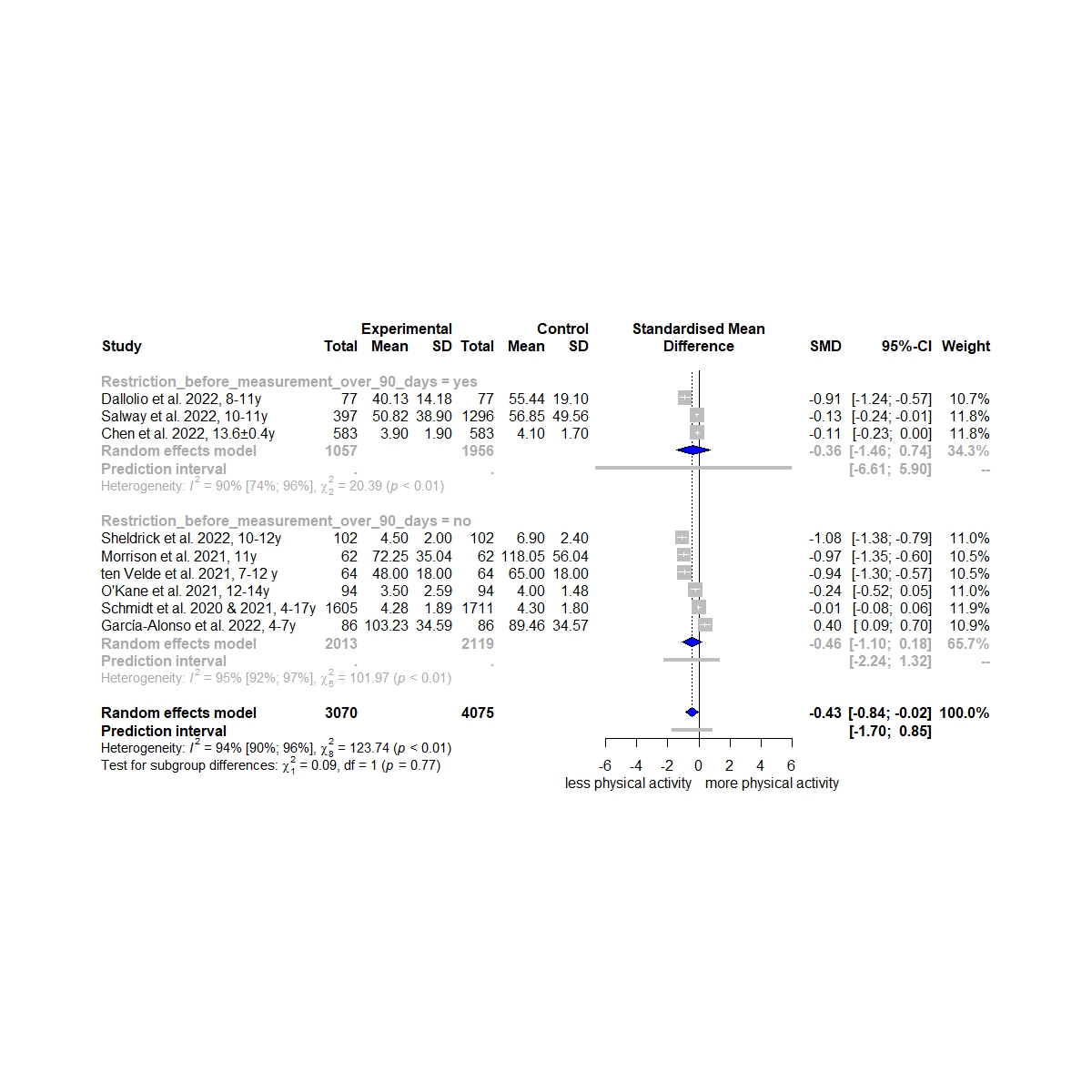

Supplement: Supplementary file 1 — Additional file 1: Table S1. PRISMA item checklist for systematic reviews. Table S2. Deviations from the systematic review protocol. Table S3. Searched websites of key organizations. Table S4. Search strategy. Table S5. Reasons for exclusion of studies from the systematic literature search, after full-text screening. Table S6. Data conversion. Table S7. Criteria for grading evidence according to Grading of Recommendations, Assessment, Development and Evaluations (GRADE). Table S8. Evidence profile for grading evidence according to Grading of Recommendations, Assessment, Development and Evaluations (GRADE). Table S9. Summary of effect estimates. Table S10. Meta-regression for total physical activity with categorical moderators. Table S11. Meta-regression for total physical activity with continuous moderators. Table S12. Meta-regression for moderate-to-vigorous physical activity with categorical moderators. Table S13. Meta-regression for moderate-to-vigorous physical activity with continuous moderators. Table S14. Sensitivity analysis for total physical activity. Table S15. Sensitivity analysis for moderate-to-vigorous physical activity. Table S16. Eggers’ test. Figure S1. PRISMA Flow Chart. Figure S2. Graphical distribution of the studies included. Figure S3. Traffic-light plots of the domain-level judgements for each individual result. Figure S4. Weighted-bar plots of the distribution of risk of bias judgements within each bias domain. Figure S5. Forest plot of changes in total physical activity comparing before and during COVID-19 pandemic, using Physical Activity Questionnaire for Children and Adolescents. Figure S6. Forest plot of changes in total physical activity comparing before and during COVID-19 pandemic, using accelerometer measurements. Figure S7. Forest plot of changes in female and male total physical activity comparing before and during COVID-19 pandemic. Figure S8. Forest plot of changes according to time course in total physical activity comparing be [file 12966_2023_1542_MOESM1_ESM.zip › S-Fig_20_ForestPlot_MVPA_Restriction_90.jpeg]

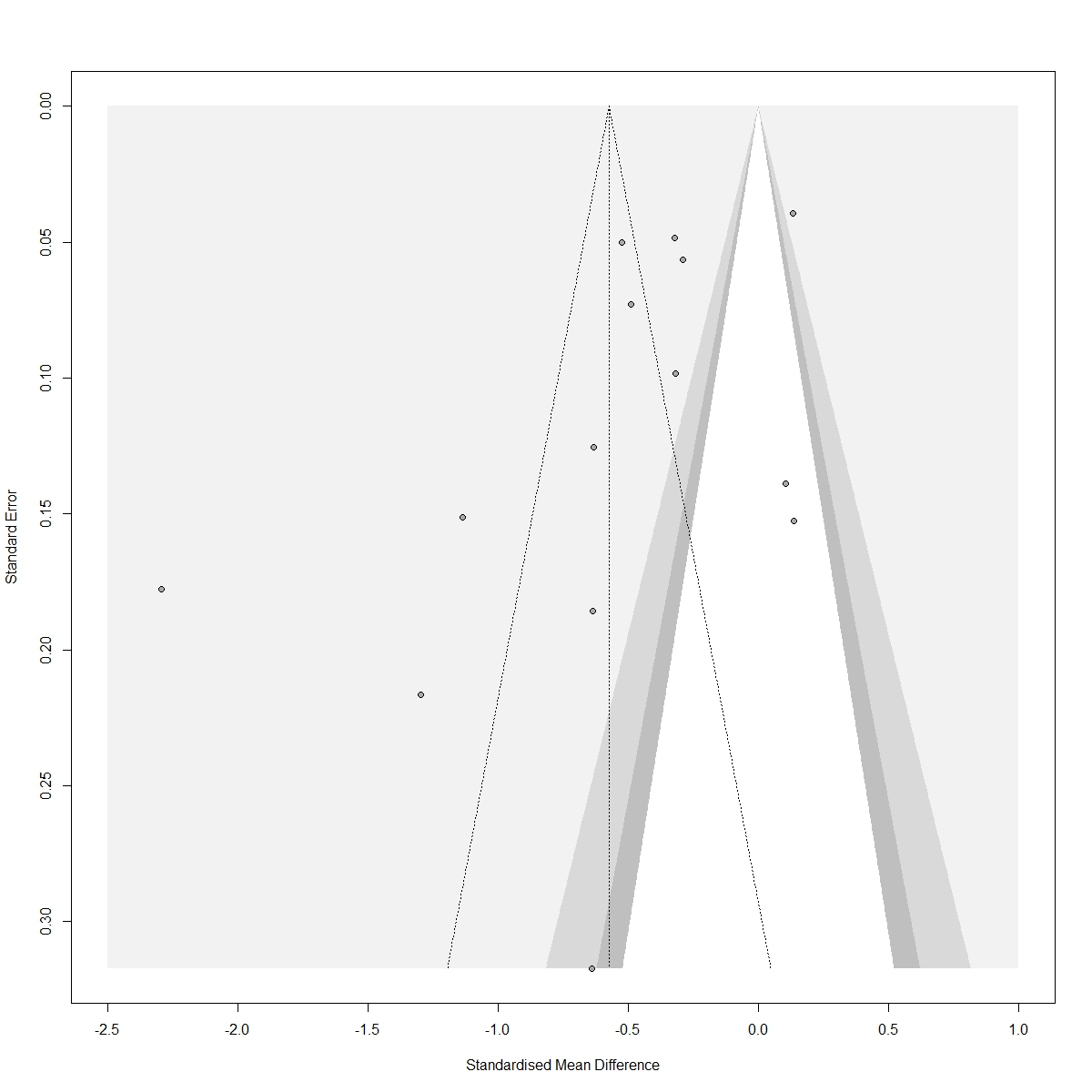

Supplement: Supplementary file 1 — Additional file 1: Table S1. PRISMA item checklist for systematic reviews. Table S2. Deviations from the systematic review protocol. Table S3. Searched websites of key organizations. Table S4. Search strategy. Table S5. Reasons for exclusion of studies from the systematic literature search, after full-text screening. Table S6. Data conversion. Table S7. Criteria for grading evidence according to Grading of Recommendations, Assessment, Development and Evaluations (GRADE). Table S8. Evidence profile for grading evidence according to Grading of Recommendations, Assessment, Development and Evaluations (GRADE). Table S9. Summary of effect estimates. Table S10. Meta-regression for total physical activity with categorical moderators. Table S11. Meta-regression for total physical activity with continuous moderators. Table S12. Meta-regression for moderate-to-vigorous physical activity with categorical moderators. Table S13. Meta-regression for moderate-to-vigorous physical activity with continuous moderators. Table S14. Sensitivity analysis for total physical activity. Table S15. Sensitivity analysis for moderate-to-vigorous physical activity. Table S16. Eggers’ test. Figure S1. PRISMA Flow Chart. Figure S2. Graphical distribution of the studies included. Figure S3. Traffic-light plots of the domain-level judgements for each individual result. Figure S4. Weighted-bar plots of the distribution of risk of bias judgements within each bias domain. Figure S5. Forest plot of changes in total physical activity comparing before and during COVID-19 pandemic, using Physical Activity Questionnaire for Children and Adolescents. Figure S6. Forest plot of changes in total physical activity comparing before and during COVID-19 pandemic, using accelerometer measurements. Figure S7. Forest plot of changes in female and male total physical activity comparing before and during COVID-19 pandemic. Figure S8. Forest plot of changes according to time course in total physical activity comparing be [file 12966_2023_1542_MOESM1_ESM.zip › S-Fig_21_FunnelPlot_TPA.jpeg]

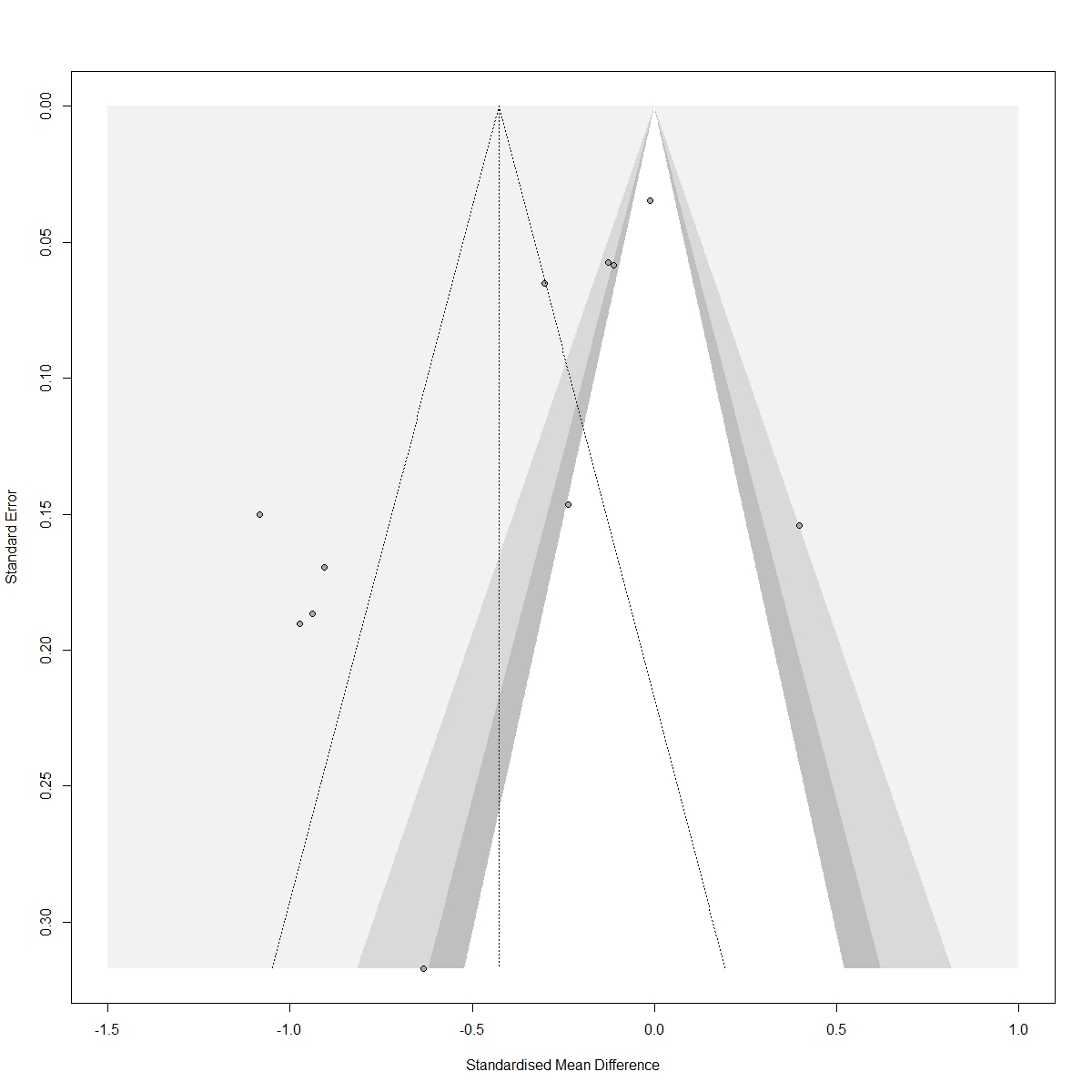

Supplement: Supplementary file 1 — Additional file 1: Table S1. PRISMA item checklist for systematic reviews. Table S2. Deviations from the systematic review protocol. Table S3. Searched websites of key organizations. Table S4. Search strategy. Table S5. Reasons for exclusion of studies from the systematic literature search, after full-text screening. Table S6. Data conversion. Table S7. Criteria for grading evidence according to Grading of Recommendations, Assessment, Development and Evaluations (GRADE). Table S8. Evidence profile for grading evidence according to Grading of Recommendations, Assessment, Development and Evaluations (GRADE). Table S9. Summary of effect estimates. Table S10. Meta-regression for total physical activity with categorical moderators. Table S11. Meta-regression for total physical activity with continuous moderators. Table S12. Meta-regression for moderate-to-vigorous physical activity with categorical moderators. Table S13. Meta-regression for moderate-to-vigorous physical activity with continuous moderators. Table S14. Sensitivity analysis for total physical activity. Table S15. Sensitivity analysis for moderate-to-vigorous physical activity. Table S16. Eggers’ test. Figure S1. PRISMA Flow Chart. Figure S2. Graphical distribution of the studies included. Figure S3. Traffic-light plots of the domain-level judgements for each individual result. Figure S4. Weighted-bar plots of the distribution of risk of bias judgements within each bias domain. Figure S5. Forest plot of changes in total physical activity comparing before and during COVID-19 pandemic, using Physical Activity Questionnaire for Children and Adolescents. Figure S6. Forest plot of changes in total physical activity comparing before and during COVID-19 pandemic, using accelerometer measurements. Figure S7. Forest plot of changes in female and male total physical activity comparing before and during COVID-19 pandemic. Figure S8. Forest plot of changes according to time course in total physical activity comparing be [file 12966_2023_1542_MOESM1_ESM.zip › S-Fig_22_FunnelPlot_MVPA.jpeg]

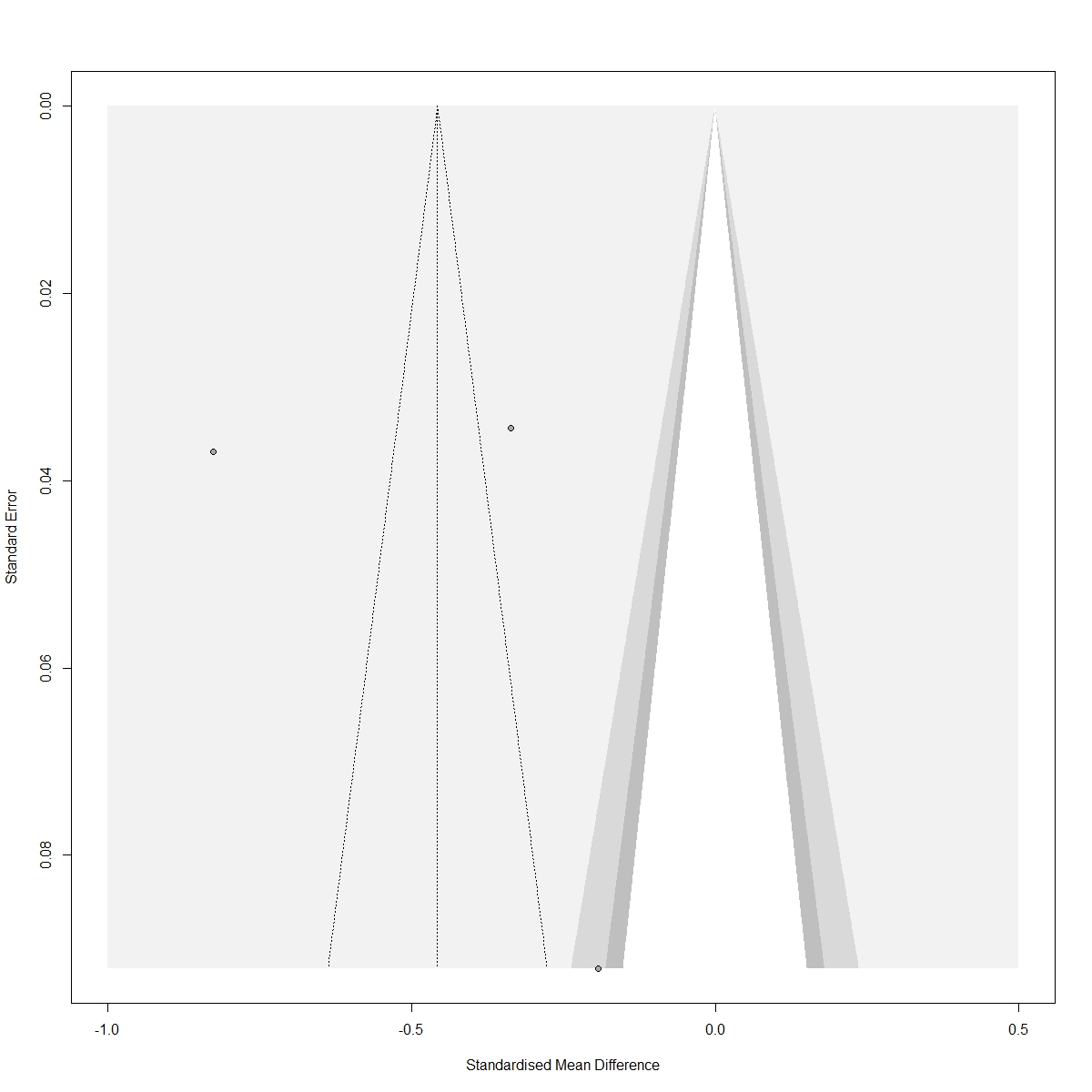

Supplement: Supplementary file 1 — Additional file 1: Table S1. PRISMA item checklist for systematic reviews. Table S2. Deviations from the systematic review protocol. Table S3. Searched websites of key organizations. Table S4. Search strategy. Table S5. Reasons for exclusion of studies from the systematic literature search, after full-text screening. Table S6. Data conversion. Table S7. Criteria for grading evidence according to Grading of Recommendations, Assessment, Development and Evaluations (GRADE). Table S8. Evidence profile for grading evidence according to Grading of Recommendations, Assessment, Development and Evaluations (GRADE). Table S9. Summary of effect estimates. Table S10. Meta-regression for total physical activity with categorical moderators. Table S11. Meta-regression for total physical activity with continuous moderators. Table S12. Meta-regression for moderate-to-vigorous physical activity with categorical moderators. Table S13. Meta-regression for moderate-to-vigorous physical activity with continuous moderators. Table S14. Sensitivity analysis for total physical activity. Table S15. Sensitivity analysis for moderate-to-vigorous physical activity. Table S16. Eggers’ test. Figure S1. PRISMA Flow Chart. Figure S2. Graphical distribution of the studies included. Figure S3. Traffic-light plots of the domain-level judgements for each individual result. Figure S4. Weighted-bar plots of the distribution of risk of bias judgements within each bias domain. Figure S5. Forest plot of changes in total physical activity comparing before and during COVID-19 pandemic, using Physical Activity Questionnaire for Children and Adolescents. Figure S6. Forest plot of changes in total physical activity comparing before and during COVID-19 pandemic, using accelerometer measurements. Figure S7. Forest plot of changes in female and male total physical activity comparing before and during COVID-19 pandemic. Figure S8. Forest plot of changes according to time course in total physical activity comparing be [file 12966_2023_1542_MOESM1_ESM.zip › S-Fig_23_Funnel_Plot_SA.jpeg]

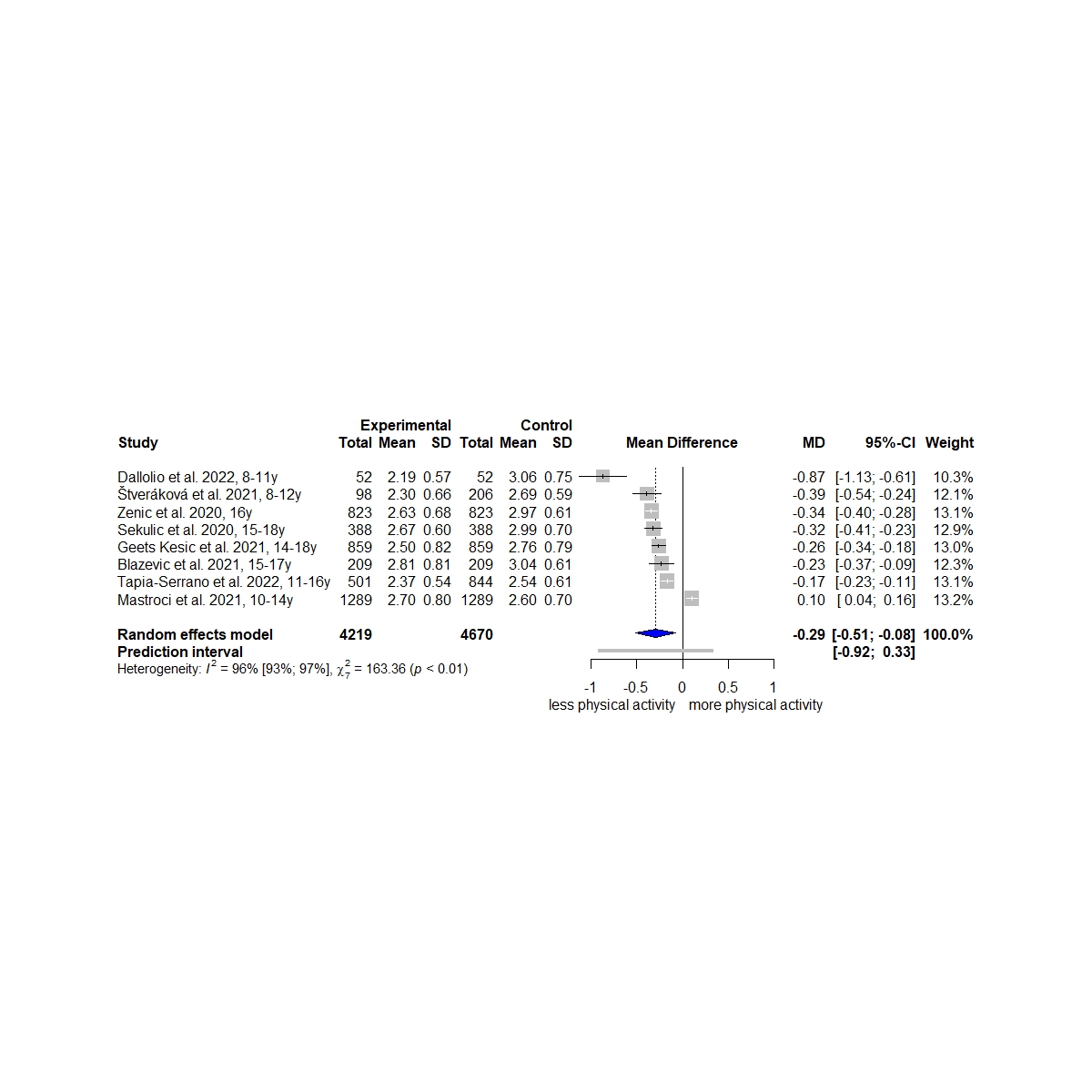

Supplement: Supplementary file 1 — Additional file 1: Table S1. PRISMA item checklist for systematic reviews. Table S2. Deviations from the systematic review protocol. Table S3. Searched websites of key organizations. Table S4. Search strategy. Table S5. Reasons for exclusion of studies from the systematic literature search, after full-text screening. Table S6. Data conversion. Table S7. Criteria for grading evidence according to Grading of Recommendations, Assessment, Development and Evaluations (GRADE). Table S8. Evidence profile for grading evidence according to Grading of Recommendations, Assessment, Development and Evaluations (GRADE). Table S9. Summary of effect estimates. Table S10. Meta-regression for total physical activity with categorical moderators. Table S11. Meta-regression for total physical activity with continuous moderators. Table S12. Meta-regression for moderate-to-vigorous physical activity with categorical moderators. Table S13. Meta-regression for moderate-to-vigorous physical activity with continuous moderators. Table S14. Sensitivity analysis for total physical activity. Table S15. Sensitivity analysis for moderate-to-vigorous physical activity. Table S16. Eggers’ test. Figure S1. PRISMA Flow Chart. Figure S2. Graphical distribution of the studies included. Figure S3. Traffic-light plots of the domain-level judgements for each individual result. Figure S4. Weighted-bar plots of the distribution of risk of bias judgements within each bias domain. Figure S5. Forest plot of changes in total physical activity comparing before and during COVID-19 pandemic, using Physical Activity Questionnaire for Children and Adolescents. Figure S6. Forest plot of changes in total physical activity comparing before and during COVID-19 pandemic, using accelerometer measurements. Figure S7. Forest plot of changes in female and male total physical activity comparing before and during COVID-19 pandemic. Figure S8. Forest plot of changes according to time course in total physical activity comparing be [file 12966_2023_1542_MOESM1_ESM.zip › S-Fig_5_ForestPlot_TPA_Score.jpeg]

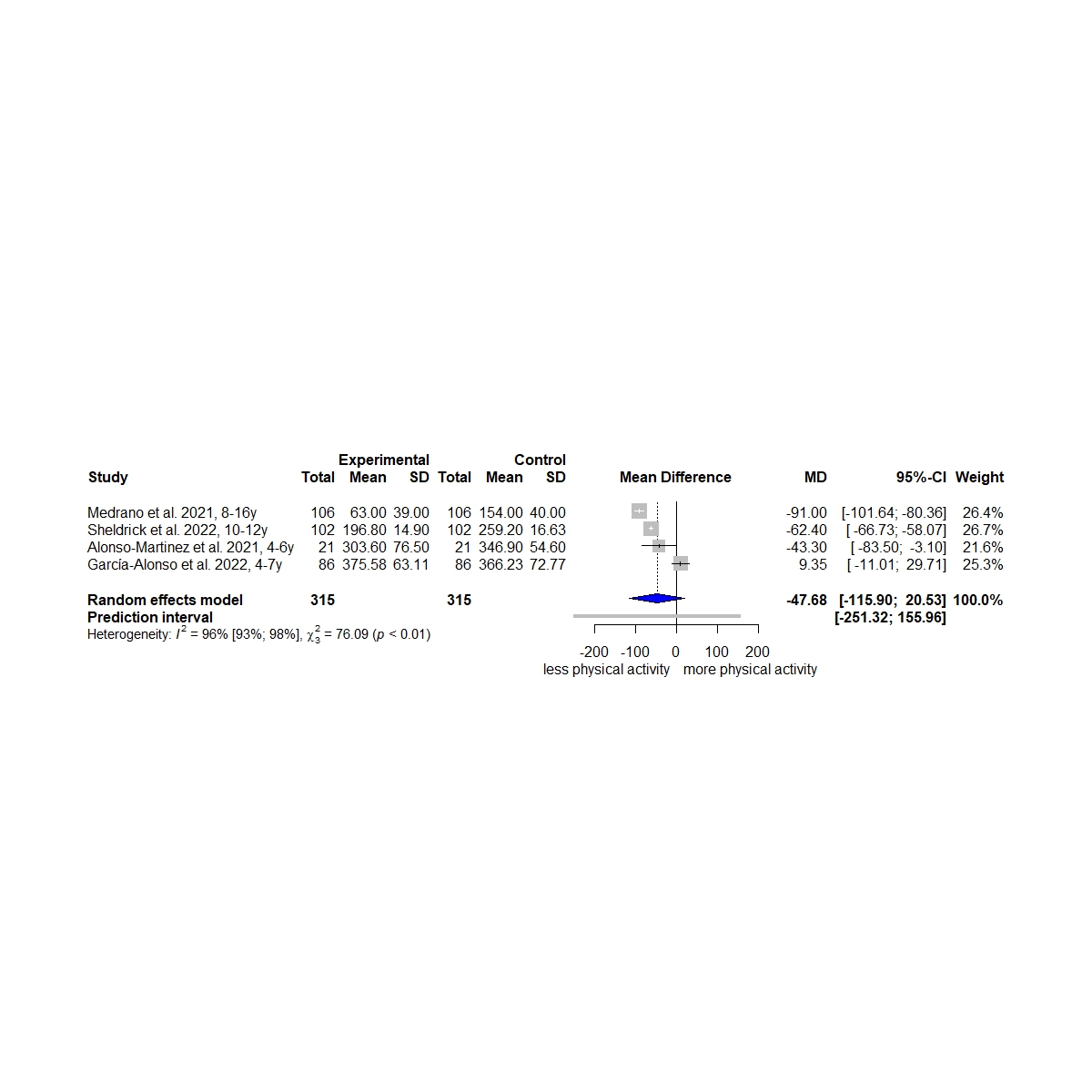

Supplement: Supplementary file 1 — Additional file 1: Table S1. PRISMA item checklist for systematic reviews. Table S2. Deviations from the systematic review protocol. Table S3. Searched websites of key organizations. Table S4. Search strategy. Table S5. Reasons for exclusion of studies from the systematic literature search, after full-text screening. Table S6. Data conversion. Table S7. Criteria for grading evidence according to Grading of Recommendations, Assessment, Development and Evaluations (GRADE). Table S8. Evidence profile for grading evidence according to Grading of Recommendations, Assessment, Development and Evaluations (GRADE). Table S9. Summary of effect estimates. Table S10. Meta-regression for total physical activity with categorical moderators. Table S11. Meta-regression for total physical activity with continuous moderators. Table S12. Meta-regression for moderate-to-vigorous physical activity with categorical moderators. Table S13. Meta-regression for moderate-to-vigorous physical activity with continuous moderators. Table S14. Sensitivity analysis for total physical activity. Table S15. Sensitivity analysis for moderate-to-vigorous physical activity. Table S16. Eggers’ test. Figure S1. PRISMA Flow Chart. Figure S2. Graphical distribution of the studies included. Figure S3. Traffic-light plots of the domain-level judgements for each individual result. Figure S4. Weighted-bar plots of the distribution of risk of bias judgements within each bias domain. Figure S5. Forest plot of changes in total physical activity comparing before and during COVID-19 pandemic, using Physical Activity Questionnaire for Children and Adolescents. Figure S6. Forest plot of changes in total physical activity comparing before and during COVID-19 pandemic, using accelerometer measurements. Figure S7. Forest plot of changes in female and male total physical activity comparing before and during COVID-19 pandemic. Figure S8. Forest plot of changes according to time course in total physical activity comparing be [file 12966_2023_1542_MOESM1_ESM.zip › S-Fig_6_ForestPlot_TPA_Accel.jpeg]

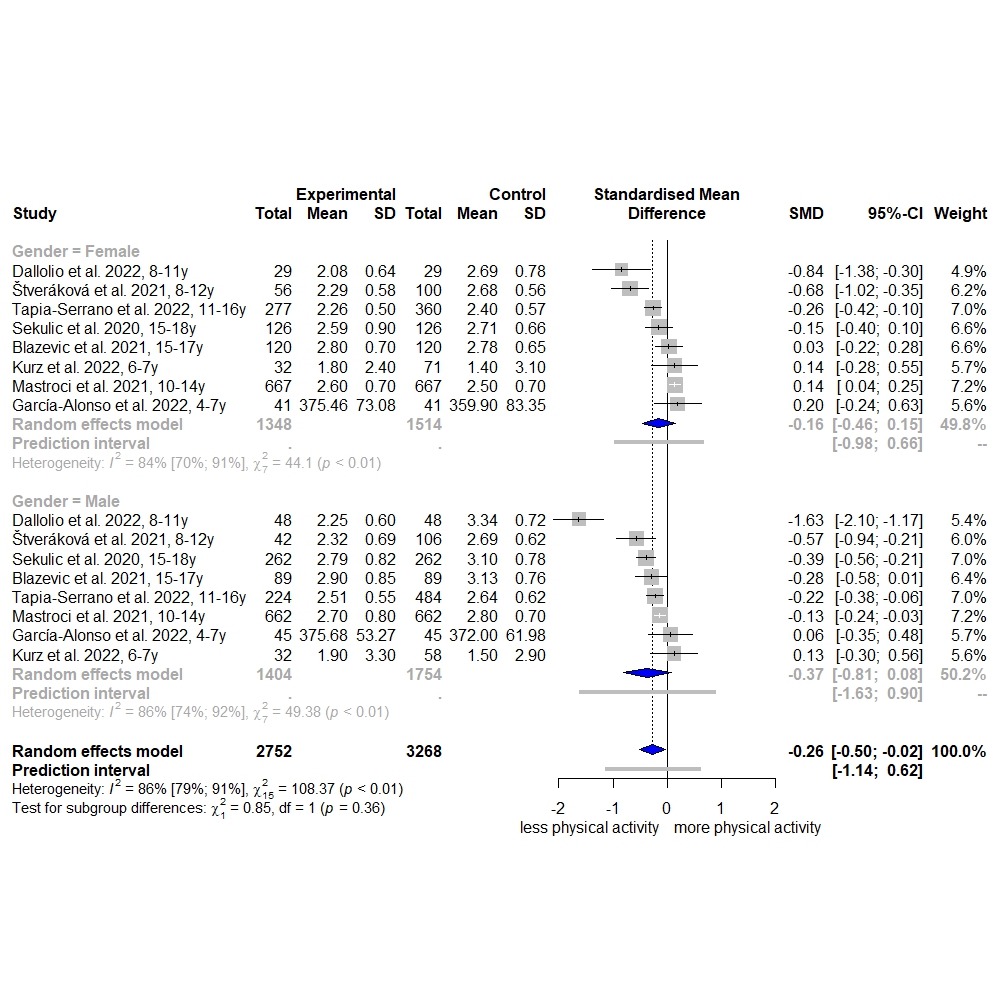

Supplement: Supplementary file 1 — Additional file 1: Table S1. PRISMA item checklist for systematic reviews. Table S2. Deviations from the systematic review protocol. Table S3. Searched websites of key organizations. Table S4. Search strategy. Table S5. Reasons for exclusion of studies from the systematic literature search, after full-text screening. Table S6. Data conversion. Table S7. Criteria for grading evidence according to Grading of Recommendations, Assessment, Development and Evaluations (GRADE). Table S8. Evidence profile for grading evidence according to Grading of Recommendations, Assessment, Development and Evaluations (GRADE). Table S9. Summary of effect estimates. Table S10. Meta-regression for total physical activity with categorical moderators. Table S11. Meta-regression for total physical activity with continuous moderators. Table S12. Meta-regression for moderate-to-vigorous physical activity with categorical moderators. Table S13. Meta-regression for moderate-to-vigorous physical activity with continuous moderators. Table S14. Sensitivity analysis for total physical activity. Table S15. Sensitivity analysis for moderate-to-vigorous physical activity. Table S16. Eggers’ test. Figure S1. PRISMA Flow Chart. Figure S2. Graphical distribution of the studies included. Figure S3. Traffic-light plots of the domain-level judgements for each individual result. Figure S4. Weighted-bar plots of the distribution of risk of bias judgements within each bias domain. Figure S5. Forest plot of changes in total physical activity comparing before and during COVID-19 pandemic, using Physical Activity Questionnaire for Children and Adolescents. Figure S6. Forest plot of changes in total physical activity comparing before and during COVID-19 pandemic, using accelerometer measurements. Figure S7. Forest plot of changes in female and male total physical activity comparing before and during COVID-19 pandemic. Figure S8. Forest plot of changes according to time course in total physical activity comparing be [file 12966_2023_1542_MOESM1_ESM.zip › S-Fig_7_ForestPlot_TPA_Female_Male.jpeg]

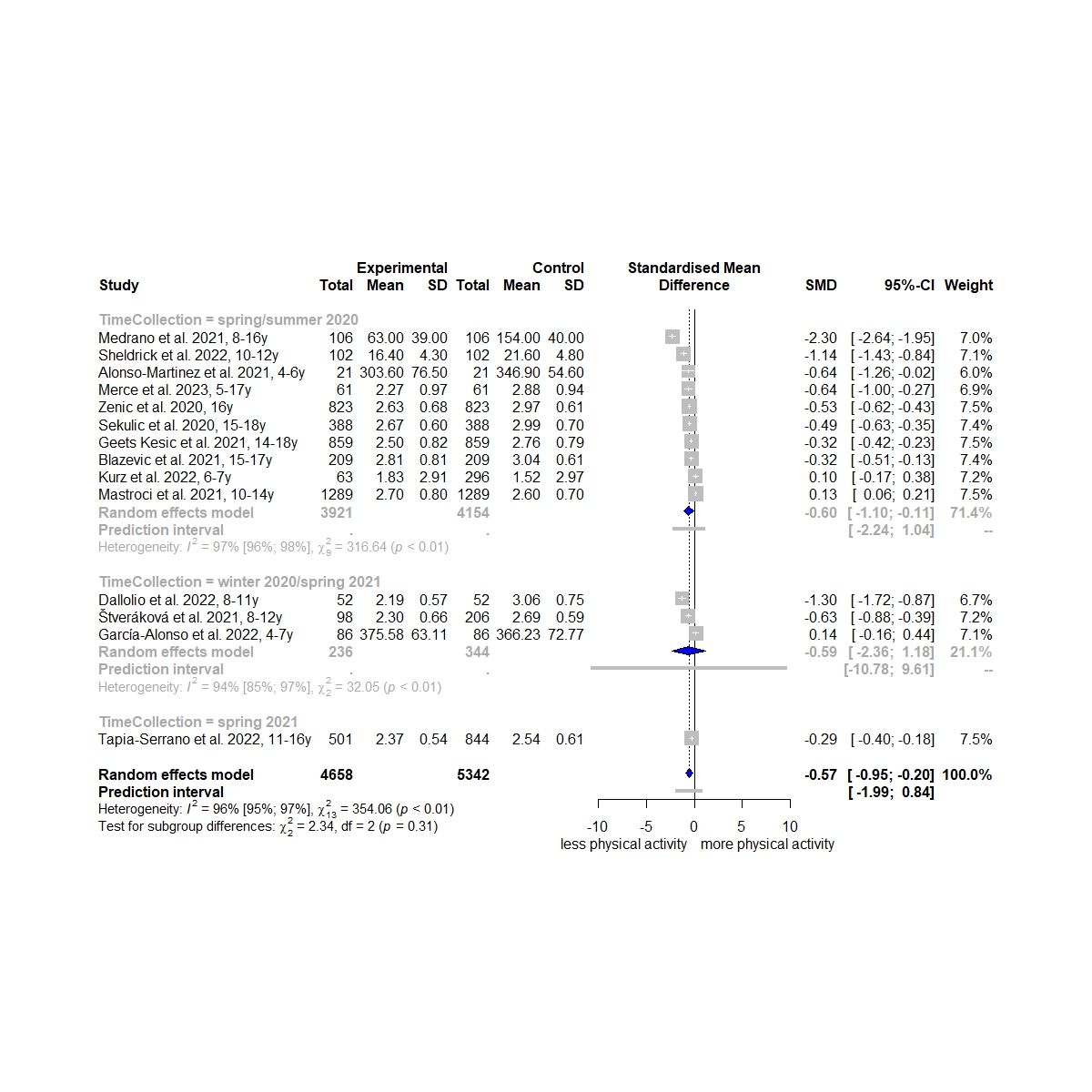

Supplement: Supplementary file 1 — Additional file 1: Table S1. PRISMA item checklist for systematic reviews. Table S2. Deviations from the systematic review protocol. Table S3. Searched websites of key organizations. Table S4. Search strategy. Table S5. Reasons for exclusion of studies from the systematic literature search, after full-text screening. Table S6. Data conversion. Table S7. Criteria for grading evidence according to Grading of Recommendations, Assessment, Development and Evaluations (GRADE). Table S8. Evidence profile for grading evidence according to Grading of Recommendations, Assessment, Development and Evaluations (GRADE). Table S9. Summary of effect estimates. Table S10. Meta-regression for total physical activity with categorical moderators. Table S11. Meta-regression for total physical activity with continuous moderators. Table S12. Meta-regression for moderate-to-vigorous physical activity with categorical moderators. Table S13. Meta-regression for moderate-to-vigorous physical activity with continuous moderators. Table S14. Sensitivity analysis for total physical activity. Table S15. Sensitivity analysis for moderate-to-vigorous physical activity. Table S16. Eggers’ test. Figure S1. PRISMA Flow Chart. Figure S2. Graphical distribution of the studies included. Figure S3. Traffic-light plots of the domain-level judgements for each individual result. Figure S4. Weighted-bar plots of the distribution of risk of bias judgements within each bias domain. Figure S5. Forest plot of changes in total physical activity comparing before and during COVID-19 pandemic, using Physical Activity Questionnaire for Children and Adolescents. Figure S6. Forest plot of changes in total physical activity comparing before and during COVID-19 pandemic, using accelerometer measurements. Figure S7. Forest plot of changes in female and male total physical activity comparing before and during COVID-19 pandemic. Figure S8. Forest plot of changes according to time course in total physical activity comparing be [file 12966_2023_1542_MOESM1_ESM.zip › S-Fig_8_ForestPlot_TPA_time.jpeg]

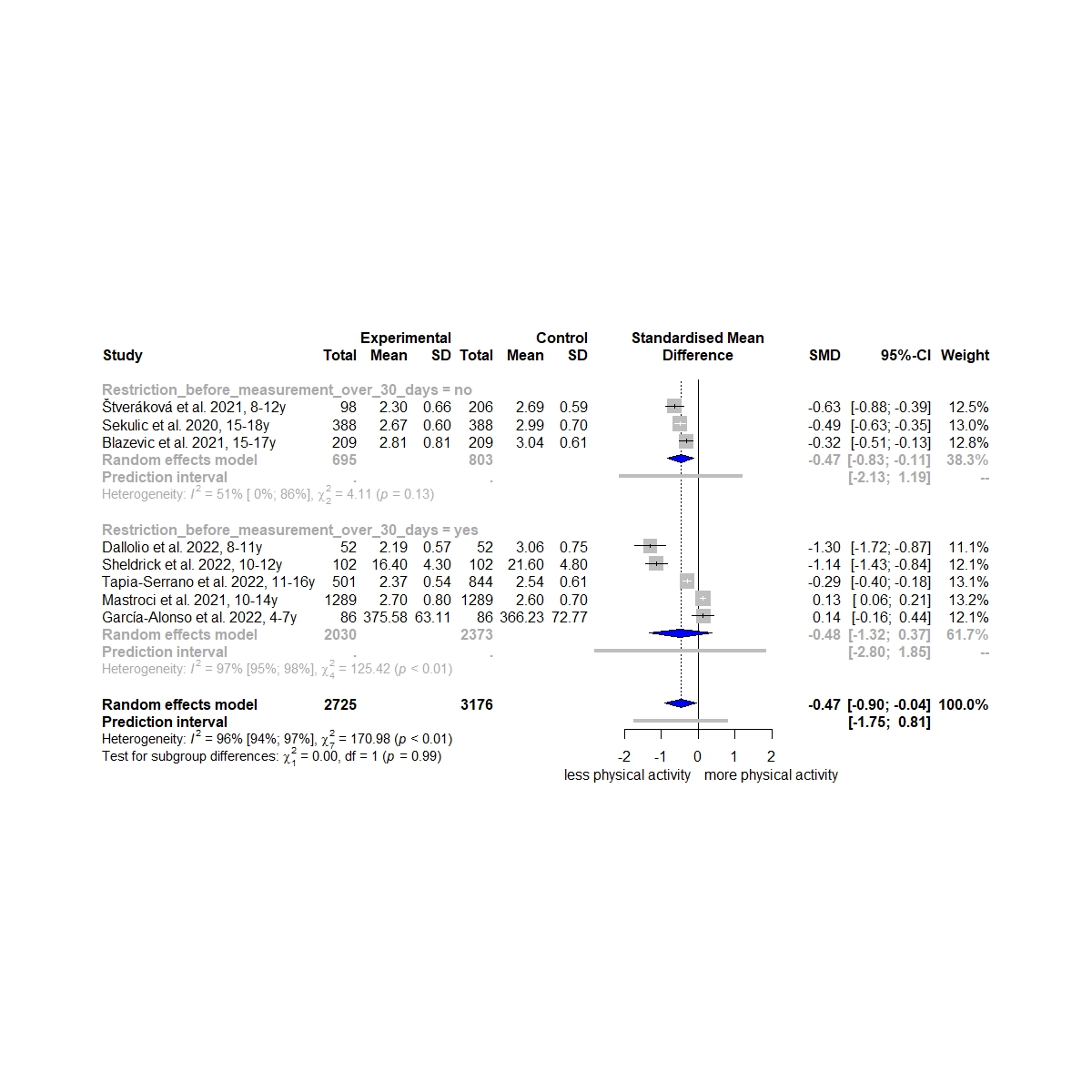

Supplement: Supplementary file 1 — Additional file 1: Table S1. PRISMA item checklist for systematic reviews. Table S2. Deviations from the systematic review protocol. Table S3. Searched websites of key organizations. Table S4. Search strategy. Table S5. Reasons for exclusion of studies from the systematic literature search, after full-text screening. Table S6. Data conversion. Table S7. Criteria for grading evidence according to Grading of Recommendations, Assessment, Development and Evaluations (GRADE). Table S8. Evidence profile for grading evidence according to Grading of Recommendations, Assessment, Development and Evaluations (GRADE). Table S9. Summary of effect estimates. Table S10. Meta-regression for total physical activity with categorical moderators. Table S11. Meta-regression for total physical activity with continuous moderators. Table S12. Meta-regression for moderate-to-vigorous physical activity with categorical moderators. Table S13. Meta-regression for moderate-to-vigorous physical activity with continuous moderators. Table S14. Sensitivity analysis for total physical activity. Table S15. Sensitivity analysis for moderate-to-vigorous physical activity. Table S16. Eggers’ test. Figure S1. PRISMA Flow Chart. Figure S2. Graphical distribution of the studies included. Figure S3. Traffic-light plots of the domain-level judgements for each individual result. Figure S4. Weighted-bar plots of the distribution of risk of bias judgements within each bias domain. Figure S5. Forest plot of changes in total physical activity comparing before and during COVID-19 pandemic, using Physical Activity Questionnaire for Children and Adolescents. Figure S6. Forest plot of changes in total physical activity comparing before and during COVID-19 pandemic, using accelerometer measurements. Figure S7. Forest plot of changes in female and male total physical activity comparing before and during COVID-19 pandemic. Figure S8. Forest plot of changes according to time course in total physical activity comparing be [file 12966_2023_1542_MOESM1_ESM.zip › S-Fig_9_ForestPlot_TPA_Restriction_30.jpeg]
